# Supplementary figures and images for: Identification and characterization of novel alphacoronaviruses in Tadarida brasiliensis (Chiroptera, Molossidae) from Argentina: insights into recombination as a mechanism favoring bat coronavirus cross-species transmission
Source: Microbiol Spectr. 2023 Sep 11;11(5):e02047-23. doi: 10.1128/spectrum.02047-23 (PMC10581097; doi:10.1128/spectrum.02047-23)

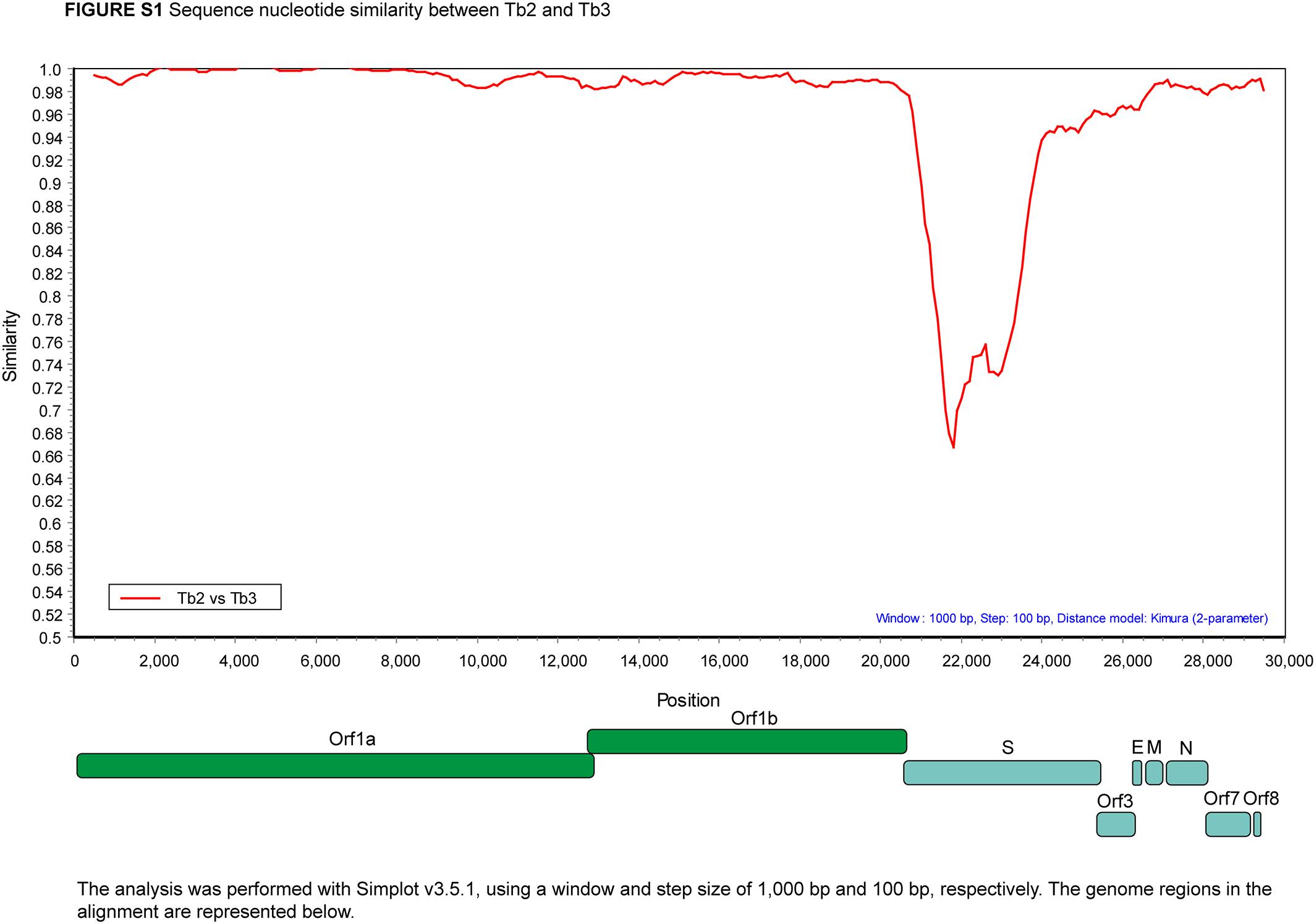

Supplement: Fig. S1 — Sequence nucleotide similarity between Tb2 and Tb3. [file spectrum.02047-23-s0001.tif]

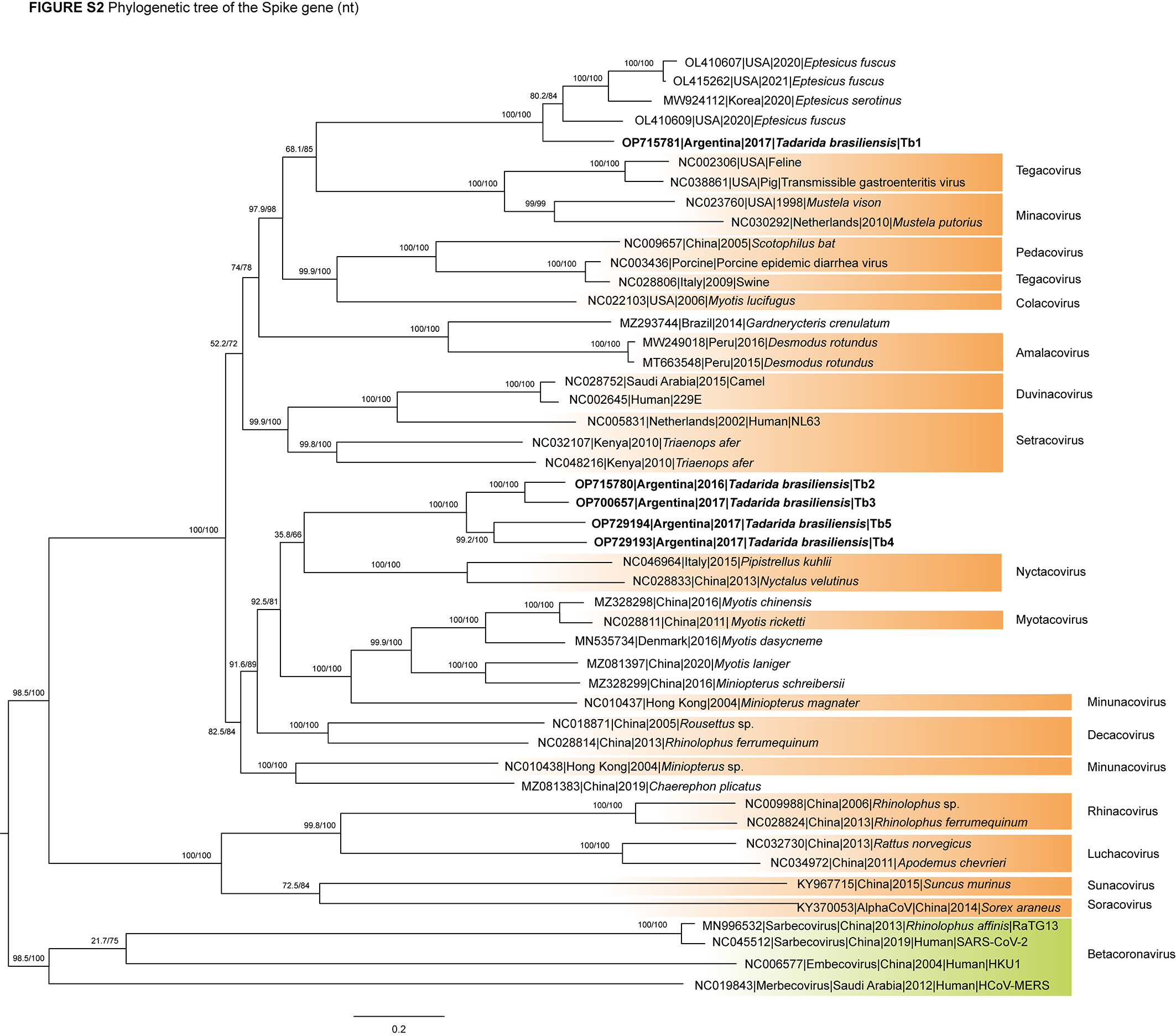

Supplement: Fig. S2 — Phylogenetic tree of the Spike gene (nt). [file spectrum.02047-23-s0002.tif]
