# Supplementary material for: Identification and characterization of novel alphacoronaviruses in Tadarida brasiliensis (Chiroptera, Molossidae) from Argentina: insights into recombination as a mechanism favoring bat coronavirus cross-species transmission
Source: Microbiol Spectr. 2023 Sep 11;11(5):e02047-23. doi: 10.1128/spectrum.02047-23 (PMC10581097; doi:10.1128/spectrum.02047-23)
Supplement: Table S1 — Sequences of primers designed using Primer3 Plus tool. [file spectrum.02047-23-s0003.docx]

**SUPPLEMENTARY TABLE S1** Sequences of primers designed using Primer3 Plus tool targeting the entire genome sequence of Tadarida brasiliensis bat alphacoronavirus 1 isolate Tb1

| Primer Pair Name | Forward | Reverse | Primers positions in the full genome (F / R ) | Expected PCR product size (bp) |
| --- | --- | --- | --- | --- |
| Bat_CoV_1 | 5′ ACCTGAACAAGCTGCATCCA 3′ | 5′ TCAACGTCATTGACATTAACACCA 3′ | 4,948–4,967/5,425–5,448 | 537 |
| Bat_CoV_2 | 5′ TCACTGCTGTTTCAAATGCACA 3′ | 5′ ACACCCACAACAATAGGACACT 3′ | 7,716–7,737/8,205–8,226 | 600 |
| Bat_CoV_3 | 5′ CGAATTCACACTTGCAGAGGT 3′ | 5′ GGGACACACAGAACCATCAT 3′ | 10,243–10,263/10,740–10,759 | 543 |
| Bat_CoV_4 | 5′ GGACAATCCTTTTGAGCATGCC 3′ | 5′ TACGACAGTGGTTTCAGGCC 3′ | 21,690–21,711/22,181–22,200 | 508 |
| Bat_CoV_5 | 5′ TGACGACAATGGTTTTGCCG 3′ | 5′ GGACATTGAGCACGATGATGC 3′ | 25,461–25,480/26,031–26,051 | 591 |

F: Forward primer

R: Reverse primer
