# Supplementary material for: Identification and characterization of novel alphacoronaviruses in Tadarida brasiliensis (Chiroptera, Molossidae) from Argentina: insights into recombination as a mechanism favoring bat coronavirus cross-species transmission
Source: Microbiol Spectr. 2023 Sep 11;11(5):e02047-23. doi: 10.1128/spectrum.02047-23 (PMC10581097; doi:10.1128/spectrum.02047-23)
Supplement: Table S2 — CoV sequences used for phylogenetic analysis. [file spectrum.02047-23-s0004.docx]

**SUPPLEMENTARY TABLE S2** CoV sequences used for phylogenetic analysis. Information was retrieved from Genbank.

| **Accession number** | **Genus** | **Subgenus** | **Country** | **Collection year** | **Host** |
| --- | --- | --- | --- | --- | --- |
| KY370053 | Alphacoronavirus | Soracovirus | China | 2014 | *Sorex araneus* |
| KY967715 | Alphacoronavirus | Sunacovirus | China | 2015 | *Suncus murinus* |
| MN535734 | Alphacoronavirus | unknown | Denmark | 2016 | *Myotis dasycneme* |
| MN996532 | Betacoronavirus | Sarbecovirus | China | 2013 | *Rhinolophus affinis* |
| MT663548 | Alphacoronavirus | Amalacovirus | Peru | 2015 | *Desmodus rotundus* |
| MW249018 | Alphacoronavirus | Amalacovirus | Peru | 2016 | *Desmodus rotundus* |
| MW924112 | Alphacoronavirus | unknown | Korea | 2020 | *Eptesicus sorotinus* |
| MZ081383 | Alphacoronavirus | unknown | China | 2019 | *Chaerephon plicatus* |
| MZ081397 | Alphacoronavirus | unknown | China | 2020 | *Myotis laniger* |
| MZ293744 | Alphacoronavirus | unknown | Brazil | 2014 | *Gardnerycteris crenulatum* |
| MZ328298 | Alphacoronavirus | unknown | China | 2016 | *Myotis chinensis* |
| MZ328299 | Alphacoronavirus | unknown | China | 2016 | *Miniopterus schreibersii* |
| NC002306 | Alphacoronavirus | Tegacovirus | U.S. | - | Feline |
| NC002645 | Alphacoronavirus | Duvinacovirus | - | - | Human |
| NC003436 | Alphacoronavirus | Pedacovirus | - | - | Porcine |
| NC005831 | Alphacoronavirus | Setracovirus | Netherlands | 2002 | Human |
| NC006577 | Betacoronavirus | Embecovirus | China | 2004 | Human |
| NC009657 | Alphacoronavirus | Pedacovirus | China | 2005 | *Scotophilus* |
| NC009988 | Alphacoronavirus | Rhinacovirus | China | 2006 | *Rhinolophus* |
| NC010437 | Alphacoronavirus | Minunacovirus | Hong Kong | 2004 | *Miniopterus magnater* |
| NC010438 | Alphacoronavirus | Minunacovirus | Hong Kong | 2004 | *Miniopterus* bat |
| NC018871 | Alphacoronavirus | Decacovirus | China | 2005 | *Rousettus* bat |
| NC019843 | Betacoronavirus | Merbecovirus | Saudi Arabia | 2012 | Human |
| NC022103 | Alphacoronavirus | Colacovirus | U.S. | 2006 | *Myotis lucifugus* |
| NC023760 | Alphacoronavirus | Minacovirus | U.S. | 1998 | Mustela vison |
| NC028752 | Alphacoronavirus | Duvinacovirus | Saudi Arabia | 2015 | Camel |
| NC028806 | Alphacoronavirus | Tegacovirus | Italy | 2009 | Swine |
| NC028811 | Alphacoronavirus | Myotacovirus | China | 2011 | *Myotis ricketti* |
| NC028814 | Alphacoronavirus | Decacovirus | China | 2013 | *Rhinolophus ferrumequinum* |
| NC028824 | Alphacoronavirus | Rhinacovirus | China | 2013 | *Rhinolophus ferrumequinum* |
| NC028833 | Alphacoronavirus | Nyctacovirus | China | 2013 | *Nyctalus velutinus* |
| NC030292 | Alphacoronavirus | Minacovirus | Netherlands | 2010 | *Mustela putorius* |
| NC032107 | Alphacoronavirus | Setracovirus | Kenia | 2010 | *Triaenops afer* |
| NC032730 | Alphacoronavirus | Luchacovirus | China | 2013 | *Rattus norvegicus* |
| NC034972 | Alphacoronavirus | Luchacovirus | China | 2011 | *Apodemus chevrieri* |
| NC038861 | Alphacoronavirus | Tegacovirus | U.S. | - | Pig |
| NC045512 | Betacoronavirus | Sarbecovirus | China | 2019 | Human |
| NC046964 | Alphacoronavirus | Nyctacovirus | Italy | 2015 | *Pipistrellus kuhlii* |
| NC048216 | Alphacoronavirus | Setracovirus | Kenya | 2010 | *Triaenops afer* |
| OL410607 | Alphacoronavirus | unknown | U.S. | 2020 | *Eptesicus fuscus* |
| OL410609 | Alphacoronavirus | unknown | U.S. | 2020 | *Eptesicus fuscus* |
| OL415262 | Alphacoronavirus | unknown | U.S. | 2021 | *Eptesicus fuscus* |
| OP700657 | Alphacoronavirus | unknown | Argentina | 2017 | *Tadarida brasiliensis* |
| OP715780 | Alphacoronavirus | unknown | Argentina | 2016 | *Tadarida brasiliensis* |
| OP715781 | Alphacoronavirus | unknown | Argentina | 2017 | *Tadarida brasiliensis* |
