# Supplementary material for: Identification and characterization of novel alphacoronaviruses in Tadarida brasiliensis (Chiroptera, Molossidae) from Argentina: insights into recombination as a mechanism favoring bat coronavirus cross-species transmission
Source: Microbiol Spectr. 2023 Sep 11;11(5):e02047-23. doi: 10.1128/spectrum.02047-23 (PMC10581097; doi:10.1128/spectrum.02047-23)
Supplement: Table S3 — AlphaCoVs sequences used in the recombination analysis. [file spectrum.02047-23-s0005.docx]

**SUPPLEMENTARY TABLE S3** AlphaCoVs sequences used in the recombination analysis.

Accession number, isolate name and information were retrieved from Genbank.

| **Accession number** | **Isolate** | **Subgenus** | **Country** | **Collection year** | **Host** |
| --- | --- | --- | --- | --- | --- |
| AF304460 | Human coronavirus 229E | Duvinacovirus | - | - | Human |
| AF353511 | Porcine epidemic diarrhea virus strain CV777 | Pedacovirus | - | - | Porcine |
| AJ271965 | Transmissible gastroenteritis virus genomic RNA | Tegacovirus | U.S. | - | Porcine |
| AY567487 | Human Coronavirus NL63 | Setracovirus | Netherlands | - | Human |
| DQ648858 | Bat coronavirus (BtCoV/512/2005) | Pedacovirus | China | - | Scotophilus bat |
| EF203064 | Bat coronavirus HKU2 strain HKU2/GD/430/2006 | Rhinacovirus | China | - | *Rhinolophus sinicus* |
| EF203065 | Bat coronavirus HKU2 strain HKU2/HK/46/2006 | Rhinacovirus | China | - | *Rhinolophus sinicus* |
| EU420137 | Bat coronavirus 1B strain AFCD307 | Minunacovirus | Hong Kong | 2006 | *Miniopterus* *pusillus* |
| EU420138 | Miniopterus bat coronavirus 1 | Minunacovirus | Hong Kong | 2006 | *Miniopterus* bat |
| EU420139 | Bat coronavirus HKU8 strain AFCD77 | Minunacovirus | Hong Kong | 2006 | *Miniopterus* bat |
| HM245925 | Mink coronavirus strain WD1127 | Minacovirus | U.S. | 1998 | Mustela vison |
| JQ989266 | Hipposideros bat coronavirus HKU10 isolate TT3A | Decacovirus | Hong Kong | 2006 | *Hipposideros* bat |
| JQ989270 | Rousettus bat coronavirus HKU10 isolate 183A | Decacovirus | China | 2005 | *Rousettus* bat |
| JQ989271 | Rousettus bat coronavirus HKU10 isolate 175A | Decacovirus | China | 2005 | *Rousettus* bat |
| JQ989272 | Hipposideros bat coronavirus HKU10 isolate TLC1343A | Decacovirus | Hong Kong | 2010 | *Hipposideros* bat |
| KF294380 | Lucheng Rn rat coronavirus isolate Lucheng-19 | Luchacovirus | China | 2013 | *Rattus norvegicus* |
| KF430219 | Bat coronavirus CDPHE15/USA/2006 | Colacovirus | U.S. | 2006 | *Myotis lucifugus* |
| KJ473795 | BtMf-AlphaCoV/AH2011 | Minunacovirus | China | 2011 | *Miniopterus fuliginosus* |
| KJ473796 | BtMf-AlphaCoV/JX2012 | Minunacovirus | China | 2012 | *Miniopterus fuliginosus* |
| KJ473797 | BtMf-AlphaCoV/GD2012 | Minunacovirus | China | 2012 | *Miniopterus fuliginosus* |
| KJ473798 | BtMf-AlphaCoV/HuB2013 | Minunacovirus | China | 2013 | *Miniopterus fuliginosus* |
| KJ473799 | BtMf-AlphaCoV/FJ2012 | Minunacovirus | China | 2012 | *Miniopterus fuliginosus* |
| KJ473800 | BtMf-AlphaCoV/HeN2013 | Minunacovirus | China | 2013 | *Miniopterus fuliginosus* |
| KJ473806 | BtMr-AlphaCoV/SAX2011 | Myotacovirus | China | 2011 | *Myotis ricketti* |
| KJ473807 | BtRf-AlphaCoV/HuB2013 | Decacovirus | China | 2013 | *Rhinolophus ferrumequinum* |
| KJ473808 | BtRf-AlphaCoV/YN2012 | Rhinacovirus | China | 2012 | *Rhinolophus ferrumequinum* |
| KJ473809 | BtNv-AlphaCoV/SC2013 | Nyctacovirus | China | 2013 | *Nyctalus velutinus* |
| KJ473810 | BtMs-AlphaCoV/GS2013 | Decacovirus | China | 2013 | *Myotis sp.* |
| KY073744 | NL63-related bat coronavirus strain BtKYNL63-9a | Setracovirus | Kenya | 2010 | *Triaenops afer* |
| KY073745 | NL63-related bat coronavirus strain BtKYNL63-9b | Setracovirus | Kenya | 2010 | *Triaenops afer* |
| KY073746 | NL63-related bat coronavirus strain BtKYNL63-15 | Setracovirus | Kenya | 2008 | *Triaenops afer* |
| KY073747 | 229E-related bat coronavirus strain BtKY229E-1 | Duvinacovirus | Kenya | 2009 | *Hipposideros sp.* |
| KY073748 | 229E-related bat coronavirus strain BtKY229E-8 | Duvinacovirus | Kenya | 2010 | *Hipposideros vittatus* |
| KY370053 | Common shrew coronavirus Tibet-2014 isolate Shrew-CoV/Tibet2014 | Soracovirus | China | 2014 | *Sorex araneus* |
| KY799179 | Myotis lucifugus coronavirus | Colacovirus | Canada | 2010 | *Myotis lucifugus* |
| KY967715 | Wencheng Sm shrew coronavirus isolate Xingguo-74 cds | Sunacovirus | China | 2015 | *Suncus murinus* |
| MG916901 | Bat coronavirus isolate BtCoV/Rh/YN2012 Rs3376 | unknown | China | 2012 | *Rhinolophus* Bats |
| MG916902 | Bat coronavirus isolate BtCoV/Rh/YN2012 Rs4125 | unknown | China | 2012 | *Rhinolophus* Bats |
| MG916903 | Bat coronavirus isolate BtCoV/Rh/YN2012 Rs4259 | unknown | China | 2013 | *Rhinolophus* Bats |
| MG916904 | Bat coronavirus isolate BtCoV/Rh/YN2012 Ra13591 | unknown | China | 2013 | *Rhinolophus* Bats |
| MG923574 | Bat alphacoronavirus isolate BtCoV/020 16/M.dau/FIN/2016 | Pedacovirus | Finland | 2016 | *Myotis daubentonii* |
| MH687935 | Alphacoronavirus sp. strain VZ AlphaCoV 16715 24 | unknown | Vietnam | 2014 | *Scotophilus kuhlii* |
| MH687939 | Alphacoronavirus sp. strain VZ AlphaCoV 16715 39 c2 | unknown | Vietnam | 2014 | *Scotophilus kuhlii* |
| MH687941 | Alphacoronavirus sp. strain VZ AlphaCoV 16715 47 c1 | unknown | Vietnam | 2014 | *Scotophilus kuhlii* |
| MH687953 | Alphacoronavirus sp. strain VZ AlphaCoV 16715 86 | unknown | Vietnam | 2014 | *Scotophilus kuhlii* |
| MH687954 | Alphacoronavirus sp. strain VZ AlphaCoV 16845 24 | unknown | Vietnam | 2014 | *Scotophilus kuhlii* |
| MH687956 | Alphacoronavirus sp. strain VZ AlphaCoV 16845 53 | unknown | Vietnam | 2014 | *Scotophilus kuhlii* |
| MH687957 | Alphacoronavirus sp. strain VZ AlphaCoV 16845 64 | unknown | Vietnam | 2014 | *Scotophilus kuhlii* |
| MH687964 | Alphacoronavirus sp. strain VZ AlphaCoV 20745 10 | unknown | Vietnam | 2015 | *Scotophilus kuhlii* |
| MH687965 | Alphacoronavirus sp. strain VZ AlphaCoV 20745 17 | unknown | Vietnam | 2015 | *Scotophilus kuhlii* |
| MH687966 | Alphacoronavirus sp. strain VZ AlphaCoV 20745 6 | unknown | Vietnam | 2015 | *Scotophilus kuhlii* |
| MH938448 | Alphacoronavirus Bat-CoV/P.kuhlii/Italy/206645-41/2011 | Nyctacovirus | Italy | 2011 | *Pipistrellus kuhlii* |
| MH938449 | Alphacoronavirus Bat-CoV/P.kuhlii/Italy/3398-19/2015 | Nyctacovirus | Italy | 2015 | *Pipistrellus kuhlii* |
| MH938450 | Alphacoronavirus Bat-CoV/P.kuhlii/Italy/206679-3/2010 | Nyctacovirus | Italy | 2010 | *Pipistrellus kuhlii* |
| MK211369 | Coronavirus BtSk-AlphaCoV/GX2018A | Pedacovirus | China | 2017 | *Scotophilus kuhlii* |
| MK211370 | Coronavirus BtSk-AlphaCoV/GX2018B | Pedacovirus | China | 2017 | *Scotophilus kuhlii* |
| MK211371 | Coronavirus BtSk-AlphaCoV/GX2018C | Pedacovirus | China | 2017 | *Scotophilus kuhlii* |
| MK211372 | Coronavirus BtSk-AlphaCoV/GX2018D | Pedacovirus | China | 2017 | *Scotophilus kuhlii* |
| MK211373 | Coronavirus BtRs-AlphaCoV/YN2018 | unknown | China | 2017 | *Cynopterus sphinx* |
| MK472067 | Alphacoronavirus sp. isolate WA1087 | Pedacovirus | Australia | 2018 | microbat |
| MK472068 | Alphacoronavirus sp. isolate WA2028 | Nyctacovirus | Australia | 2018 | microbat |
| MK472070 | Alphacoronavirus sp. isolate WA3607 | Decacovirus | Australia | 2018 | microbat |
| MK720944 | Tylonycteris bat coronavirus HKU33 strain GZ151867 | Nyctacovirus | China | 2015 | *Tylonycteris robustula* |
| MK720945 | Rhinolophus bat coronavirus HKU32 strain TLC26A | Decacovirus | Hong Kong | 2015 | *Rhinolophus sinicus* |
| MN065811 | Bat alphacoronavirus strain BtCoV/008 16/M.bra/FIN/2016 | unknown | Finland | 2016 | *Myotis brandtii* |
| MN482242 | MAG: Bat coronavirus isolate BtCoV/B40-5/P.pyg/DK/2013 | unknown | Denmark | 2013 | *Pipistrellus pygmaeus* |
| MN535732 | MAG: Bat coronavirus isolate BtCoV/13585-58/M.dau/DK/2014 | unknown | Denmark | 2014 | *Myotis daubentonii* |
| MN535733 | MAG: Bat coronavirus isolate BtCoV/OV-157/M.dau/DK/2018 | unknown | Denmark | 2018 | *Myotis daubentonii* |
| MN535734 | MAG: Bat coronavirus isolate BtCoV/18802-1/M.das/DK/2016 | unknown | Denmark | 2016 | *Myotis dasycneme* |
| MN611517 | Rousettus aegyptiacus bat coronavirus 229E-related isolate 5425 | Duvinacovirus | Kenya | 2018 | *Rousettus aegyptiacus* |
| MN611518 | Miniopterus pusillus bat coronavirus HKU8-related isolate 6610 | Minunacovirus | China | 2018 | *Miniopterus pusillus* |
| MN611521 | Scotophilus kuhlii bat coronavirus 512-related isolate HK140714 | Pedacovirus | China | 2018 | *Scotophilus kuhlii* |
| MN611522 | Rhinolophus affinis bat coronavirus HKU2-related isolate 160660 | Rhinacovirus | China | 2018 | *Rhinolophus affinis* |
| MN611523 | Hipposideros pomona bat coronavirus HKU10-related isolate 160942 | Decacovirus | China | 2018 | *Hipposideros pomona* |
| MN611524 | Miniopterus schreibersii bat coronavirus 1-related isolate 161454 | Minunacovirus | China | 2018 | *Miniopterus schreibersii* |
| MN611525 | Hipposideros pomona bat coronavirus CHB25 isolate CHB0025 | Decacovirus | China | 2018 | *Hipposideros larvatus* |
| MT663548 | Bat alphacoronavirus isolate AMA L F | Amalacovirus | Peru | 2015 | *Desmodus rotundus* |
| MT747186 | Swine acute diarrhea syndrome coronavirus isolate SADS-CoV/CN/GDDCD/2017 | Rhinacovirus | China | 2017 | Swine |
| MW249018 | Bat coronavirus isolate DesRot/Peru/HUA4 F DrCoV | Amalacovirus | Peru | 2016 | *Desmodus rotundus* |
| MW924112 | Alphacoronavirus HCQD-2020 | unknown | South Korea | 2020 | *Eptesicus sorotinus* |
| MZ081383 | Alphacoronavirus sp. strain bat/Yunnan/CpYN11/2019 | unknown | China | 2019 | *Chaerephon plicatus* |
| MZ081384 | Alphacoronavirus sp. strain bat/Yunnan/HcYN26/2020 | unknown | China | 2020 | *Hipposideros cineraceus* |
| MZ081387 | Alphacoronavirus sp. strain bat/Yunnan/RmYN21/2020 | unknown | China | 2020 | *Rhinolophus malayanus* |
| MZ081388 | Alphacoronavirus sp. strain bat/Yunnan/HlYN23/2020 | unknown | China | 2020 | *Hipposideros larvatus* |
| MZ081389 | Alphacoronavirus sp. strain bat/Yunnan/HlYN10/2019 | unknown | China | 2019 | *Hipposideros larvatus* |
| MZ081390 | Alphacoronavirus sp. strain bat/Yunnan/HpYN13/2019 | unknown | China | 2019 | *Hipposideros pomona* |
| MZ081391 | Alphacoronavirus sp. strain bat/Yunnan/RmYN22/2020 | unknown | China | 2020 | *Rhinolophus malayanus* |
| MZ081392 | Alphacoronavirus sp. strain bat/Yunnan/McYN19/2020 | unknown | China | 2020 | *Murina cyclotis* |
| MZ081396 | Alphacoronavirus sp. strain bat/Yunnan/HlYN18/2020 | unknown | China | 2020 | *Hipposideros larvatus* |
| MZ081397 | Alphacoronavirus sp. strain bat/Yunnan/MlYN20/2020 | unknown | China | 2020 | *Myotis laniger* |
| MZ081398 | Alphacoronavirus sp. strain bat/Yunnan/MlYN15/2020 | unknown | China | 2020 | *Myotis laniger* |
| MZ081399 | Alphacoronavirus sp. strain bat/Yunnan/RsYN25/2020 | unknown | China | 2020 | *Rhinolophus stheno* |
| MZ218052 | MAG: Bat coronavirus isolate BtCoV/21164-6-alt/M.dau/DK/2015 | unknown | Denmark | 2015 | *Myotis daubentonii* |
| MZ218060 | MAG: Bat coronavirus isolate BtCoV/7542-55/P.pyg/DK/2014 | unknown | Denmark | 2014 | *Pipistrellus pygmaeus* |
| MZ293744 | Mimon bat coronavirus isolate PREDICT/PDF-3316 | unknown | Brazil | 2014 | *Gardnerycteris crenulatum* |
| MZ328298 | Jingmen Myotis chinensis alphacoronavirus 1 | unknown | China | 2016 | *Myotis chinensis* |
| MZ328299 | Jingmen Miniopterus schreibersii alphacoronavirus 1 | unknown | China | 2016 | *Miniopterus schreibersii* |
| MZ328300 | Jingmen Miniopterus schreibersii alphacoronavirus 2 | unknown | China | 2016 | *Miniopterus schreibersii* |
| NC002306 | Feline infectious peritonitis virus | Tegacovirus | U.S. | - | Feline |
| NC028752 | Camel alphacoronavirus isolate camel/Riyadh/Ry141/2015 | Duvinacovirus | Saudi Arabia | 2015 | Camel |
| NC028806 | Swine enteric coronavirus strain Italy/213306/2009 | Tegacovirus | Italy | 2009 | Swine |
| NC030292 | Ferret coronavirus isolate FRCoV-NL-2010 | Minacovirus | Netherlands | 2010 | *Mustela putorius* |
| NC034972 | Coronavirus AcCoV-JC34 | Luchacovirus | China | 2011 | *Apodemus chevrieri* |
| OK287352 | Alphacoronavirus sp. isolate Yunnan/Hp JC8xc/2020 | unknown | China | 2020 | *Hipposideros pomona* |
| OK287353 | Alphacoronavirus sp. isolate Yunnan Rs CX18c 2020 | unknown | China | 2020 | *Rhinolophus sinicus* |
| OL410607 | Eptesicus bat coronavirus strain 15712 | unknown | U.S. | 2020 | *Eptesicus fuscus* |
| OL410609 | Eptesicus bat coronavirus strain 16964 | unknown | U.S. | 2020 | *Eptesicus fuscus* |
| OL415262 | Eptesicus bat coronavirus strain 15593 | unknown | U.S. | 2021 | *Eptesicus fuscus* |
| OL956935 | Bat alphacoronavirus isolate batCoV/MinFul/2018/SriLanka | unknown | Sri Lanka | 2018 | *Miniopterus fuliginosus* |
| OM030318 | Jingmen Myotis ricketti alphacoronavirus 1 isolate JSB DaZu | unknown | China | 2016 | *Myotis ricketti* |
| ON325309 | Bat alphacoronavirus isolate BatCoV/M.myotis/Switzerland/2019 | unknown | Switzerland | 2019 | *Myotis myotis* |
| ON378803 | MAG: Alphacoronavirus sp. isolate BatCoV B20-97 | unknown | South Korea | 2020 | *Rhinolophus ferrumequinum* |
| ON378804 | MAG: Alphacoronavirus sp. isolate BatCoV B20-104-1 | unknown | South Korea | 2020 | *Myotis petax* |
| ON378805 | MAG: Alphacoronavirus sp. isolate BatCoV B20-104-2 | unknown | South Korea | 2020 | *Myotis petax* |
| ON378806 | MAG: Alphacoronavirus sp. isolate BatCoV 20-177 | unknown | South Korea | 2020 | *Myotis macrodactylus* |
| OP700657 | MAG: Tadarida brasiliensis bat alphacoronavirus 2 isolate Tb3 | unknown | Argentina | 2017 | *Tadarida brasiliensis* |
| OP715780 | MAG: Tadarida brasiliensis bat alphacoronavirus 2 isolate Tb2 | unknown | Argentina | 2016 | *Tadarida brasiliensis* |
| OP715781 | MAG: Tadarida brasiliensis bat alphacoronavirus 1 isolate Tb1 | unknown | Argentina | 2017 | *Tadarida brasiliensis* |

MAG: Metagenomic assembled genome
