# Supplementary material for: Identification and characterization of novel alphacoronaviruses in Tadarida brasiliensis (Chiroptera, Molossidae) from Argentina: insights into recombination as a mechanism favoring bat coronavirus cross-species transmission
Source: Microbiol Spectr. 2023 Sep 11;11(5):e02047-23. doi: 10.1128/spectrum.02047-23 (PMC10581097; doi:10.1128/spectrum.02047-23)
Supplement: Table S4 — Pairwise amino acid comparisons of the replicase polyprotein pp1ab conserved domains of the AlphaCoVs sequences. [file spectrum.02047-23-s0006.docx]

**SUPPLEMENTARY TABLE S4** Pairwise amino acid comparisons of the replicase polyprotein pp1ab conserved domains of the AlphaCoVs sequences.

| **Species 1** | **Species 2** | **% Identity in pp1ab conserved domains** |
| --- | --- | --- |
| MW924112 Alphacoronavirus Korea 2020 Eptesicus serotinus HCQD2020 | OL415262 Alphacoronavirus U.S. 2021 Eptesicus fuscus | 99.9% |
| OL410607 Alphacoronavirus U.S. 2020 Eptesicus fuscus | OL415262 Alphacoronavirus U.S. 2021 Eptesicus fuscus | 99.8% |
| **OP700657 Tadarida brasilensis bat alphacoronavirus 2 isolate Tb3** | **OP715780 Tadarida brasilensis bat alphacoronavirus 2 isolate Tb2** | **99.8%** |
| OL410609 Alphacoronavirus U.S. 2020 Eptesicus fuscus | OL415262 Alphacoronavirus U.S. 2021 Eptesicus fuscus | 99.8% |
| MW924112 Alphacoronavirus Korea 2020 Eptesicus serotinus HCQD2020 | OL410607 Alphacoronavirus U.S. 2020 Eptesicus fuscus | 99.7% |
| MW924112 Alphacoronavirus Korea 2020 Eptesicus serotinus HCQD2020 | OL410609 Alphacoronavirus U.S. 2020 Eptesicus fuscus | 99.7% |
| MN996532 Betacoronavirus Sarbecovirus China 2013 Rhinolophus affinis RaTG13 | NC045512 Betacoronavirus Sarbecovirus China 2019 Human SARS CoV 2 | 99.6% |
| MZ328298 Alphacoronavirus China 2016 Myotis chinensis | NC028811 Alphacoronavirus Myotacovirus China 2011 Myotis ricketti SAX2011 | 99.6% |
| OL410607 Alphacoronavirus U.S. 2020 Eptesicus fuscus | OL410609 Alphacoronavirus U.S. 2020 Eptesicus fuscus | 99.6% |
| MT663548 Alphacoronavirus Peru 2015 Desmodus rotundus | MW249018 Coronavirinae Peru 2016 Desmodus rotundus | 99.4% |
| NC009988 Alphacoronavirus Rhinacovirus China 2006 Rhinolophus bat HKU2 | NC028824 Alphacoronavirus Rhinacovirus China 2013 Rhinolophus ferrumequinum YN2012 | 99.3% |
| NC028806 Alphacoronavirus Tegacovirus Italy 2009 Swine | NC038861 Alphacoronavirus Tegacovirus U.S. Pig Transmissible gastroenteritis virus | 98.6% |
| NC002645 Alphacoronavirus Duvinacovirus Human coronavirus 229E | NC028752 Alphacoronavirus Duvinacovirus Saudi Arabia 2015 Camel | 97.6% |
| NC002306 Alphacoronavirus Tegacovirus U.S. Feline | NC038861 Alphacoronavirus Tegacovirus U.S. Pig Transmissible gastroenteritis virus | 97.3% |
| MZ081397 Alphacoronavirus China 2020 Myotis laniger | MZ328299 Alphacoronavirus China 2016 Miniopterus schreibersii | 96.8% |
| NC002306 Alphacoronavirus Tegacovirus U.S. Feline | NC028806 Alphacoronavirus Tegacovirus Italy 2009 Swine | 96.3% |
| MN535734 Coronavirinae Denmark 2016 Myotis dasycneme | NC028811 Alphacoronavirus Myotacovirus China 2011 Myotis ricketti SAX2011 | 96.2% |
| MN535734 Coronavirinae Denmark 2016 Myotis dasycneme | MZ328298 Alphacoronavirus China 2016 Myotis chinensis | 96.1% |
| **OL415262 Alphacoronavirus U.S. 2021 Eptesicus fuscus** | **OP715781 Tadarida brasiliensis bat alphacoronavirus 1 isolate Tb1** | **95.8%** |
| **OL410607 Alphacoronavirus U.S. 2020 Eptesicus fuscus** | **OP715781 Tadarida brasiliensis bat alphacoronavirus 1 isolate Tb1** | **95.7%** |
| **OL410609 Alphacoronavirus U.S. 2020 Eptesicus fuscus** | **OP715781 Tadarida brasiliensis bat alphacoronavirus 1 isolate Tb1** | **95.6%** |
| **MW924112 Alphacoronavirus Korea 2020 Eptesicus serotinus HCQD2020** | **OP715781 Tadarida brasiliensis bat alphacoronavirus 1 isolate Tb1** | **95.6%** |
| MW249018 Coronavirinae Peru 2016 Desmodus rotundus | MZ293744 Alphacoronavirus Brazil 2014 Gardnerycteris crenulatum bat | 95.0% |
| MT663548 Alphacoronavirus Peru 2015 Desmodus rotundus | MZ293744 Alphacoronavirus Brazil 2014 Gardnerycteris crenulatum bat | 94.9% |
| NC032730 Alphacoronavirus Luchacovirus China 2013 Rattus norvegicus | NC034972 Alphacoronavirus Luchacovirus China 2011 Apodemus chevrieri rat AcCoV JC34 | 94.7% |
| NC005831 Alphacoronavirus Setracovirus Netherlands 2002 Human NL63 | NC032107 Alphacoronavirus Setracovirus Kenia 2010 Triaenops afer NL63 related bat coronavirus BtKYNL63 9a | 92.8% |
| MN535734 Coronavirinae Denmark 2016 Myotis dasycneme | MZ081397 Alphacoronavirus China 2020 Myotis laniger | 92.6% |
| MZ328299 Alphacoronavirus China 2016 Miniopterus schreibersii | NC028811 Alphacoronavirus Myotacovirus China 2011 Myotis ricketti SAX2011 | 92.3% |
| MZ081397 Alphacoronavirus China 2020 Myotis laniger | NC028811 Alphacoronavirus Myotacovirus China 2011 Myotis ricketti SAX2011 | 92.3% |
| MZ328298 Alphacoronavirus China 2016 Myotis chinensis | MZ328299 Alphacoronavirus China 2016 Miniopterus schreibersii | 92.3% |
| MZ081397 Alphacoronavirus China 2020 Myotis laniger | MZ328298 Alphacoronavirus China 2016 Myotis chinensis | 92.3% |
| MN535734 Coronavirinae Denmark 2016 Myotis dasycneme | MZ328299 Alphacoronavirus China 2016 Miniopterus schreibersii | 92.2% |
| NC023760 Alphacoronavirus Minacovirus U.S. 1998 Mustela vison | NC030292 Alphacoronavirus Minacovirus Netherlands 2010 Mustela putorius ferret | 91.2% |
| NC018871 Alphacoronavirus Decacovirus China 2005 Rousettus bat HKU10 | NC028814 Alphacoronavirus Decacovirus China 2013 Rhinolophus ferrumequinum HuB2013 | 89.7% |
| NC032107 Alphacoronavirus Setracovirus Kenia 2010 Triaenops afer NL63 related bat coronavirus BtKYNL63 9a | NC048216 Alphacoronavirus Setracovirus Kenya 2010 Triaenops afer bat NL63 related bat coronavirus BtKYNL63 9b | 89.0% |
| NC028833 Alphacoronavirus Nyctacovirus China 2013 Nyctalus velutinus bat | NC046964 Alphacoronavirus Nyctacovirus Italy 2015 Pipistrellus kuhlii | 88.2% |
| NC005831 Alphacoronavirus Setracovirus Netherlands 2002 Human NL63 | NC048216 Alphacoronavirus Setracovirus Kenya 2010 Triaenops afer bat NL63 related bat coronavirus BtKYNL63 9b | 86.9% |
| NC003436 Alphacoronavirus Pedacovirus Porcine | NC009657 Alphacoronavirus Pedacovirus China 2005 Scotophilus bat | 85.9% |
| NC010437 Alphacoronavirus Minunacovirus Hong Kong 2004 Miniopterus magnater | NC010438 Alphacoronavirus Minunacovirus Hong Kong 2004 Miniopterus bat HKU8 | 85.8% |
| NC028752 Alphacoronavirus Duvinacovirus Saudi Arabia 2015 Camel | NC048216 Alphacoronavirus Setracovirus Kenya 2010 Triaenops afer bat NL63 related bat coronavirus BtKYNL63 9b | 84.4% |
| **NC028814 Alphacoronavirus Decacovirus China 2013 Rhinolophus ferrumequinum HuB2013** | **OP715780 Tadarida brasilensis bat alphacoronavirus 2 isolate Tb2** | **84.2%** |
| **NC028814 Alphacoronavirus Decacovirus China 2013 Rhinolophus ferrumequinum HuB2013** | **OP700657 Tadarida brasilensis bat alphacoronavirus 2 isolate Tb3** | **84.2%** |
| NC023760 Alphacoronavirus Minacovirus U.S. 1998 Mustela vison | NC038861 Alphacoronavirus Tegacovirus U.S. Pig Transmissible gastroenteritis virus | 84.0% |
| NC002645 Alphacoronavirus Duvinacovirus Human coronavirus 229E | NC048216 Alphacoronavirus Setracovirus Kenya 2010 Triaenops afer bat NL63 related bat coronavirus BtKYNL63 9b | 83.9% |
| NC030292 Alphacoronavirus Minacovirus Netherlands 2010 Mustela putorius ferret | NC038861 Alphacoronavirus Tegacovirus U.S. Pig Transmissible gastroenteritis virus | 83.9% |
| NC023760 Alphacoronavirus Minacovirus U.S. 1998 Mustela vison | NC028806 Alphacoronavirus Tegacovirus Italy 2009 Swine | 83.8% |
| NC028752 Alphacoronavirus Duvinacovirus Saudi Arabia 2015 Camel | NC032107 Alphacoronavirus Setracovirus Kenia 2010 Triaenops afer NL63 related bat coronavirus BtKYNL63 9a | 83.8% |
| NC002306 Alphacoronavirus Tegacovirus U.S. Feline | NC030292 Alphacoronavirus Minacovirus Netherlands 2010 Mustela putorius ferret | 83.6% |
| NC028806 Alphacoronavirus Tegacovirus Italy 2009 Swine | NC030292 Alphacoronavirus Minacovirus Netherlands 2010 Mustela putorius ferret | 83.5% |
| NC002306 Alphacoronavirus Tegacovirus U.S. Feline | NC023760 Alphacoronavirus Minacovirus U.S. 1998 Mustela vison | 83.5% |
| NC002645 Alphacoronavirus Duvinacovirus Human coronavirus 229E | NC032107 Alphacoronavirus Setracovirus Kenia 2010 Triaenops afer NL63 related bat coronavirus BtKYNL63 9a | 83.5% |
| NC010438 Alphacoronavirus Minunacovirus Hong Kong 2004 Miniopterus bat HKU8 | NC028814 Alphacoronavirus Decacovirus China 2013 Rhinolophus ferrumequinum HuB2013 | 83.4% |
| NC010438 Alphacoronavirus Minunacovirus Hong Kong 2004 Miniopterus bat HKU8 | NC018871 Alphacoronavirus Decacovirus China 2005 Rousettus bat HKU10 | 83.2% |
| NC028814 Alphacoronavirus Decacovirus China 2013 Rhinolophus ferrumequinum HuB2013 | NC048216 Alphacoronavirus Setracovirus Kenya 2010 Triaenops afer bat NL63 related bat coronavirus BtKYNL63 9b | 83.2% |
| NC010437 Alphacoronavirus Minunacovirus Hong Kong 2004 Miniopterus magnater | NC028814 Alphacoronavirus Decacovirus China 2013 Rhinolophus ferrumequinum HuB2013 | 82.9% |
| **NC003436 Alphacoronavirus Pedacovirus Porcine** | **OP700657 Tadarida brasilensis bat alphacoronavirus 2 isolate Tb3** | **82.8%** |
| **NC003436 Alphacoronavirus Pedacovirus Porcine** | **OP715780 Tadarida brasilensis bat alphacoronavirus 2 isolate Tb2** | **82.8%** |
| NC028814 Alphacoronavirus Decacovirus China 2013 Rhinolophus ferrumequinum HuB2013 | NC046964 Alphacoronavirus Nyctacovirus Italy 2015 Pipistrellus kuhlii | 82.5% |
| NC003436 Alphacoronavirus Pedacovirus Porcine | NC028814 Alphacoronavirus Decacovirus China 2013 Rhinolophus ferrumequinum HuB2013 | 82.5% |
| NC003436 Alphacoronavirus Pedacovirus Porcine | NC018871 Alphacoronavirus Decacovirus China 2005 Rousettus bat HKU10 | 82.4% |
| **NC018871 Alphacoronavirus Decacovirus China 2005 Rousettus bat HKU10** | **OP700657 Tadarida brasilensis bat alphacoronavirus 2 isolate Tb3** | **82.4%** |
| **NC018871 Alphacoronavirus Decacovirus China 2005 Rousettus bat HKU10** | **OP715780 Tadarida brasilensis bat alphacoronavirus 2 isolate Tb2** | **82.4%** |
| NC028814 Alphacoronavirus Decacovirus China 2013 Rhinolophus ferrumequinum HuB2013 | NC032107 Alphacoronavirus Setracovirus Kenia 2010 Triaenops afer NL63 related bat coronavirus BtKYNL63 9a | 82.4% |
| **NC022103 Alphacoronavirus Colacovirus U.S. 2006 Myotis lucifugus** | **OP700657 Tadarida brasilensis bat alphacoronavirus 2 isolate Tb3** | **82.3%** |
| **NC022103 Alphacoronavirus Colacovirus U.S. 2006 Myotis lucifugus** | **OP715780 Tadarida brasilensis bat alphacoronavirus 2 isolate Tb2** | **82.3%** |
| NC018871 Alphacoronavirus Decacovirus China 2005 Rousettus bat HKU10 | NC046964 Alphacoronavirus Nyctacovirus Italy 2015 Pipistrellus kuhlii | 81.9% |
| NC003436 Alphacoronavirus Pedacovirus Porcine | NC022103 Alphacoronavirus Colacovirus U.S. 2006 Myotis lucifugus | 81.9% |
| NC005831 Alphacoronavirus Setracovirus Netherlands 2002 Human NL63 | NC028752 Alphacoronavirus Duvinacovirus Saudi Arabia 2015 Camel | 81.9% |
| MZ328298 Alphacoronavirus China 2016 Myotis chinensis | NC028814 Alphacoronavirus Decacovirus China 2013 Rhinolophus ferrumequinum HuB2013 | 81.9% |
| NC028811 Alphacoronavirus Myotacovirus China 2011 Myotis ricketti SAX2011 | NC028814 Alphacoronavirus Decacovirus China 2013 Rhinolophus ferrumequinum HuB2013 | 81.8% |
| **NC046964 Alphacoronavirus Nyctacovirus Italy 2015 Pipistrellus kuhlii** | **OP715780 Tadarida brasilensis bat alphacoronavirus 2 isolate Tb2** | **81.8%** |
| **NC010438 Alphacoronavirus Minunacovirus Hong Kong 2004 Miniopterus bat HKU8** | **OP715780 Tadarida brasilensis bat alphacoronavirus 2 isolate Tb2** | **81.8%** |
| MZ081397 Alphacoronavirus China 2020 Myotis laniger | NC028814 Alphacoronavirus Decacovirus China 2013 Rhinolophus ferrumequinum HuB2013 | 81.8% |
| **NC046964 Alphacoronavirus Nyctacovirus Italy 2015 Pipistrellus kuhlii** | **OP700657 Tadarida brasilensis bat alphacoronavirus 2 isolate Tb3** | **81.7%** |
| MZ328299 Alphacoronavirus China 2016 Miniopterus schreibersii | NC028814 Alphacoronavirus Decacovirus China 2013 Rhinolophus ferrumequinum HuB2013 | 81.7% |
| **NC010438 Alphacoronavirus Minunacovirus Hong Kong 2004 Miniopterus bat HKU8** | **OP700657 Tadarida brasilensis bat alphacoronavirus 2 isolate Tb3** | **81.7%** |
| **NC048216 Alphacoronavirus Setracovirus Kenya 2010 Triaenops afer bat NL63 related bat coronavirus BtKYNL63 9b** | **OP715780 Tadarida brasilensis bat alphacoronavirus 2 isolate Tb2** | **81.7%** |
| **NC048216 Alphacoronavirus Setracovirus Kenya 2010 Triaenops afer bat NL63 related bat coronavirus BtKYNL63 9b** | **OP700657 Tadarida brasilensis bat alphacoronavirus 2 isolate Tb3** | **81.7%** |
| NC028814 Alphacoronavirus Decacovirus China 2013 Rhinolophus ferrumequinum HuB2013 | NC028833 Alphacoronavirus Nyctacovirus China 2013 Nyctalus velutinus bat | 81.6% |
| NC018871 Alphacoronavirus Decacovirus China 2005 Rousettus bat HKU10 | NC048216 Alphacoronavirus Setracovirus Kenya 2010 Triaenops afer bat NL63 related bat coronavirus BtKYNL63 9b | 81.6% |
| **NC009657 Alphacoronavirus Pedacovirus China 2005 Scotophilus bat** | **OP700657 Tadarida brasilensis bat alphacoronavirus 2 isolate Tb3** | **81.6%** |
| **NC009657 Alphacoronavirus Pedacovirus China 2005 Scotophilus bat** | **OP715780 Tadarida brasilensis bat alphacoronavirus 2 isolate Tb2** | **81.6%** |
| MZ081397 Alphacoronavirus China 2020 Myotis laniger | NC018871 Alphacoronavirus Decacovirus China 2005 Rousettus bat HKU10 | 81.6% |
| MZ081397 Alphacoronavirus China 2020 Myotis laniger | NC048216 Alphacoronavirus Setracovirus Kenya 2010 Triaenops afer bat NL63 related bat coronavirus BtKYNL63 9b | 81.6% |
| MZ328299 Alphacoronavirus China 2016 Miniopterus schreibersii | NC018871 Alphacoronavirus Decacovirus China 2005 Rousettus bat HKU10 | 81.5% |
| NC010437 Alphacoronavirus Minunacovirus Hong Kong 2004 Miniopterus magnater | NC018871 Alphacoronavirus Decacovirus China 2005 Rousettus bat HKU10 | 81.5% |
| MN535734 Coronavirinae Denmark 2016 Myotis dasycneme | NC028814 Alphacoronavirus Decacovirus China 2013 Rhinolophus ferrumequinum HuB2013 | 81.5% |
| NC010437 Alphacoronavirus Minunacovirus Hong Kong 2004 Miniopterus magnater | NC046964 Alphacoronavirus Nyctacovirus Italy 2015 Pipistrellus kuhlii | 81.5% |
| NC018871 Alphacoronavirus Decacovirus China 2005 Rousettus bat HKU10 | NC028833 Alphacoronavirus Nyctacovirus China 2013 Nyctalus velutinus bat | 81.4% |
| NC002645 Alphacoronavirus Duvinacovirus Human coronavirus 229E | NC005831 Alphacoronavirus Setracovirus Netherlands 2002 Human NL63 | 81.4% |
| MZ328299 Alphacoronavirus China 2016 Miniopterus schreibersii | NC048216 Alphacoronavirus Setracovirus Kenya 2010 Triaenops afer bat NL63 related bat coronavirus BtKYNL63 9b | 81.3% |
| NC028811 Alphacoronavirus Myotacovirus China 2011 Myotis ricketti SAX2011 | NC048216 Alphacoronavirus Setracovirus Kenya 2010 Triaenops afer bat NL63 related bat coronavirus BtKYNL63 9b | 81.3% |
| **NC032107 Alphacoronavirus Setracovirus Kenia 2010 Triaenops afer NL63 related bat coronavirus BtKYNL63 9a** | **OP715780 Tadarida brasilensis bat alphacoronavirus 2 isolate Tb2** | **81.3%** |
| MZ328298 Alphacoronavirus China 2016 Myotis chinensis | NC018871 Alphacoronavirus Decacovirus China 2005 Rousettus bat HKU10 | 81.3% |
| **NC032107 Alphacoronavirus Setracovirus Kenia 2010 Triaenops afer NL63 related bat coronavirus BtKYNL63 9a** | **OP700657 Tadarida brasilensis bat alphacoronavirus 2 isolate Tb3** | **81.3%** |
| **NC028833 Alphacoronavirus Nyctacovirus China 2013 Nyctalus velutinus bat** | **OP715780 Tadarida brasilensis bat alphacoronavirus 2 isolate Tb2** | **81.3%** |
| NC005831 Alphacoronavirus Setracovirus Netherlands 2002 Human NL63 | NC028814 Alphacoronavirus Decacovirus China 2013 Rhinolophus ferrumequinum HuB2013 | 81.2% |
| **NC028833 Alphacoronavirus Nyctacovirus China 2013 Nyctalus velutinus bat** | **OP700657 Tadarida brasilensis bat alphacoronavirus 2 isolate Tb3** | **81.2%** |
| NC018871 Alphacoronavirus Decacovirus China 2005 Rousettus bat HKU10 | NC032107 Alphacoronavirus Setracovirus Kenia 2010 Triaenops afer NL63 related bat coronavirus BtKYNL63 9a | 81.1% |
| NC010438 Alphacoronavirus Minunacovirus Hong Kong 2004 Miniopterus bat HKU8 | NC048216 Alphacoronavirus Setracovirus Kenya 2010 Triaenops afer bat NL63 related bat coronavirus BtKYNL63 9b | 81.1% |
| NC010438 Alphacoronavirus Minunacovirus Hong Kong 2004 Miniopterus bat HKU8 | NC046964 Alphacoronavirus Nyctacovirus Italy 2015 Pipistrellus kuhlii | 81.1% |
| NC018871 Alphacoronavirus Decacovirus China 2005 Rousettus bat HKU10 | NC028811 Alphacoronavirus Myotacovirus China 2011 Myotis ricketti SAX2011 | 81.1% |
| MZ328298 Alphacoronavirus China 2016 Myotis chinensis | NC048216 Alphacoronavirus Setracovirus Kenya 2010 Triaenops afer bat NL63 related bat coronavirus BtKYNL63 9b | 81.1% |
| NC009657 Alphacoronavirus Pedacovirus China 2005 Scotophilus bat | NC022103 Alphacoronavirus Colacovirus U.S. 2006 Myotis lucifugus | 81.0% |
| NC010437 Alphacoronavirus Minunacovirus Hong Kong 2004 Miniopterus magnater | NC028833 Alphacoronavirus Nyctacovirus China 2013 Nyctalus velutinus bat | 81.0% |
| NC010437 Alphacoronavirus Minunacovirus Hong Kong 2004 Miniopterus magnater | NC048216 Alphacoronavirus Setracovirus Kenya 2010 Triaenops afer bat NL63 related bat coronavirus BtKYNL63 9b | 80.9% |
| MN535734 Coronavirinae Denmark 2016 Myotis dasycneme | NC048216 Alphacoronavirus Setracovirus Kenya 2010 Triaenops afer bat NL63 related bat coronavirus BtKYNL63 9b | 80.9% |
| **NC010437 Alphacoronavirus Minunacovirus Hong Kong 2004 Miniopterus magnater** | **OP715780 Tadarida brasilensis bat alphacoronavirus 2 isolate Tb2** | **80.9%** |
| **NC010437 Alphacoronavirus Minunacovirus Hong Kong 2004 Miniopterus magnater** | **OP700657 Tadarida brasilensis bat alphacoronavirus 2 isolate Tb3** | **80.8%** |
| MN535734 Coronavirinae Denmark 2016 Myotis dasycneme | NC018871 Alphacoronavirus Decacovirus China 2005 Rousettus bat HKU10 | 80.8% |
| MZ081397 Alphacoronavirus China 2020 Myotis laniger | NC032107 Alphacoronavirus Setracovirus Kenia 2010 Triaenops afer NL63 related bat coronavirus BtKYNL63 9a | 80.8% |
| **MZ081397 Alphacoronavirus China 2020 Myotis laniger** | **OP715780 Tadarida brasilensis bat alphacoronavirus 2 isolate Tb2** | **80.7%** |
| NC010438 Alphacoronavirus Minunacovirus Hong Kong 2004 Miniopterus bat HKU8 | NC032107 Alphacoronavirus Setracovirus Kenia 2010 Triaenops afer NL63 related bat coronavirus BtKYNL63 9a | 80.7% |
| **MZ081397 Alphacoronavirus China 2020 Myotis laniger** | **OP700657 Tadarida brasilensis bat alphacoronavirus 2 isolate Tb3** | **80.7%** |
| MZ328299 Alphacoronavirus China 2016 Miniopterus schreibersii | NC032107 Alphacoronavirus Setracovirus Kenia 2010 Triaenops afer NL63 related bat coronavirus BtKYNL63 9a | 80.6% |
| **MZ328298 Alphacoronavirus China 2016 Myotis chinensis** | **OP715780 Tadarida brasilensis bat alphacoronavirus 2 isolate Tb2** | **80.6%** |
| **MZ328298 Alphacoronavirus China 2016 Myotis chinensis** | **OP700657 Tadarida brasilensis bat alphacoronavirus 2 isolate Tb3** | **80.5%** |
| **NC028811 Alphacoronavirus Myotacovirus China 2011 Myotis ricketti SAX2011** | **OP715780 Tadarida brasilensis bat alphacoronavirus 2 isolate Tb2** | **80.5%** |
| **NC028811 Alphacoronavirus Myotacovirus China 2011 Myotis ricketti SAX2011** | **OP700657 Tadarida brasilensis bat alphacoronavirus 2 isolate Tb3** | **80.4%** |
| **MZ328299 Alphacoronavirus China 2016 Miniopterus schreibersii** | **OP715780 Tadarida brasilensis bat alphacoronavirus 2 isolate Tb2** | **80.4%** |
| NC003436 Alphacoronavirus Pedacovirus Porcine | NC048216 Alphacoronavirus Setracovirus Kenya 2010 Triaenops afer bat NL63 related bat coronavirus BtKYNL63 9b | 80.4% |
| **MZ328299 Alphacoronavirus China 2016 Miniopterus schreibersii** | **OP700657 Tadarida brasilensis bat alphacoronavirus 2 isolate Tb3** | **80.4%** |
| NC003436 Alphacoronavirus Pedacovirus Porcine | NC010438 Alphacoronavirus Minunacovirus Hong Kong 2004 Miniopterus bat HKU8 | 80.3% |
| **NC005831 Alphacoronavirus Setracovirus Netherlands 2002 Human NL63** | **OP715780 Tadarida brasilensis bat alphacoronavirus 2 isolate Tb2** | **80.3%** |
| NC046964 Alphacoronavirus Nyctacovirus Italy 2015 Pipistrellus kuhlii | NC048216 Alphacoronavirus Setracovirus Kenya 2010 Triaenops afer bat NL63 related bat coronavirus BtKYNL63 9b | 80.3% |
| NC005831 Alphacoronavirus Setracovirus Netherlands 2002 Human NL63 | NC010438 Alphacoronavirus Minunacovirus Hong Kong 2004 Miniopterus bat HKU8 | 80.3% |
| NC005831 Alphacoronavirus Setracovirus Netherlands 2002 Human NL63 | NC018871 Alphacoronavirus Decacovirus China 2005 Rousettus bat HKU10 | 80.3% |
| **NC005831 Alphacoronavirus Setracovirus Netherlands 2002 Human NL63** | **OP700657 Tadarida brasilensis bat alphacoronavirus 2 isolate Tb3** | **80.3%** |
| NC022103 Alphacoronavirus Colacovirus U.S. 2006 Myotis lucifugus | NC028814 Alphacoronavirus Decacovirus China 2013 Rhinolophus ferrumequinum HuB2013 | 80.2% |
| NC028811 Alphacoronavirus Myotacovirus China 2011 Myotis ricketti SAX2011 | NC032107 Alphacoronavirus Setracovirus Kenia 2010 Triaenops afer NL63 related bat coronavirus BtKYNL63 9a | 80.2% |
| **MN535734 Coronavirinae Denmark 2016 Myotis dasycneme** | **OP715780 Tadarida brasilensis bat alphacoronavirus 2 isolate Tb2** | **80.2%** |
| MZ328299 Alphacoronavirus China 2016 Miniopterus schreibersii | NC046964 Alphacoronavirus Nyctacovirus Italy 2015 Pipistrellus kuhlii | 80.2% |
| NC003436 Alphacoronavirus Pedacovirus Porcine | NC010437 Alphacoronavirus Minunacovirus Hong Kong 2004 Miniopterus magnater | 80.2% |
| NC010438 Alphacoronavirus Minunacovirus Hong Kong 2004 Miniopterus bat HKU8 | NC028833 Alphacoronavirus Nyctacovirus China 2013 Nyctalus velutinus bat | 80.2% |
| NC003436 Alphacoronavirus Pedacovirus Porcine | NC032107 Alphacoronavirus Setracovirus Kenia 2010 Triaenops afer NL63 related bat coronavirus BtKYNL63 9a | 80.2% |
| NC028833 Alphacoronavirus Nyctacovirus China 2013 Nyctalus velutinus bat | NC048216 Alphacoronavirus Setracovirus Kenya 2010 Triaenops afer bat NL63 related bat coronavirus BtKYNL63 9b | 80.2% |
| **MN535734 Coronavirinae Denmark 2016 Myotis dasycneme** | **OP700657 Tadarida brasilensis bat alphacoronavirus 2 isolate Tb3** | **80.2%** |
| NC028811 Alphacoronavirus Myotacovirus China 2011 Myotis ricketti SAX2011 | NC046964 Alphacoronavirus Nyctacovirus Italy 2015 Pipistrellus kuhlii | 80.1% |
| MZ081397 Alphacoronavirus China 2020 Myotis laniger | NC046964 Alphacoronavirus Nyctacovirus Italy 2015 Pipistrellus kuhlii | 80.1% |
| MZ328298 Alphacoronavirus China 2016 Myotis chinensis | NC046964 Alphacoronavirus Nyctacovirus Italy 2015 Pipistrellus kuhlii | 80.1% |
| MZ328298 Alphacoronavirus China 2016 Myotis chinensis | NC003436 Alphacoronavirus Pedacovirus Porcine | 80.1% |
| NC003436 Alphacoronavirus Pedacovirus Porcine | NC028811 Alphacoronavirus Myotacovirus China 2011 Myotis ricketti SAX2011 | 80.0% |
| NC010438 Alphacoronavirus Minunacovirus Hong Kong 2004 Miniopterus bat HKU8 | NC028811 Alphacoronavirus Myotacovirus China 2011 Myotis ricketti SAX2011 | 80.0% |
| MZ328298 Alphacoronavirus China 2016 Myotis chinensis | NC032107 Alphacoronavirus Setracovirus Kenia 2010 Triaenops afer NL63 related bat coronavirus BtKYNL63 9a | 80.0% |
| NC010437 Alphacoronavirus Minunacovirus Hong Kong 2004 Miniopterus magnater | NC032107 Alphacoronavirus Setracovirus Kenia 2010 Triaenops afer NL63 related bat coronavirus BtKYNL63 9a | 80.0% |
| MZ081397 Alphacoronavirus China 2020 Myotis laniger | NC003436 Alphacoronavirus Pedacovirus Porcine | 80.0% |
| MZ328298 Alphacoronavirus China 2016 Myotis chinensis | NC010438 Alphacoronavirus Minunacovirus Hong Kong 2004 Miniopterus bat HKU8 | 80.0% |
| NC003436 Alphacoronavirus Pedacovirus Porcine | NC046964 Alphacoronavirus Nyctacovirus Italy 2015 Pipistrellus kuhlii | 79.9% |
| NC009988 Alphacoronavirus Rhinacovirus China 2006 Rhinolophus bat HKU2 | NC048216 Alphacoronavirus Setracovirus Kenya 2010 Triaenops afer bat NL63 related bat coronavirus BtKYNL63 9b | 79.8% |
| MZ328299 Alphacoronavirus China 2016 Miniopterus schreibersii | NC010438 Alphacoronavirus Minunacovirus Hong Kong 2004 Miniopterus bat HKU8 | 79.8% |
| NC009657 Alphacoronavirus Pedacovirus China 2005 Scotophilus bat | NC028814 Alphacoronavirus Decacovirus China 2013 Rhinolophus ferrumequinum HuB2013 | 79.8% |
| NC028811 Alphacoronavirus Myotacovirus China 2011 Myotis ricketti SAX2011 | NC028833 Alphacoronavirus Nyctacovirus China 2013 Nyctalus velutinus bat | 79.8% |
| MN535734 Coronavirinae Denmark 2016 Myotis dasycneme | NC010438 Alphacoronavirus Minunacovirus Hong Kong 2004 Miniopterus bat HKU8 | 79.7% |
| MN535734 Coronavirinae Denmark 2016 Myotis dasycneme | NC028833 Alphacoronavirus Nyctacovirus China 2013 Nyctalus velutinus bat | 79.7% |
| MZ328298 Alphacoronavirus China 2016 Myotis chinensis | NC028833 Alphacoronavirus Nyctacovirus China 2013 Nyctalus velutinus bat | 79.7% |
| MN535734 Coronavirinae Denmark 2016 Myotis dasycneme | NC032107 Alphacoronavirus Setracovirus Kenia 2010 Triaenops afer NL63 related bat coronavirus BtKYNL63 9a | 79.7% |
| NC028824 Alphacoronavirus Rhinacovirus China 2013 Rhinolophus ferrumequinum YN2012 | NC048216 Alphacoronavirus Setracovirus Kenya 2010 Triaenops afer bat NL63 related bat coronavirus BtKYNL63 9b | 79.7% |
| MZ081397 Alphacoronavirus China 2020 Myotis laniger | NC010438 Alphacoronavirus Minunacovirus Hong Kong 2004 Miniopterus bat HKU8 | 79.7% |
| MZ081397 Alphacoronavirus China 2020 Myotis laniger | NC022103 Alphacoronavirus Colacovirus U.S. 2006 Myotis lucifugus | 79.7% |
| MN535734 Coronavirinae Denmark 2016 Myotis dasycneme | NC046964 Alphacoronavirus Nyctacovirus Italy 2015 Pipistrellus kuhlii | 79.7% |
| NC032107 Alphacoronavirus Setracovirus Kenia 2010 Triaenops afer NL63 related bat coronavirus BtKYNL63 9a | NC046964 Alphacoronavirus Nyctacovirus Italy 2015 Pipistrellus kuhlii | 79.7% |
| NC009657 Alphacoronavirus Pedacovirus China 2005 Scotophilus bat | NC018871 Alphacoronavirus Decacovirus China 2005 Rousettus bat HKU10 | 79.7% |
| NC022103 Alphacoronavirus Colacovirus U.S. 2006 Myotis lucifugus | NC028833 Alphacoronavirus Nyctacovirus China 2013 Nyctalus velutinus bat | 79.7% |
| NC028824 Alphacoronavirus Rhinacovirus China 2013 Rhinolophus ferrumequinum YN2012 | NC032107 Alphacoronavirus Setracovirus Kenia 2010 Triaenops afer NL63 related bat coronavirus BtKYNL63 9a | 79.7% |
| MZ081397 Alphacoronavirus China 2020 Myotis laniger | NC028833 Alphacoronavirus Nyctacovirus China 2013 Nyctalus velutinus bat | 79.6% |
| NC003436 Alphacoronavirus Pedacovirus Porcine | NC028833 Alphacoronavirus Nyctacovirus China 2013 Nyctalus velutinus bat | 79.6% |
| NC009657 Alphacoronavirus Pedacovirus China 2005 Scotophilus bat | NC046964 Alphacoronavirus Nyctacovirus Italy 2015 Pipistrellus kuhlii | 79.5% |
| MN535734 Coronavirinae Denmark 2016 Myotis dasycneme | NC003436 Alphacoronavirus Pedacovirus Porcine | 79.5% |
| MZ328299 Alphacoronavirus China 2016 Miniopterus schreibersii | NC010437 Alphacoronavirus Minunacovirus Hong Kong 2004 Miniopterus magnater | 79.5% |
| NC009988 Alphacoronavirus Rhinacovirus China 2006 Rhinolophus bat HKU2 | NC032107 Alphacoronavirus Setracovirus Kenia 2010 Triaenops afer NL63 related bat coronavirus BtKYNL63 9a | 79.5% |
| MZ081397 Alphacoronavirus China 2020 Myotis laniger | NC010437 Alphacoronavirus Minunacovirus Hong Kong 2004 Miniopterus magnater | 79.5% |
| MZ328299 Alphacoronavirus China 2016 Miniopterus schreibersii | NC003436 Alphacoronavirus Pedacovirus Porcine | 79.5% |
| MN535734 Coronavirinae Denmark 2016 Myotis dasycneme | NC022103 Alphacoronavirus Colacovirus U.S. 2006 Myotis lucifugus | 79.5% |
| NC018871 Alphacoronavirus Decacovirus China 2005 Rousettus bat HKU10 | NC022103 Alphacoronavirus Colacovirus U.S. 2006 Myotis lucifugus | 79.5% |
| NC010438 Alphacoronavirus Minunacovirus Hong Kong 2004 Miniopterus bat HKU8 | NC028752 Alphacoronavirus Duvinacovirus Saudi Arabia 2015 Camel | 79.5% |
| MN535734 Coronavirinae Denmark 2016 Myotis dasycneme | NC010437 Alphacoronavirus Minunacovirus Hong Kong 2004 Miniopterus magnater | 79.4% |
| MZ328299 Alphacoronavirus China 2016 Miniopterus schreibersii | NC022103 Alphacoronavirus Colacovirus U.S. 2006 Myotis lucifugus | 79.4% |
| NC010437 Alphacoronavirus Minunacovirus Hong Kong 2004 Miniopterus magnater | NC028811 Alphacoronavirus Myotacovirus China 2011 Myotis ricketti SAX2011 | 79.4% |
| MZ328298 Alphacoronavirus China 2016 Myotis chinensis | NC010437 Alphacoronavirus Minunacovirus Hong Kong 2004 Miniopterus magnater | 79.4% |
| MZ328298 Alphacoronavirus China 2016 Myotis chinensis | NC022103 Alphacoronavirus Colacovirus U.S. 2006 Myotis lucifugus | 79.4% |
| NC028752 Alphacoronavirus Duvinacovirus Saudi Arabia 2015 Camel | NC028814 Alphacoronavirus Decacovirus China 2013 Rhinolophus ferrumequinum HuB2013 | 79.4% |
| NC005831 Alphacoronavirus Setracovirus Netherlands 2002 Human NL63 | NC028811 Alphacoronavirus Myotacovirus China 2011 Myotis ricketti SAX2011 | 79.3% |
| MZ328299 Alphacoronavirus China 2016 Miniopterus schreibersii | NC028833 Alphacoronavirus Nyctacovirus China 2013 Nyctalus velutinus bat | 79.3% |
| NC022103 Alphacoronavirus Colacovirus U.S. 2006 Myotis lucifugus | NC048216 Alphacoronavirus Setracovirus Kenya 2010 Triaenops afer bat NL63 related bat coronavirus BtKYNL63 9b | 79.2% |
| MZ081397 Alphacoronavirus China 2020 Myotis laniger | NC005831 Alphacoronavirus Setracovirus Netherlands 2002 Human NL63 | 79.2% |
| MZ081397 Alphacoronavirus China 2020 Myotis laniger | OL410609 Alphacoronavirus U.S. 2020 Eptesicus fuscus | 79.2% |
| MZ328298 Alphacoronavirus China 2016 Myotis chinensis | NC005831 Alphacoronavirus Setracovirus Netherlands 2002 Human NL63 | 79.2% |
| NC022103 Alphacoronavirus Colacovirus U.S. 2006 Myotis lucifugus | NC028811 Alphacoronavirus Myotacovirus China 2011 Myotis ricketti SAX2011 | 79.2% |
| MZ081397 Alphacoronavirus China 2020 Myotis laniger | NC009988 Alphacoronavirus Rhinacovirus China 2006 Rhinolophus bat HKU2 | 79.2% |
| MZ081397 Alphacoronavirus China 2020 Myotis laniger | NC028752 Alphacoronavirus Duvinacovirus Saudi Arabia 2015 Camel | 79.2% |
| MZ081397 Alphacoronavirus China 2020 Myotis laniger | OL415262 Alphacoronavirus U.S. 2021 Eptesicus fuscus | 79.2% |
| NC018871 Alphacoronavirus Decacovirus China 2005 Rousettus bat HKU10 | NC028752 Alphacoronavirus Duvinacovirus Saudi Arabia 2015 Camel | 79.1% |
| NC028833 Alphacoronavirus Nyctacovirus China 2013 Nyctalus velutinus bat | NC032107 Alphacoronavirus Setracovirus Kenia 2010 Triaenops afer NL63 related bat coronavirus BtKYNL63 9a | 79.1% |
| NC028811 Alphacoronavirus Myotacovirus China 2011 Myotis ricketti SAX2011 | OL410609 Alphacoronavirus U.S. 2020 Eptesicus fuscus | 79.1% |
| MN535734 Coronavirinae Denmark 2016 Myotis dasycneme | OL415262 Alphacoronavirus U.S. 2021 Eptesicus fuscus | 79.1% |
| NC048216 Alphacoronavirus Setracovirus Kenya 2010 Triaenops afer bat NL63 related bat coronavirus BtKYNL63 9b | MZ293744 Alphacoronavirus Brazil 2014 Gardnerycteris crenulatum bat | 79.1% |
| MZ081397 Alphacoronavirus China 2020 Myotis laniger | OL410607 Alphacoronavirus U.S. 2020 Eptesicus fuscus | 79.1% |
| MZ328299 Alphacoronavirus China 2016 Miniopterus schreibersii | NC005831 Alphacoronavirus Setracovirus Netherlands 2002 Human NL63 | 79.1% |
| MZ328299 Alphacoronavirus China 2016 Miniopterus schreibersii | NC028752 Alphacoronavirus Duvinacovirus Saudi Arabia 2015 Camel | 79.1% |
| MN535734 Coronavirinae Denmark 2016 Myotis dasycneme | OL410607 Alphacoronavirus U.S. 2020 Eptesicus fuscus | 79.1% |
| MN535734 Coronavirinae Denmark 2016 Myotis dasycneme | OL410609 Alphacoronavirus U.S. 2020 Eptesicus fuscus | 79.1% |
| MZ328298 Alphacoronavirus China 2016 Myotis chinensis | OL410609 Alphacoronavirus U.S. 2020 Eptesicus fuscus | 79.1% |
| NC028811 Alphacoronavirus Myotacovirus China 2011 Myotis ricketti SAX2011 | OL415262 Alphacoronavirus U.S. 2021 Eptesicus fuscus | 79.1% |
| NC003436 Alphacoronavirus Pedacovirus Porcine | NC005831 Alphacoronavirus Setracovirus Netherlands 2002 Human NL63 | 79.0% |
| MN535734 Coronavirinae Denmark 2016 Myotis dasycneme | NC009657 Alphacoronavirus Pedacovirus China 2005 Scotophilus bat | 79.0% |
| NC028811 Alphacoronavirus Myotacovirus China 2011 Myotis ricketti SAX2011 | OL410607 Alphacoronavirus U.S. 2020 Eptesicus fuscus | 79.0% |
| MZ328298 Alphacoronavirus China 2016 Myotis chinensis | OL415262 Alphacoronavirus U.S. 2021 Eptesicus fuscus | 79.0% |
| MZ081397 Alphacoronavirus China 2020 Myotis laniger | NC028824 Alphacoronavirus Rhinacovirus China 2013 Rhinolophus ferrumequinum YN2012 | 79.0% |
| **MZ081397 Alphacoronavirus China 2020 Myotis laniger** | **OP715781 Tadarida brasiliensis bat alphacoronavirus 1 isolate Tb1** | **79.0%** |
| MZ328299 Alphacoronavirus China 2016 Miniopterus schreibersii | NC009988 Alphacoronavirus Rhinacovirus China 2006 Rhinolophus bat HKU2 | 79.0% |
| NC002645 Alphacoronavirus Duvinacovirus Human coronavirus 229E | NC010438 Alphacoronavirus Minunacovirus Hong Kong 2004 Miniopterus bat HKU8 | 79.0% |
| NC028752 Alphacoronavirus Duvinacovirus Saudi Arabia 2015 Camel | NC028811 Alphacoronavirus Myotacovirus China 2011 Myotis ricketti SAX2011 | 79.0% |
| MZ328298 Alphacoronavirus China 2016 Myotis chinensis | OL410607 Alphacoronavirus U.S. 2020 Eptesicus fuscus | 79.0% |
| NC022103 Alphacoronavirus Colacovirus U.S. 2006 Myotis lucifugus | NC046964 Alphacoronavirus Nyctacovirus Italy 2015 Pipistrellus kuhlii | 78.9% |
| NC005831 Alphacoronavirus Setracovirus Netherlands 2002 Human NL63 | NC010437 Alphacoronavirus Minunacovirus Hong Kong 2004 Miniopterus magnater | 78.9% |
| NC002645 Alphacoronavirus Duvinacovirus Human coronavirus 229E | NC028814 Alphacoronavirus Decacovirus China 2013 Rhinolophus ferrumequinum HuB2013 | 78.9% |
| MT663548 Alphacoronavirus Peru 2015 Desmodus rotundus | NC048216 Alphacoronavirus Setracovirus Kenya 2010 Triaenops afer bat NL63 related bat coronavirus BtKYNL63 9b | 78.9% |
| NC009657 Alphacoronavirus Pedacovirus China 2005 Scotophilus bat | NC028811 Alphacoronavirus Myotacovirus China 2011 Myotis ricketti SAX2011 | 78.9% |
| MW249018 Coronavirinae Peru 2016 Desmodus rotundus | NC048216 Alphacoronavirus Setracovirus Kenya 2010 Triaenops afer bat NL63 related bat coronavirus BtKYNL63 9b | 78.9% |
| **MN535734 Coronavirinae Denmark 2016 Myotis dasycneme** | **OP715781 Tadarida brasiliensis bat alphacoronavirus 1 isolate Tb1** | **78.9%** |
| MZ081397 Alphacoronavirus China 2020 Myotis laniger | NC009657 Alphacoronavirus Pedacovirus China 2005 Scotophilus bat | 78.8% |
| MN535734 Coronavirinae Denmark 2016 Myotis dasycneme | MW924112 Alphacoronavirus Korea 2020 Eptesicus serotinus HCQD2020 | 78.8% |
| MW924112 Alphacoronavirus Korea 2020 Eptesicus serotinus HCQD2020 | NC028811 Alphacoronavirus Myotacovirus China 2011 Myotis ricketti SAX2011 | 78.8% |
| MW924112 Alphacoronavirus Korea 2020 Eptesicus serotinus HCQD2020 | MZ081397 Alphacoronavirus China 2020 Myotis laniger | 78.8% |
| MZ328298 Alphacoronavirus China 2016 Myotis chinensis | NC028752 Alphacoronavirus Duvinacovirus Saudi Arabia 2015 Camel | 78.8% |
| NC010437 Alphacoronavirus Minunacovirus Hong Kong 2004 Miniopterus magnater | NC028752 Alphacoronavirus Duvinacovirus Saudi Arabia 2015 Camel | 78.8% |
| NC010437 Alphacoronavirus Minunacovirus Hong Kong 2004 Miniopterus magnater | NC028824 Alphacoronavirus Rhinacovirus China 2013 Rhinolophus ferrumequinum YN2012 | 78.8% |
| MZ081397 Alphacoronavirus China 2020 Myotis laniger | NC002645 Alphacoronavirus Duvinacovirus Human coronavirus 229E | 78.8% |
| MZ328298 Alphacoronavirus China 2016 Myotis chinensis | NC009657 Alphacoronavirus Pedacovirus China 2005 Scotophilus bat | 78.8% |
| MZ328299 Alphacoronavirus China 2016 Miniopterus schreibersii | NC009657 Alphacoronavirus Pedacovirus China 2005 Scotophilus bat | 78.8% |
| NC009988 Alphacoronavirus Rhinacovirus China 2006 Rhinolophus bat HKU2 | NC010437 Alphacoronavirus Minunacovirus Hong Kong 2004 Miniopterus magnater | 78.8% |
| MZ328299 Alphacoronavirus China 2016 Miniopterus schreibersii | NC028824 Alphacoronavirus Rhinacovirus China 2013 Rhinolophus ferrumequinum YN2012 | 78.8% |
| NC022103 Alphacoronavirus Colacovirus U.S. 2006 Myotis lucifugus | NC032107 Alphacoronavirus Setracovirus Kenia 2010 Triaenops afer NL63 related bat coronavirus BtKYNL63 9a | 78.8% |
| MZ328299 Alphacoronavirus China 2016 Miniopterus schreibersii | OL410609 Alphacoronavirus U.S. 2020 Eptesicus fuscus | 78.8% |
| MW924112 Alphacoronavirus Korea 2020 Eptesicus serotinus HCQD2020 | MZ328298 Alphacoronavirus China 2016 Myotis chinensis | 78.7% |
| MN535734 Coronavirinae Denmark 2016 Myotis dasycneme | NC005831 Alphacoronavirus Setracovirus Netherlands 2002 Human NL63 | 78.7% |
| NC010438 Alphacoronavirus Minunacovirus Hong Kong 2004 Miniopterus bat HKU8 | NC022103 Alphacoronavirus Colacovirus U.S. 2006 Myotis lucifugus | 78.7% |
| NC009657 Alphacoronavirus Pedacovirus China 2005 Scotophilus bat | NC048216 Alphacoronavirus Setracovirus Kenya 2010 Triaenops afer bat NL63 related bat coronavirus BtKYNL63 9b | 78.7% |
| MZ328299 Alphacoronavirus China 2016 Miniopterus schreibersii | OL415262 Alphacoronavirus U.S. 2021 Eptesicus fuscus | 78.7% |
| MZ328299 Alphacoronavirus China 2016 Miniopterus schreibersii | NC002645 Alphacoronavirus Duvinacovirus Human coronavirus 229E | 78.6% |
| MZ328299 Alphacoronavirus China 2016 Miniopterus schreibersii | OL410607 Alphacoronavirus U.S. 2020 Eptesicus fuscus | 78.6% |
| NC002645 Alphacoronavirus Duvinacovirus Human coronavirus 229E | NC018871 Alphacoronavirus Decacovirus China 2005 Rousettus bat HKU10 | 78.6% |
| NC009988 Alphacoronavirus Rhinacovirus China 2006 Rhinolophus bat HKU2 | NC028752 Alphacoronavirus Duvinacovirus Saudi Arabia 2015 Camel | 78.6% |
| **NC028811 Alphacoronavirus Myotacovirus China 2011 Myotis ricketti SAX2011** | **OP715781 Tadarida brasiliensis bat alphacoronavirus 1 isolate Tb1** | **78.6%** |
| NC005831 Alphacoronavirus Setracovirus Netherlands 2002 Human NL63 | NC046964 Alphacoronavirus Nyctacovirus Italy 2015 Pipistrellus kuhlii | 78.5% |
| **MZ328298 Alphacoronavirus China 2016 Myotis chinensis** | **OP715781 Tadarida brasiliensis bat alphacoronavirus 1 isolate Tb1** | **78.5%** |
| **MZ328299 Alphacoronavirus China 2016 Miniopterus schreibersii** | **OP715781 Tadarida brasiliensis bat alphacoronavirus 1 isolate Tb1** | **78.5%** |
| NC009657 Alphacoronavirus Pedacovirus China 2005 Scotophilus bat | NC028833 Alphacoronavirus Nyctacovirus China 2013 Nyctalus velutinus bat | 78.5% |
| **NC048216 Alphacoronavirus Setracovirus Kenya 2010 Triaenops afer bat NL63 related bat coronavirus BtKYNL63 9b** | **OP715781 Tadarida brasiliensis bat alphacoronavirus 1 isolate Tb1** | **78.5%** |
| NC002645 Alphacoronavirus Duvinacovirus Human coronavirus 229E | NC028811 Alphacoronavirus Myotacovirus China 2011 Myotis ricketti SAX2011 | 78.4% |
| MW924112 Alphacoronavirus Korea 2020 Eptesicus serotinus HCQD2020 | MZ328299 Alphacoronavirus China 2016 Miniopterus schreibersii | 78.4% |
| NC002645 Alphacoronavirus Duvinacovirus Human coronavirus 229E | NC009988 Alphacoronavirus Rhinacovirus China 2006 Rhinolophus bat HKU2 | 78.4% |
| NC010437 Alphacoronavirus Minunacovirus Hong Kong 2004 Miniopterus magnater | NC022103 Alphacoronavirus Colacovirus U.S. 2006 Myotis lucifugus | 78.4% |
| MN535734 Coronavirinae Denmark 2016 Myotis dasycneme | NC028752 Alphacoronavirus Duvinacovirus Saudi Arabia 2015 Camel | 78.4% |
| NC028814 Alphacoronavirus Decacovirus China 2013 Rhinolophus ferrumequinum HuB2013 | NC028824 Alphacoronavirus Rhinacovirus China 2013 Rhinolophus ferrumequinum YN2012 | 78.4% |
| **NC028752 Alphacoronavirus Duvinacovirus Saudi Arabia 2015 Camel** | **OP715780 Tadarida brasilensis bat alphacoronavirus 2 isolate Tb2** | **78.4%** |
| NC002645 Alphacoronavirus Duvinacovirus Human coronavirus 229E | NC010437 Alphacoronavirus Minunacovirus Hong Kong 2004 Miniopterus magnater | 78.3% |
| NC009988 Alphacoronavirus Rhinacovirus China 2006 Rhinolophus bat HKU2 | NC028814 Alphacoronavirus Decacovirus China 2013 Rhinolophus ferrumequinum HuB2013 | 78.3% |
| NC028752 Alphacoronavirus Duvinacovirus Saudi Arabia 2015 Camel | NC028824 Alphacoronavirus Rhinacovirus China 2013 Rhinolophus ferrumequinum YN2012 | 78.3% |
| MZ328298 Alphacoronavirus China 2016 Myotis chinensis | NC002645 Alphacoronavirus Duvinacovirus Human coronavirus 229E | 78.3% |
| NC009657 Alphacoronavirus Pedacovirus China 2005 Scotophilus bat | NC010437 Alphacoronavirus Minunacovirus Hong Kong 2004 Miniopterus magnater | 78.3% |
| **NC028752 Alphacoronavirus Duvinacovirus Saudi Arabia 2015 Camel** | **OP700657 Tadarida brasilensis bat alphacoronavirus 2 isolate Tb3** | **78.3%** |
| MZ328298 Alphacoronavirus China 2016 Myotis chinensis | NC009988 Alphacoronavirus Rhinacovirus China 2006 Rhinolophus bat HKU2 | 78.2% |
| NC009988 Alphacoronavirus Rhinacovirus China 2006 Rhinolophus bat HKU2 | NC028811 Alphacoronavirus Myotacovirus China 2011 Myotis ricketti SAX2011 | 78.2% |
| NC005831 Alphacoronavirus Setracovirus Netherlands 2002 Human NL63 | NC028833 Alphacoronavirus Nyctacovirus China 2013 Nyctalus velutinus bat | 78.2% |
| NC018871 Alphacoronavirus Decacovirus China 2005 Rousettus bat HKU10 | MZ293744 Alphacoronavirus Brazil 2014 Gardnerycteris crenulatum bat | 78.2% |
| NC009657 Alphacoronavirus Pedacovirus China 2005 Scotophilus bat | NC010438 Alphacoronavirus Minunacovirus Hong Kong 2004 Miniopterus bat HKU8 | 78.1% |
| MN535734 Coronavirinae Denmark 2016 Myotis dasycneme | NC028824 Alphacoronavirus Rhinacovirus China 2013 Rhinolophus ferrumequinum YN2012 | 78.1% |
| MZ328298 Alphacoronavirus China 2016 Myotis chinensis | NC028824 Alphacoronavirus Rhinacovirus China 2013 Rhinolophus ferrumequinum YN2012 | 78.1% |
| NC028811 Alphacoronavirus Myotacovirus China 2011 Myotis ricketti SAX2011 | NC028824 Alphacoronavirus Rhinacovirus China 2013 Rhinolophus ferrumequinum YN2012 | 78.1% |
| NC028814 Alphacoronavirus Decacovirus China 2013 Rhinolophus ferrumequinum HuB2013 | MZ293744 Alphacoronavirus Brazil 2014 Gardnerycteris crenulatum bat | 78.1% |
| MN535734 Coronavirinae Denmark 2016 Myotis dasycneme | NC009988 Alphacoronavirus Rhinacovirus China 2006 Rhinolophus bat HKU2 | 78.1% |
| NC002645 Alphacoronavirus Duvinacovirus Human coronavirus 229E | NC028824 Alphacoronavirus Rhinacovirus China 2013 Rhinolophus ferrumequinum YN2012 | 78.1% |
| NC048216 Alphacoronavirus Setracovirus Kenya 2010 Triaenops afer bat NL63 related bat coronavirus BtKYNL63 9b | OL410609 Alphacoronavirus U.S. 2020 Eptesicus fuscus | 78.0% |
| NC048216 Alphacoronavirus Setracovirus Kenya 2010 Triaenops afer bat NL63 related bat coronavirus BtKYNL63 9b | OL415262 Alphacoronavirus U.S. 2021 Eptesicus fuscus | 78.0% |
| NC048216 Alphacoronavirus Setracovirus Kenya 2010 Triaenops afer bat NL63 related bat coronavirus BtKYNL63 9b | OL410607 Alphacoronavirus U.S. 2020 Eptesicus fuscus | 78.0% |
| **NC002645 Alphacoronavirus Duvinacovirus Human coronavirus 229E** | **OP715780 Tadarida brasilensis bat alphacoronavirus 2 isolate Tb2** | **78.0%** |
| MZ081397 Alphacoronavirus China 2020 Myotis laniger | MZ293744 Alphacoronavirus Brazil 2014 Gardnerycteris crenulatum bat | 78.0% |
| MW249018 Coronavirinae Peru 2016 Desmodus rotundus | NC018871 Alphacoronavirus Decacovirus China 2005 Rousettus bat HKU10 | 77.9% |
| MN535734 Coronavirinae Denmark 2016 Myotis dasycneme | NC002645 Alphacoronavirus Duvinacovirus Human coronavirus 229E | 77.9% |
| MT663548 Alphacoronavirus Peru 2015 Desmodus rotundus | NC018871 Alphacoronavirus Decacovirus China 2005 Rousettus bat HKU10 | 77.9% |
| MT663548 Alphacoronavirus Peru 2015 Desmodus rotundus | NC028814 Alphacoronavirus Decacovirus China 2013 Rhinolophus ferrumequinum HuB2013 | 77.9% |
| MW249018 Coronavirinae Peru 2016 Desmodus rotundus | NC028814 Alphacoronavirus Decacovirus China 2013 Rhinolophus ferrumequinum HuB2013 | 77.9% |
| **NC002645 Alphacoronavirus Duvinacovirus Human coronavirus 229E** | **OP700657 Tadarida brasilensis bat alphacoronavirus 2 isolate Tb3** | **77.9%** |
| NC009988 Alphacoronavirus Rhinacovirus China 2006 Rhinolophus bat HKU2 | NC010438 Alphacoronavirus Minunacovirus Hong Kong 2004 Miniopterus bat HKU8 | 77.8% |
| NC005831 Alphacoronavirus Setracovirus Netherlands 2002 Human NL63 | NC022103 Alphacoronavirus Colacovirus U.S. 2006 Myotis lucifugus | 77.8% |
| MW924112 Alphacoronavirus Korea 2020 Eptesicus serotinus HCQD2020 | NC048216 Alphacoronavirus Setracovirus Kenya 2010 Triaenops afer bat NL63 related bat coronavirus BtKYNL63 9b | 77.7% |
| NC010438 Alphacoronavirus Minunacovirus Hong Kong 2004 Miniopterus bat HKU8 | NC028824 Alphacoronavirus Rhinacovirus China 2013 Rhinolophus ferrumequinum YN2012 | 77.7% |
| NC010438 Alphacoronavirus Minunacovirus Hong Kong 2004 Miniopterus bat HKU8 | MZ293744 Alphacoronavirus Brazil 2014 Gardnerycteris crenulatum bat | 77.7% |
| NC032107 Alphacoronavirus Setracovirus Kenia 2010 Triaenops afer NL63 related bat coronavirus BtKYNL63 9a | MZ293744 Alphacoronavirus Brazil 2014 Gardnerycteris crenulatum bat | 77.7% |
| MT663548 Alphacoronavirus Peru 2015 Desmodus rotundus | MZ081397 Alphacoronavirus China 2020 Myotis laniger | 77.6% |
| NC028752 Alphacoronavirus Duvinacovirus Saudi Arabia 2015 Camel | NC046964 Alphacoronavirus Nyctacovirus Italy 2015 Pipistrellus kuhlii | 77.6% |
| MT663548 Alphacoronavirus Peru 2015 Desmodus rotundus | NC032107 Alphacoronavirus Setracovirus Kenia 2010 Triaenops afer NL63 related bat coronavirus BtKYNL63 9a | 77.6% |
| MW249018 Coronavirinae Peru 2016 Desmodus rotundus | MZ081397 Alphacoronavirus China 2020 Myotis laniger | 77.6% |
| NC046964 Alphacoronavirus Nyctacovirus Italy 2015 Pipistrellus kuhlii | MZ293744 Alphacoronavirus Brazil 2014 Gardnerycteris crenulatum bat | 77.6% |
| NC005831 Alphacoronavirus Setracovirus Netherlands 2002 Human NL63 | NC009988 Alphacoronavirus Rhinacovirus China 2006 Rhinolophus bat HKU2 | 77.5% |
| MW249018 Coronavirinae Peru 2016 Desmodus rotundus | NC032107 Alphacoronavirus Setracovirus Kenia 2010 Triaenops afer NL63 related bat coronavirus BtKYNL63 9a | 77.5% |
| MZ328299 Alphacoronavirus China 2016 Miniopterus schreibersii | MZ293744 Alphacoronavirus Brazil 2014 Gardnerycteris crenulatum bat | 77.5% |
| **OP715780 Tadarida brasilensis bat alphacoronavirus 2 isolate Tb2** | **MZ293744 Alphacoronavirus Brazil 2014 Gardnerycteris crenulatum bat** | **77.5%** |
| NC003436 Alphacoronavirus Pedacovirus Porcine | NC028752 Alphacoronavirus Duvinacovirus Saudi Arabia 2015 Camel | 77.4% |
| NC005831 Alphacoronavirus Setracovirus Netherlands 2002 Human NL63 | NC028824 Alphacoronavirus Rhinacovirus China 2013 Rhinolophus ferrumequinum YN2012 | 77.4% |
| NC028752 Alphacoronavirus Duvinacovirus Saudi Arabia 2015 Camel | NC028833 Alphacoronavirus Nyctacovirus China 2013 Nyctalus velutinus bat | 77.4% |
| NC009657 Alphacoronavirus Pedacovirus China 2005 Scotophilus bat | NC032107 Alphacoronavirus Setracovirus Kenia 2010 Triaenops afer NL63 related bat coronavirus BtKYNL63 9a | 77.4% |
| **NC010437 Alphacoronavirus Minunacovirus Hong Kong 2004 Miniopterus magnater** | **OP715781 Tadarida brasiliensis bat alphacoronavirus 1 isolate Tb1** | **77.4%** |
| NC028833 Alphacoronavirus Nyctacovirus China 2013 Nyctalus velutinus bat | MZ293744 Alphacoronavirus Brazil 2014 Gardnerycteris crenulatum bat | 77.4% |
| **OP700657 Tadarida brasilensis bat alphacoronavirus 2 isolate Tb3** | **MZ293744 Alphacoronavirus Brazil 2014 Gardnerycteris crenulatum bat** | **77.4%** |
| NC002645 Alphacoronavirus Duvinacovirus Human coronavirus 229E | NC003436 Alphacoronavirus Pedacovirus Porcine | 77.3% |
| NC028811 Alphacoronavirus Myotacovirus China 2011 Myotis ricketti SAX2011 | MZ293744 Alphacoronavirus Brazil 2014 Gardnerycteris crenulatum bat | 77.3% |
| NC010437 Alphacoronavirus Minunacovirus Hong Kong 2004 Miniopterus magnater | OL410607 Alphacoronavirus U.S. 2020 Eptesicus fuscus | 77.3% |
| NC018871 Alphacoronavirus Decacovirus China 2005 Rousettus bat HKU10 | OL410607 Alphacoronavirus U.S. 2020 Eptesicus fuscus | 77.3% |
| **NC032107 Alphacoronavirus Setracovirus Kenia 2010 Triaenops afer NL63 related bat coronavirus BtKYNL63 9a** | **OP715781 Tadarida brasiliensis bat alphacoronavirus 1 isolate Tb1** | **77.3%** |
| NC010437 Alphacoronavirus Minunacovirus Hong Kong 2004 Miniopterus magnater | MZ293744 Alphacoronavirus Brazil 2014 Gardnerycteris crenulatum bat | 77.3% |
| MT663548 Alphacoronavirus Peru 2015 Desmodus rotundus | NC010438 Alphacoronavirus Minunacovirus Hong Kong 2004 Miniopterus bat HKU8 | 77.2% |
| MW249018 Coronavirinae Peru 2016 Desmodus rotundus | NC010438 Alphacoronavirus Minunacovirus Hong Kong 2004 Miniopterus bat HKU8 | 77.2% |
| MT663548 Alphacoronavirus Peru 2015 Desmodus rotundus | NC028833 Alphacoronavirus Nyctacovirus China 2013 Nyctalus velutinus bat | 77.2% |
| NC010437 Alphacoronavirus Minunacovirus Hong Kong 2004 Miniopterus magnater | OL410609 Alphacoronavirus U.S. 2020 Eptesicus fuscus | 77.2% |
| NC018871 Alphacoronavirus Decacovirus China 2005 Rousettus bat HKU10 | OL410609 Alphacoronavirus U.S. 2020 Eptesicus fuscus | 77.2% |
| NC018871 Alphacoronavirus Decacovirus China 2005 Rousettus bat HKU10 | OL415262 Alphacoronavirus U.S. 2021 Eptesicus fuscus | 77.2% |
| **NC009988 Alphacoronavirus Rhinacovirus China 2006 Rhinolophus bat HKU2** | **OP715780 Tadarida brasilensis bat alphacoronavirus 2 isolate Tb2** | **77.2%** |
| NC003436 Alphacoronavirus Pedacovirus Porcine | MZ293744 Alphacoronavirus Brazil 2014 Gardnerycteris crenulatum bat | 77.2% |
| NC002645 Alphacoronavirus Duvinacovirus Human coronavirus 229E | NC046964 Alphacoronavirus Nyctacovirus Italy 2015 Pipistrellus kuhlii | 77.2% |
| NC028814 Alphacoronavirus Decacovirus China 2013 Rhinolophus ferrumequinum HuB2013 | OL410607 Alphacoronavirus U.S. 2020 Eptesicus fuscus | 77.2% |
| NC028814 Alphacoronavirus Decacovirus China 2013 Rhinolophus ferrumequinum HuB2013 | OL410609 Alphacoronavirus U.S. 2020 Eptesicus fuscus | 77.2% |
| NC032107 Alphacoronavirus Setracovirus Kenia 2010 Triaenops afer NL63 related bat coronavirus BtKYNL63 9a | OL410609 Alphacoronavirus U.S. 2020 Eptesicus fuscus | 77.2% |
| NC010437 Alphacoronavirus Minunacovirus Hong Kong 2004 Miniopterus magnater | OL415262 Alphacoronavirus U.S. 2021 Eptesicus fuscus | 77.2% |
| **NC009988 Alphacoronavirus Rhinacovirus China 2006 Rhinolophus bat HKU2** | **OP700657 Tadarida brasilensis bat alphacoronavirus 2 isolate Tb3** | **77.2%** |
| **NC046964 Alphacoronavirus Nyctacovirus Italy 2015 Pipistrellus kuhlii** | **OP715781 Tadarida brasiliensis bat alphacoronavirus 1 isolate Tb1** | **77.1%** |
| MT663548 Alphacoronavirus Peru 2015 Desmodus rotundus | NC003436 Alphacoronavirus Pedacovirus Porcine | 77.1% |
| MW249018 Coronavirinae Peru 2016 Desmodus rotundus | NC003436 Alphacoronavirus Pedacovirus Porcine | 77.1% |
| MT663548 Alphacoronavirus Peru 2015 Desmodus rotundus | NC010437 Alphacoronavirus Minunacovirus Hong Kong 2004 Miniopterus magnater | 77.1% |
| MW249018 Coronavirinae Peru 2016 Desmodus rotundus | NC028833 Alphacoronavirus Nyctacovirus China 2013 Nyctalus velutinus bat | 77.1% |
| NC032107 Alphacoronavirus Setracovirus Kenia 2010 Triaenops afer NL63 related bat coronavirus BtKYNL63 9a | OL415262 Alphacoronavirus U.S. 2021 Eptesicus fuscus | 77.1% |
| MZ328298 Alphacoronavirus China 2016 Myotis chinensis | MZ293744 Alphacoronavirus Brazil 2014 Gardnerycteris crenulatum bat | 77.1% |
| NC009988 Alphacoronavirus Rhinacovirus China 2006 Rhinolophus bat HKU2 | NC046964 Alphacoronavirus Nyctacovirus Italy 2015 Pipistrellus kuhlii | 77.1% |
| NC046964 Alphacoronavirus Nyctacovirus Italy 2015 Pipistrellus kuhlii | OL415262 Alphacoronavirus U.S. 2021 Eptesicus fuscus | 77.1% |
| NC009988 Alphacoronavirus Rhinacovirus China 2006 Rhinolophus bat HKU2 | NC018871 Alphacoronavirus Decacovirus China 2005 Rousettus bat HKU10 | 77.1% |
| NC018871 Alphacoronavirus Decacovirus China 2005 Rousettus bat HKU10 | NC028824 Alphacoronavirus Rhinacovirus China 2013 Rhinolophus ferrumequinum YN2012 | 77.1% |
| NC032107 Alphacoronavirus Setracovirus Kenia 2010 Triaenops afer NL63 related bat coronavirus BtKYNL63 9a | OL410607 Alphacoronavirus U.S. 2020 Eptesicus fuscus | 77.1% |
| NC028814 Alphacoronavirus Decacovirus China 2013 Rhinolophus ferrumequinum HuB2013 | OL415262 Alphacoronavirus U.S. 2021 Eptesicus fuscus | 77.1% |
| **NC028824 Alphacoronavirus Rhinacovirus China 2013 Rhinolophus ferrumequinum YN2012** | **OP715780 Tadarida brasilensis bat alphacoronavirus 2 isolate Tb2** | **77.1%** |
| MT663548 Alphacoronavirus Peru 2015 Desmodus rotundus | NC046964 Alphacoronavirus Nyctacovirus Italy 2015 Pipistrellus kuhlii | 77.0% |
| MW249018 Coronavirinae Peru 2016 Desmodus rotundus | NC046964 Alphacoronavirus Nyctacovirus Italy 2015 Pipistrellus kuhlii | 77.0% |
| NC046964 Alphacoronavirus Nyctacovirus Italy 2015 Pipistrellus kuhlii | OL410607 Alphacoronavirus U.S. 2020 Eptesicus fuscus | 77.0% |
| NC046964 Alphacoronavirus Nyctacovirus Italy 2015 Pipistrellus kuhlii | OL410609 Alphacoronavirus U.S. 2020 Eptesicus fuscus | 77.0% |
| MT663548 Alphacoronavirus Peru 2015 Desmodus rotundus | MZ328299 Alphacoronavirus China 2016 Miniopterus schreibersii | 77.0% |
| MW249018 Coronavirinae Peru 2016 Desmodus rotundus | NC010437 Alphacoronavirus Minunacovirus Hong Kong 2004 Miniopterus magnater | 77.0% |
| NC002645 Alphacoronavirus Duvinacovirus Human coronavirus 229E | NC028833 Alphacoronavirus Nyctacovirus China 2013 Nyctalus velutinus bat | 77.0% |
| NC028833 Alphacoronavirus Nyctacovirus China 2013 Nyctalus velutinus bat | OL410609 Alphacoronavirus U.S. 2020 Eptesicus fuscus | 77.0% |
| **NC028824 Alphacoronavirus Rhinacovirus China 2013 Rhinolophus ferrumequinum YN2012** | **OP700657 Tadarida brasilensis bat alphacoronavirus 2 isolate Tb3** | **77.0%** |
| **MW249018 Coronavirinae Peru 2016 Desmodus rotundus** | **OP715780 Tadarida brasilensis bat alphacoronavirus 2 isolate Tb2** | **77.0%** |
| MW924112 Alphacoronavirus Korea 2020 Eptesicus serotinus HCQD2020 | NC010437 Alphacoronavirus Minunacovirus Hong Kong 2004 Miniopterus magnater | 76.9% |
| MW249018 Coronavirinae Peru 2016 Desmodus rotundus | MZ328299 Alphacoronavirus China 2016 Miniopterus schreibersii | 76.9% |
| MT663548 Alphacoronavirus Peru 2015 Desmodus rotundus | NC028752 Alphacoronavirus Duvinacovirus Saudi Arabia 2015 Camel | 76.9% |
| MW249018 Coronavirinae Peru 2016 Desmodus rotundus | NC028752 Alphacoronavirus Duvinacovirus Saudi Arabia 2015 Camel | 76.9% |
| NC028833 Alphacoronavirus Nyctacovirus China 2013 Nyctalus velutinus bat | OL415262 Alphacoronavirus U.S. 2021 Eptesicus fuscus | 76.9% |
| **MW249018 Coronavirinae Peru 2016 Desmodus rotundus** | **OP700657 Tadarida brasilensis bat alphacoronavirus 2 isolate Tb3** | **76.9%** |
| NC028833 Alphacoronavirus Nyctacovirus China 2013 Nyctalus velutinus bat | OL410607 Alphacoronavirus U.S. 2020 Eptesicus fuscus | 76.9% |
| **MT663548 Alphacoronavirus Peru 2015 Desmodus rotundus** | **OP715780 Tadarida brasilensis bat alphacoronavirus 2 isolate Tb2** | **76.9%** |
| **NC018871 Alphacoronavirus Decacovirus China 2005 Rousettus bat HKU10** | **OP715781 Tadarida brasiliensis bat alphacoronavirus 1 isolate Tb1** | **76.9%** |
| MN535734 Coronavirinae Denmark 2016 Myotis dasycneme | MZ293744 Alphacoronavirus Brazil 2014 Gardnerycteris crenulatum bat | 76.9% |
| NC028824 Alphacoronavirus Rhinacovirus China 2013 Rhinolophus ferrumequinum YN2012 | NC046964 Alphacoronavirus Nyctacovirus Italy 2015 Pipistrellus kuhlii | 76.8% |
| **MT663548 Alphacoronavirus Peru 2015 Desmodus rotundus** | **OP700657 Tadarida brasilensis bat alphacoronavirus 2 isolate Tb3** | **76.8%** |
| **NC028814 Alphacoronavirus Decacovirus China 2013 Rhinolophus ferrumequinum HuB2013** | **OP715781 Tadarida brasiliensis bat alphacoronavirus 1 isolate Tb1** | **76.8%** |
| MW924112 Alphacoronavirus Korea 2020 Eptesicus serotinus HCQD2020 | NC018871 Alphacoronavirus Decacovirus China 2005 Rousettus bat HKU10 | 76.8% |
| MW924112 Alphacoronavirus Korea 2020 Eptesicus serotinus HCQD2020 | NC046964 Alphacoronavirus Nyctacovirus Italy 2015 Pipistrellus kuhlii | 76.8% |
| NC010438 Alphacoronavirus Minunacovirus Hong Kong 2004 Miniopterus bat HKU8 | OL410607 Alphacoronavirus U.S. 2020 Eptesicus fuscus | 76.8% |
| NC028752 Alphacoronavirus Duvinacovirus Saudi Arabia 2015 Camel | MZ293744 Alphacoronavirus Brazil 2014 Gardnerycteris crenulatum bat | 76.8% |
| MW924112 Alphacoronavirus Korea 2020 Eptesicus serotinus HCQD2020 | NC032107 Alphacoronavirus Setracovirus Kenia 2010 Triaenops afer NL63 related bat coronavirus BtKYNL63 9a | 76.8% |
| MT663548 Alphacoronavirus Peru 2015 Desmodus rotundus | NC002645 Alphacoronavirus Duvinacovirus Human coronavirus 229E | 76.7% |
| MW249018 Coronavirinae Peru 2016 Desmodus rotundus | NC002645 Alphacoronavirus Duvinacovirus Human coronavirus 229E | 76.7% |
| MT663548 Alphacoronavirus Peru 2015 Desmodus rotundus | NC028811 Alphacoronavirus Myotacovirus China 2011 Myotis ricketti SAX2011 | 76.7% |
| **NC028833 Alphacoronavirus Nyctacovirus China 2013 Nyctalus velutinus bat** | **OP715781 Tadarida brasiliensis bat alphacoronavirus 1 isolate Tb1** | **76.7%** |
| MW924112 Alphacoronavirus Korea 2020 Eptesicus serotinus HCQD2020 | NC028814 Alphacoronavirus Decacovirus China 2013 Rhinolophus ferrumequinum HuB2013 | 76.7% |
| MN535734 Coronavirinae Denmark 2016 Myotis dasycneme | MT663548 Alphacoronavirus Peru 2015 Desmodus rotundus | 76.7% |
| NC010438 Alphacoronavirus Minunacovirus Hong Kong 2004 Miniopterus bat HKU8 | OL410609 Alphacoronavirus U.S. 2020 Eptesicus fuscus | 76.7% |
| NC010438 Alphacoronavirus Minunacovirus Hong Kong 2004 Miniopterus bat HKU8 | OL415262 Alphacoronavirus U.S. 2021 Eptesicus fuscus | 76.7% |
| **NC010438 Alphacoronavirus Minunacovirus Hong Kong 2004 Miniopterus bat HKU8** | **OP715781 Tadarida brasiliensis bat alphacoronavirus 1 isolate Tb1** | **76.7%** |
| MW249018 Coronavirinae Peru 2016 Desmodus rotundus | NC028811 Alphacoronavirus Myotacovirus China 2011 Myotis ricketti SAX2011 | 76.6% |
| NC009988 Alphacoronavirus Rhinacovirus China 2006 Rhinolophus bat HKU2 | NC028833 Alphacoronavirus Nyctacovirus China 2013 Nyctalus velutinus bat | 76.6% |
| MW924112 Alphacoronavirus Korea 2020 Eptesicus serotinus HCQD2020 | NC028833 Alphacoronavirus Nyctacovirus China 2013 Nyctalus velutinus bat | 76.6% |
| NC002645 Alphacoronavirus Duvinacovirus Human coronavirus 229E | MZ293744 Alphacoronavirus Brazil 2014 Gardnerycteris crenulatum bat | 76.6% |
| NC005831 Alphacoronavirus Setracovirus Netherlands 2002 Human NL63 | MZ293744 Alphacoronavirus Brazil 2014 Gardnerycteris crenulatum bat | 76.6% |
| MT663548 Alphacoronavirus Peru 2015 Desmodus rotundus | MZ328298 Alphacoronavirus China 2016 Myotis chinensis | 76.5% |
| NC028824 Alphacoronavirus Rhinacovirus China 2013 Rhinolophus ferrumequinum YN2012 | NC028833 Alphacoronavirus Nyctacovirus China 2013 Nyctalus velutinus bat | 76.5% |
| MN535734 Coronavirinae Denmark 2016 Myotis dasycneme | MW249018 Coronavirinae Peru 2016 Desmodus rotundus | 76.5% |
| NC005831 Alphacoronavirus Setracovirus Netherlands 2002 Human NL63 | NC009657 Alphacoronavirus Pedacovirus China 2005 Scotophilus bat | 76.5% |
| NC009988 Alphacoronavirus Rhinacovirus China 2006 Rhinolophus bat HKU2 | OL410607 Alphacoronavirus U.S. 2020 Eptesicus fuscus | 76.5% |
| NC009988 Alphacoronavirus Rhinacovirus China 2006 Rhinolophus bat HKU2 | OL410609 Alphacoronavirus U.S. 2020 Eptesicus fuscus | 76.5% |
| **OL410607 Alphacoronavirus U.S. 2020 Eptesicus fuscus** | **OP700657 Tadarida brasilensis bat alphacoronavirus 2 isolate Tb3** | **76.5%** |
| **OL410609 Alphacoronavirus U.S. 2020 Eptesicus fuscus** | **OP700657 Tadarida brasilensis bat alphacoronavirus 2 isolate Tb3** | **76.5%** |
| **OL415262 Alphacoronavirus U.S. 2021 Eptesicus fuscus** | **OP700657 Tadarida brasilensis bat alphacoronavirus 2 isolate Tb3** | **76.5%** |
| **OL410607 Alphacoronavirus U.S. 2020 Eptesicus fuscus** | **OP715780 Tadarida brasilensis bat alphacoronavirus 2 isolate Tb2** | **76.5%** |
| **OL410609 Alphacoronavirus U.S. 2020 Eptesicus fuscus** | **OP715780 Tadarida brasilensis bat alphacoronavirus 2 isolate Tb2** | **76.5%** |
| **OL415262 Alphacoronavirus U.S. 2021 Eptesicus fuscus** | **OP715780 Tadarida brasilensis bat alphacoronavirus 2 isolate Tb2** | **76.5%** |
| MW924112 Alphacoronavirus Korea 2020 Eptesicus serotinus HCQD2020 | NC010438 Alphacoronavirus Minunacovirus Hong Kong 2004 Miniopterus bat HKU8 | 76.4% |
| MW249018 Coronavirinae Peru 2016 Desmodus rotundus | MZ328298 Alphacoronavirus China 2016 Myotis chinensis | 76.4% |
| **NC009988 Alphacoronavirus Rhinacovirus China 2006 Rhinolophus bat HKU2** | **OP715781 Tadarida brasiliensis bat alphacoronavirus 1 isolate Tb1** | **76.4%** |
| NC028824 Alphacoronavirus Rhinacovirus China 2013 Rhinolophus ferrumequinum YN2012 | OL410607 Alphacoronavirus U.S. 2020 Eptesicus fuscus | 76.3% |
| NC028824 Alphacoronavirus Rhinacovirus China 2013 Rhinolophus ferrumequinum YN2012 | OL410609 Alphacoronavirus U.S. 2020 Eptesicus fuscus | 76.3% |
| NC009988 Alphacoronavirus Rhinacovirus China 2006 Rhinolophus bat HKU2 | OL415262 Alphacoronavirus U.S. 2021 Eptesicus fuscus | 76.3% |
| **OP700657 Tadarida brasilensis bat alphacoronavirus 2 isolate Tb3** | **OP715781 Tadarida brasiliensis bat alphacoronavirus 1 isolate Tb1** | **76.3%** |
| **OP715780 Tadarida brasilensis bat alphacoronavirus 2 isolate Tb2** | **OP715781 Tadarida brasiliensis bat alphacoronavirus 1 isolate Tb1** | **76.3%** |
| MT663548 Alphacoronavirus Peru 2015 Desmodus rotundus | NC005831 Alphacoronavirus Setracovirus Netherlands 2002 Human NL63 | 76.3% |
| NC003436 Alphacoronavirus Pedacovirus Porcine | NC009988 Alphacoronavirus Rhinacovirus China 2006 Rhinolophus bat HKU2 | 76.3% |
| NC028752 Alphacoronavirus Duvinacovirus Saudi Arabia 2015 Camel | OL410609 Alphacoronavirus U.S. 2020 Eptesicus fuscus | 76.3% |
| NC003436 Alphacoronavirus Pedacovirus Porcine | NC028824 Alphacoronavirus Rhinacovirus China 2013 Rhinolophus ferrumequinum YN2012 | 76.2% |
| NC028824 Alphacoronavirus Rhinacovirus China 2013 Rhinolophus ferrumequinum YN2012 | OL415262 Alphacoronavirus U.S. 2021 Eptesicus fuscus | 76.2% |
| **NC028752 Alphacoronavirus Duvinacovirus Saudi Arabia 2015 Camel** | **OP715781 Tadarida brasiliensis bat alphacoronavirus 1 isolate Tb1** | **76.2%** |
| **NC028824 Alphacoronavirus Rhinacovirus China 2013 Rhinolophus ferrumequinum YN2012** | **OP715781 Tadarida brasiliensis bat alphacoronavirus 1 isolate Tb1** | **76.2%** |
| NC028752 Alphacoronavirus Duvinacovirus Saudi Arabia 2015 Camel | OL415262 Alphacoronavirus U.S. 2021 Eptesicus fuscus | 76.2% |
| MW924112 Alphacoronavirus Korea 2020 Eptesicus serotinus HCQD2020 | NC009988 Alphacoronavirus Rhinacovirus China 2006 Rhinolophus bat HKU2 | 76.2% |
| MW249018 Coronavirinae Peru 2016 Desmodus rotundus | NC005831 Alphacoronavirus Setracovirus Netherlands 2002 Human NL63 | 76.1% |
| NC028752 Alphacoronavirus Duvinacovirus Saudi Arabia 2015 Camel | OL410607 Alphacoronavirus U.S. 2020 Eptesicus fuscus | 76.1% |
| **NC005831 Alphacoronavirus Setracovirus Netherlands 2002 Human NL63** | **OP715781 Tadarida brasiliensis bat alphacoronavirus 1 isolate Tb1** | **76.1%** |
| **MW924112 Alphacoronavirus Korea 2020 Eptesicus serotinus HCQD2020** | **OP700657 Tadarida brasilensis bat alphacoronavirus 2 isolate Tb3** | **76.1%** |
| **MW924112 Alphacoronavirus Korea 2020 Eptesicus serotinus HCQD2020** | **OP715780 Tadarida brasilensis bat alphacoronavirus 2 isolate Tb2** | **76.1%** |
| NC009988 Alphacoronavirus Rhinacovirus China 2006 Rhinolophus bat HKU2 | NC022103 Alphacoronavirus Colacovirus U.S. 2006 Myotis lucifugus | 76.1% |
| MT663548 Alphacoronavirus Peru 2015 Desmodus rotundus | OL410607 Alphacoronavirus U.S. 2020 Eptesicus fuscus | 76.1% |
| MW249018 Coronavirinae Peru 2016 Desmodus rotundus | OL410607 Alphacoronavirus U.S. 2020 Eptesicus fuscus | 76.1% |
| NC002645 Alphacoronavirus Duvinacovirus Human coronavirus 229E | OL410609 Alphacoronavirus U.S. 2020 Eptesicus fuscus | 76.1% |
| MT663548 Alphacoronavirus Peru 2015 Desmodus rotundus | OL415262 Alphacoronavirus U.S. 2021 Eptesicus fuscus | 76.1% |
| MW249018 Coronavirinae Peru 2016 Desmodus rotundus | OL415262 Alphacoronavirus U.S. 2021 Eptesicus fuscus | 76.1% |
| NC009657 Alphacoronavirus Pedacovirus China 2005 Scotophilus bat | MZ293744 Alphacoronavirus Brazil 2014 Gardnerycteris crenulatum bat | 76.1% |
| MW924112 Alphacoronavirus Korea 2020 Eptesicus serotinus HCQD2020 | NC028824 Alphacoronavirus Rhinacovirus China 2013 Rhinolophus ferrumequinum YN2012 | 76.1% |
| NC022103 Alphacoronavirus Colacovirus U.S. 2006 Myotis lucifugus | OL410609 Alphacoronavirus U.S. 2020 Eptesicus fuscus | 76.0% |
| **NC002645 Alphacoronavirus Duvinacovirus Human coronavirus 229E** | **OP715781 Tadarida brasiliensis bat alphacoronavirus 1 isolate Tb1** | **76.0%** |
| MW249018 Coronavirinae Peru 2016 Desmodus rotundus | NC009657 Alphacoronavirus Pedacovirus China 2005 Scotophilus bat | 76.0% |
| NC009988 Alphacoronavirus Rhinacovirus China 2006 Rhinolophus bat HKU2 | NC023760 Alphacoronavirus Minacovirus U.S. 1998 Mustela vison | 76.0% |
| NC022103 Alphacoronavirus Colacovirus U.S. 2006 Myotis lucifugus | OL410607 Alphacoronavirus U.S. 2020 Eptesicus fuscus | 76.0% |
| MT663548 Alphacoronavirus Peru 2015 Desmodus rotundus | OL410609 Alphacoronavirus U.S. 2020 Eptesicus fuscus | 76.0% |
| MW249018 Coronavirinae Peru 2016 Desmodus rotundus | OL410609 Alphacoronavirus U.S. 2020 Eptesicus fuscus | 76.0% |
| NC002645 Alphacoronavirus Duvinacovirus Human coronavirus 229E | OL415262 Alphacoronavirus U.S. 2021 Eptesicus fuscus | 76.0% |
| NC022103 Alphacoronavirus Colacovirus U.S. 2006 Myotis lucifugus | OL415262 Alphacoronavirus U.S. 2021 Eptesicus fuscus | 76.0% |
| MT663548 Alphacoronavirus Peru 2015 Desmodus rotundus | NC009657 Alphacoronavirus Pedacovirus China 2005 Scotophilus bat | 75.9% |
| NC023760 Alphacoronavirus Minacovirus U.S. 1998 Mustela vison | NC028824 Alphacoronavirus Rhinacovirus China 2013 Rhinolophus ferrumequinum YN2012 | 75.9% |
| NC002645 Alphacoronavirus Duvinacovirus Human coronavirus 229E | OL410607 Alphacoronavirus U.S. 2020 Eptesicus fuscus | 75.9% |
| NC003436 Alphacoronavirus Pedacovirus Porcine | OL410607 Alphacoronavirus U.S. 2020 Eptesicus fuscus | 75.9% |
| NC003436 Alphacoronavirus Pedacovirus Porcine | OL410609 Alphacoronavirus U.S. 2020 Eptesicus fuscus | 75.9% |
| **NC022103 Alphacoronavirus Colacovirus U.S. 2006 Myotis lucifugus** | **OP715781 Tadarida brasiliensis bat alphacoronavirus 1 isolate Tb1** | **75.9%** |
| NC009657 Alphacoronavirus Pedacovirus China 2005 Scotophilus bat | NC028752 Alphacoronavirus Duvinacovirus Saudi Arabia 2015 Camel | 75.8% |
| NC022103 Alphacoronavirus Colacovirus U.S. 2006 Myotis lucifugus | NC028824 Alphacoronavirus Rhinacovirus China 2013 Rhinolophus ferrumequinum YN2012 | 75.8% |
| NC005831 Alphacoronavirus Setracovirus Netherlands 2002 Human NL63 | OL410609 Alphacoronavirus U.S. 2020 Eptesicus fuscus | 75.8% |
| NC003436 Alphacoronavirus Pedacovirus Porcine | OL415262 Alphacoronavirus U.S. 2021 Eptesicus fuscus | 75.8% |
| MT663548 Alphacoronavirus Peru 2015 Desmodus rotundus | MW924112 Alphacoronavirus Korea 2020 Eptesicus serotinus HCQD2020 | 75.8% |
| MW249018 Coronavirinae Peru 2016 Desmodus rotundus | MW924112 Alphacoronavirus Korea 2020 Eptesicus serotinus HCQD2020 | 75.8% |
| MW924112 Alphacoronavirus Korea 2020 Eptesicus serotinus HCQD2020 | NC028752 Alphacoronavirus Duvinacovirus Saudi Arabia 2015 Camel | 75.8% |
| NC022103 Alphacoronavirus Colacovirus U.S. 2006 Myotis lucifugus | NC028752 Alphacoronavirus Duvinacovirus Saudi Arabia 2015 Camel | 75.7% |
| NC005831 Alphacoronavirus Setracovirus Netherlands 2002 Human NL63 | OL415262 Alphacoronavirus U.S. 2021 Eptesicus fuscus | 75.7% |
| MW249018 Coronavirinae Peru 2016 Desmodus rotundus | NC009988 Alphacoronavirus Rhinacovirus China 2006 Rhinolophus bat HKU2 | 75.7% |
| MT663548 Alphacoronavirus Peru 2015 Desmodus rotundus | NC028824 Alphacoronavirus Rhinacovirus China 2013 Rhinolophus ferrumequinum YN2012 | 75.7% |
| NC009988 Alphacoronavirus Rhinacovirus China 2006 Rhinolophus bat HKU2 | NC030292 Alphacoronavirus Minacovirus Netherlands 2010 Mustela putorius ferret | 75.7% |
| NC005831 Alphacoronavirus Setracovirus Netherlands 2002 Human NL63 | OL410607 Alphacoronavirus U.S. 2020 Eptesicus fuscus | 75.7% |
| OL410607 Alphacoronavirus U.S. 2020 Eptesicus fuscus | MZ293744 Alphacoronavirus Brazil 2014 Gardnerycteris crenulatum bat | 75.7% |
| OL415262 Alphacoronavirus U.S. 2021 Eptesicus fuscus | MZ293744 Alphacoronavirus Brazil 2014 Gardnerycteris crenulatum bat | 75.7% |
| MT663548 Alphacoronavirus Peru 2015 Desmodus rotundus | NC009988 Alphacoronavirus Rhinacovirus China 2006 Rhinolophus bat HKU2 | 75.6% |
| MW249018 Coronavirinae Peru 2016 Desmodus rotundus | NC028824 Alphacoronavirus Rhinacovirus China 2013 Rhinolophus ferrumequinum YN2012 | 75.6% |
| NC009988 Alphacoronavirus Rhinacovirus China 2006 Rhinolophus bat HKU2 | MZ293744 Alphacoronavirus Brazil 2014 Gardnerycteris crenulatum bat | 75.6% |
| OL410609 Alphacoronavirus U.S. 2020 Eptesicus fuscus | MZ293744 Alphacoronavirus Brazil 2014 Gardnerycteris crenulatum bat | 75.6% |
| MW924112 Alphacoronavirus Korea 2020 Eptesicus serotinus HCQD2020 | NC022103 Alphacoronavirus Colacovirus U.S. 2006 Myotis lucifugus | 75.6% |
| NC002645 Alphacoronavirus Duvinacovirus Human coronavirus 229E | NC009657 Alphacoronavirus Pedacovirus China 2005 Scotophilus bat | 75.6% |
| NC028824 Alphacoronavirus Rhinacovirus China 2013 Rhinolophus ferrumequinum YN2012 | NC030292 Alphacoronavirus Minacovirus Netherlands 2010 Mustela putorius ferret | 75.6% |
| MW924112 Alphacoronavirus Korea 2020 Eptesicus serotinus HCQD2020 | NC002645 Alphacoronavirus Duvinacovirus Human coronavirus 229E | 75.6% |
| **NC003436 Alphacoronavirus Pedacovirus Porcine** | **OP715781 Tadarida brasiliensis bat alphacoronavirus 1 isolate Tb1** | **75.5%** |
| NC028824 Alphacoronavirus Rhinacovirus China 2013 Rhinolophus ferrumequinum YN2012 | MZ293744 Alphacoronavirus Brazil 2014 Gardnerycteris crenulatum bat | 75.5% |
| MW924112 Alphacoronavirus Korea 2020 Eptesicus serotinus HCQD2020 | NC005831 Alphacoronavirus Setracovirus Netherlands 2002 Human NL63 | 75.4% |
| MW924112 Alphacoronavirus Korea 2020 Eptesicus serotinus HCQD2020 | MZ293744 Alphacoronavirus Brazil 2014 Gardnerycteris crenulatum bat | 75.4% |
| **OP715781 Tadarida brasiliensis bat alphacoronavirus 1 isolate Tb1** | **MZ293744 Alphacoronavirus Brazil 2014 Gardnerycteris crenulatum bat** | **75.4%** |
| MW924112 Alphacoronavirus Korea 2020 Eptesicus serotinus HCQD2020 | NC003436 Alphacoronavirus Pedacovirus Porcine | 75.4% |
| NC009657 Alphacoronavirus Pedacovirus China 2005 Scotophilus bat | NC009988 Alphacoronavirus Rhinacovirus China 2006 Rhinolophus bat HKU2 | 75.4% |
| **MT663548 Alphacoronavirus Peru 2015 Desmodus rotundus** | **OP715781 Tadarida brasiliensis bat alphacoronavirus 1 isolate Tb1** | **75.4%** |
| **MW249018 Coronavirinae Peru 2016 Desmodus rotundus** | **OP715781 Tadarida brasiliensis bat alphacoronavirus 1 isolate Tb1** | **75.4%** |
| NC009657 Alphacoronavirus Pedacovirus China 2005 Scotophilus bat | NC028824 Alphacoronavirus Rhinacovirus China 2013 Rhinolophus ferrumequinum YN2012 | 75.3% |
| NC002645 Alphacoronavirus Duvinacovirus Human coronavirus 229E | NC022103 Alphacoronavirus Colacovirus U.S. 2006 Myotis lucifugus | 75.2% |
| MT663548 Alphacoronavirus Peru 2015 Desmodus rotundus | NC022103 Alphacoronavirus Colacovirus U.S. 2006 Myotis lucifugus | 75.1% |
| MW249018 Coronavirinae Peru 2016 Desmodus rotundus | NC022103 Alphacoronavirus Colacovirus U.S. 2006 Myotis lucifugus | 75.1% |
| NC009657 Alphacoronavirus Pedacovirus China 2005 Scotophilus bat | OL410607 Alphacoronavirus U.S. 2020 Eptesicus fuscus | 75.1% |
| NC009657 Alphacoronavirus Pedacovirus China 2005 Scotophilus bat | OL415262 Alphacoronavirus U.S. 2021 Eptesicus fuscus | 75.0% |
| **NC009657 Alphacoronavirus Pedacovirus China 2005 Scotophilus bat** | **OP715781 Tadarida brasiliensis bat alphacoronavirus 1 isolate Tb1** | **75.0%** |
| NC009657 Alphacoronavirus Pedacovirus China 2005 Scotophilus bat | OL410609 Alphacoronavirus U.S. 2020 Eptesicus fuscus | 75.0% |
| NC022103 Alphacoronavirus Colacovirus U.S. 2006 Myotis lucifugus | MZ293744 Alphacoronavirus Brazil 2014 Gardnerycteris crenulatum bat | 74.8% |
| MW924112 Alphacoronavirus Korea 2020 Eptesicus serotinus HCQD2020 | NC009657 Alphacoronavirus Pedacovirus China 2005 Scotophilus bat | 74.6% |
| NC009988 Alphacoronavirus Rhinacovirus China 2006 Rhinolophus bat HKU2 | NC038861 Alphacoronavirus Tegacovirus U.S. Pig Transmissible gastroenteritis virus | 74.5% |
| NC028824 Alphacoronavirus Rhinacovirus China 2013 Rhinolophus ferrumequinum YN2012 | NC038861 Alphacoronavirus Tegacovirus U.S. Pig Transmissible gastroenteritis virus | 74.4% |
| NC010437 Alphacoronavirus Minunacovirus Hong Kong 2004 Miniopterus magnater | NC023760 Alphacoronavirus Minacovirus U.S. 1998 Mustela vison | 74.4% |
| NC002306 Alphacoronavirus Tegacovirus U.S. Feline | NC009988 Alphacoronavirus Rhinacovirus China 2006 Rhinolophus bat HKU2 | 74.3% |
| NC009988 Alphacoronavirus Rhinacovirus China 2006 Rhinolophus bat HKU2 | NC028806 Alphacoronavirus Tegacovirus Italy 2009 Swine | 74.2% |
| NC002306 Alphacoronavirus Tegacovirus U.S. Feline | NC028824 Alphacoronavirus Rhinacovirus China 2013 Rhinolophus ferrumequinum YN2012 | 74.2% |
| MZ081397 Alphacoronavirus China 2020 Myotis laniger | NC023760 Alphacoronavirus Minacovirus U.S. 1998 Mustela vison | 74.2% |
| NC028806 Alphacoronavirus Tegacovirus Italy 2009 Swine | NC028824 Alphacoronavirus Rhinacovirus China 2013 Rhinolophus ferrumequinum YN2012 | 74.1% |
| MZ328299 Alphacoronavirus China 2016 Miniopterus schreibersii | NC023760 Alphacoronavirus Minacovirus U.S. 1998 Mustela vison | 73.9% |
| NC018871 Alphacoronavirus Decacovirus China 2005 Rousettus bat HKU10 | NC038861 Alphacoronavirus Tegacovirus U.S. Pig Transmissible gastroenteritis virus | 73.9% |
| NC002306 Alphacoronavirus Tegacovirus U.S. Feline | NC018871 Alphacoronavirus Decacovirus China 2005 Rousettus bat HKU10 | 73.9% |
| MN535734 Coronavirinae Denmark 2016 Myotis dasycneme | NC023760 Alphacoronavirus Minacovirus U.S. 1998 Mustela vison | 73.9% |
| NC023760 Alphacoronavirus Minacovirus U.S. 1998 Mustela vison | NC032107 Alphacoronavirus Setracovirus Kenia 2010 Triaenops afer NL63 related bat coronavirus BtKYNL63 9a | 73.8% |
| NC023760 Alphacoronavirus Minacovirus U.S. 1998 Mustela vison | NC028752 Alphacoronavirus Duvinacovirus Saudi Arabia 2015 Camel | 73.8% |
| NC010437 Alphacoronavirus Minunacovirus Hong Kong 2004 Miniopterus magnater | NC030292 Alphacoronavirus Minacovirus Netherlands 2010 Mustela putorius ferret | 73.8% |
| NC023760 Alphacoronavirus Minacovirus U.S. 1998 Mustela vison | NC048216 Alphacoronavirus Setracovirus Kenya 2010 Triaenops afer bat NL63 related bat coronavirus BtKYNL63 9b | 73.7% |
| **NC023760 Alphacoronavirus Minacovirus U.S. 1998 Mustela vison** | **OP700657 Tadarida brasilensis bat alphacoronavirus 2 isolate Tb3** | **73.7%** |
| **NC023760 Alphacoronavirus Minacovirus U.S. 1998 Mustela vison** | **OP715780 Tadarida brasilensis bat alphacoronavirus 2 isolate Tb2** | **73.7%** |
| NC028814 Alphacoronavirus Decacovirus China 2013 Rhinolophus ferrumequinum HuB2013 | NC038861 Alphacoronavirus Tegacovirus U.S. Pig Transmissible gastroenteritis virus | 73.6% |
| MZ081397 Alphacoronavirus China 2020 Myotis laniger | NC030292 Alphacoronavirus Minacovirus Netherlands 2010 Mustela putorius ferret | 73.6% |
| NC002306 Alphacoronavirus Tegacovirus U.S. Feline | NC028814 Alphacoronavirus Decacovirus China 2013 Rhinolophus ferrumequinum HuB2013 | 73.5% |
| NC028752 Alphacoronavirus Duvinacovirus Saudi Arabia 2015 Camel | NC030292 Alphacoronavirus Minacovirus Netherlands 2010 Mustela putorius ferret | 73.5% |
| MZ081397 Alphacoronavirus China 2020 Myotis laniger | NC002306 Alphacoronavirus Tegacovirus U.S. Feline | 73.5% |
| NC002645 Alphacoronavirus Duvinacovirus Human coronavirus 229E | NC023760 Alphacoronavirus Minacovirus U.S. 1998 Mustela vison | 73.4% |
| NC018871 Alphacoronavirus Decacovirus China 2005 Rousettus bat HKU10 | NC028806 Alphacoronavirus Tegacovirus Italy 2009 Swine | 73.4% |
| NC023760 Alphacoronavirus Minacovirus U.S. 1998 Mustela vison | NC028811 Alphacoronavirus Myotacovirus China 2011 Myotis ricketti SAX2011 | 73.4% |
| MZ328298 Alphacoronavirus China 2016 Myotis chinensis | NC023760 Alphacoronavirus Minacovirus U.S. 1998 Mustela vison | 73.4% |
| MZ328299 Alphacoronavirus China 2016 Miniopterus schreibersii | NC030292 Alphacoronavirus Minacovirus Netherlands 2010 Mustela putorius ferret | 73.4% |
| NC010438 Alphacoronavirus Minunacovirus Hong Kong 2004 Miniopterus bat HKU8 | NC038861 Alphacoronavirus Tegacovirus U.S. Pig Transmissible gastroenteritis virus | 73.3% |
| MZ081397 Alphacoronavirus China 2020 Myotis laniger | NC038861 Alphacoronavirus Tegacovirus U.S. Pig Transmissible gastroenteritis virus | 73.3% |
| NC010438 Alphacoronavirus Minunacovirus Hong Kong 2004 Miniopterus bat HKU8 | NC023760 Alphacoronavirus Minacovirus U.S. 1998 Mustela vison | 73.3% |
| NC028806 Alphacoronavirus Tegacovirus Italy 2009 Swine | NC028814 Alphacoronavirus Decacovirus China 2013 Rhinolophus ferrumequinum HuB2013 | 73.3% |
| NC002645 Alphacoronavirus Duvinacovirus Human coronavirus 229E | NC030292 Alphacoronavirus Minacovirus Netherlands 2010 Mustela putorius ferret | 73.3% |
| NC030292 Alphacoronavirus Minacovirus Netherlands 2010 Mustela putorius ferret | NC048216 Alphacoronavirus Setracovirus Kenya 2010 Triaenops afer bat NL63 related bat coronavirus BtKYNL63 9b | 73.3% |
| NC023760 Alphacoronavirus Minacovirus U.S. 1998 Mustela vison | NC028814 Alphacoronavirus Decacovirus China 2013 Rhinolophus ferrumequinum HuB2013 | 73.2% |
| NC032107 Alphacoronavirus Setracovirus Kenia 2010 Triaenops afer NL63 related bat coronavirus BtKYNL63 9a | NC038861 Alphacoronavirus Tegacovirus U.S. Pig Transmissible gastroenteritis virus | 73.2% |
| MZ081397 Alphacoronavirus China 2020 Myotis laniger | NC028806 Alphacoronavirus Tegacovirus Italy 2009 Swine | 73.2% |
| NC005831 Alphacoronavirus Setracovirus Netherlands 2002 Human NL63 | NC023760 Alphacoronavirus Minacovirus U.S. 1998 Mustela vison | 73.1% |
| NC030292 Alphacoronavirus Minacovirus Netherlands 2010 Mustela putorius ferret | NC032107 Alphacoronavirus Setracovirus Kenia 2010 Triaenops afer NL63 related bat coronavirus BtKYNL63 9a | 73.1% |
| NC028806 Alphacoronavirus Tegacovirus Italy 2009 Swine | NC048216 Alphacoronavirus Setracovirus Kenya 2010 Triaenops afer bat NL63 related bat coronavirus BtKYNL63 9b | 73.1% |
| NC038861 Alphacoronavirus Tegacovirus U.S. Pig Transmissible gastroenteritis virus | NC048216 Alphacoronavirus Setracovirus Kenya 2010 Triaenops afer bat NL63 related bat coronavirus BtKYNL63 9b | 73.1% |
| NC023760 Alphacoronavirus Minacovirus U.S. 1998 Mustela vison | NC046964 Alphacoronavirus Nyctacovirus Italy 2015 Pipistrellus kuhlii | 73.1% |
| MZ328299 Alphacoronavirus China 2016 Miniopterus schreibersii | NC002306 Alphacoronavirus Tegacovirus U.S. Feline | 73.1% |
| NC010438 Alphacoronavirus Minunacovirus Hong Kong 2004 Miniopterus bat HKU8 | NC028806 Alphacoronavirus Tegacovirus Italy 2009 Swine | 73.1% |
| NC028814 Alphacoronavirus Decacovirus China 2013 Rhinolophus ferrumequinum HuB2013 | NC030292 Alphacoronavirus Minacovirus Netherlands 2010 Mustela putorius ferret | 73.1% |
| MZ328299 Alphacoronavirus China 2016 Miniopterus schreibersii | NC038861 Alphacoronavirus Tegacovirus U.S. Pig Transmissible gastroenteritis virus | 73.1% |
| NC002306 Alphacoronavirus Tegacovirus U.S. Feline | NC003436 Alphacoronavirus Pedacovirus Porcine | 72.9% |
| NC002306 Alphacoronavirus Tegacovirus U.S. Feline | NC010438 Alphacoronavirus Minunacovirus Hong Kong 2004 Miniopterus bat HKU8 | 72.9% |
| NC010437 Alphacoronavirus Minunacovirus Hong Kong 2004 Miniopterus magnater | NC038861 Alphacoronavirus Tegacovirus U.S. Pig Transmissible gastroenteritis virus | 72.9% |
| NC002306 Alphacoronavirus Tegacovirus U.S. Feline | NC010437 Alphacoronavirus Minunacovirus Hong Kong 2004 Miniopterus magnater | 72.9% |
| MZ328299 Alphacoronavirus China 2016 Miniopterus schreibersii | NC028806 Alphacoronavirus Tegacovirus Italy 2009 Swine | 72.9% |
| NC010438 Alphacoronavirus Minunacovirus Hong Kong 2004 Miniopterus bat HKU8 | NC030292 Alphacoronavirus Minacovirus Netherlands 2010 Mustela putorius ferret | 72.9% |
| NC023760 Alphacoronavirus Minacovirus U.S. 1998 Mustela vison | MZ293744 Alphacoronavirus Brazil 2014 Gardnerycteris crenulatum bat | 72.9% |
| NC018871 Alphacoronavirus Decacovirus China 2005 Rousettus bat HKU10 | NC023760 Alphacoronavirus Minacovirus U.S. 1998 Mustela vison | 72.8% |
| NC018871 Alphacoronavirus Decacovirus China 2005 Rousettus bat HKU10 | NC030292 Alphacoronavirus Minacovirus Netherlands 2010 Mustela putorius ferret | 72.8% |
| NC028806 Alphacoronavirus Tegacovirus Italy 2009 Swine | NC032107 Alphacoronavirus Setracovirus Kenia 2010 Triaenops afer NL63 related bat coronavirus BtKYNL63 9a | 72.8% |
| MN535734 Coronavirinae Denmark 2016 Myotis dasycneme | NC038861 Alphacoronavirus Tegacovirus U.S. Pig Transmissible gastroenteritis virus | 72.8% |
| NC003436 Alphacoronavirus Pedacovirus Porcine | NC038861 Alphacoronavirus Tegacovirus U.S. Pig Transmissible gastroenteritis virus | 72.8% |
| NC023760 Alphacoronavirus Minacovirus U.S. 1998 Mustela vison | NC034972 Alphacoronavirus Luchacovirus China 2011 Apodemus chevrieri rat AcCoV JC34 | 72.8% |
| NC002306 Alphacoronavirus Tegacovirus U.S. Feline | NC048216 Alphacoronavirus Setracovirus Kenya 2010 Triaenops afer bat NL63 related bat coronavirus BtKYNL63 9b | 72.8% |
| NC003436 Alphacoronavirus Pedacovirus Porcine | NC023760 Alphacoronavirus Minacovirus U.S. 1998 Mustela vison | 72.7% |
| MN535734 Coronavirinae Denmark 2016 Myotis dasycneme | NC028806 Alphacoronavirus Tegacovirus Italy 2009 Swine | 72.7% |
| MZ328298 Alphacoronavirus China 2016 Myotis chinensis | NC038861 Alphacoronavirus Tegacovirus U.S. Pig Transmissible gastroenteritis virus | 72.7% |
| NC028752 Alphacoronavirus Duvinacovirus Saudi Arabia 2015 Camel | NC038861 Alphacoronavirus Tegacovirus U.S. Pig Transmissible gastroenteritis virus | 72.7% |
| NC010437 Alphacoronavirus Minunacovirus Hong Kong 2004 Miniopterus magnater | NC028806 Alphacoronavirus Tegacovirus Italy 2009 Swine | 72.7% |
| MN535734 Coronavirinae Denmark 2016 Myotis dasycneme | NC030292 Alphacoronavirus Minacovirus Netherlands 2010 Mustela putorius ferret | 72.7% |
| NC002306 Alphacoronavirus Tegacovirus U.S. Feline | NC032107 Alphacoronavirus Setracovirus Kenia 2010 Triaenops afer NL63 related bat coronavirus BtKYNL63 9a | 72.7% |
| NC030292 Alphacoronavirus Minacovirus Netherlands 2010 Mustela putorius ferret | NC032730 Alphacoronavirus Luchacovirus China 2013 Rattus norvegicus | 72.7% |
| MZ328298 Alphacoronavirus China 2016 Myotis chinensis | NC028806 Alphacoronavirus Tegacovirus Italy 2009 Swine | 72.6% |
| NC003436 Alphacoronavirus Pedacovirus Porcine | NC028806 Alphacoronavirus Tegacovirus Italy 2009 Swine | 72.6% |
| NC023760 Alphacoronavirus Minacovirus U.S. 1998 Mustela vison | NC028833 Alphacoronavirus Nyctacovirus China 2013 Nyctalus velutinus bat | 72.6% |
| NC002645 Alphacoronavirus Duvinacovirus Human coronavirus 229E | NC038861 Alphacoronavirus Tegacovirus U.S. Pig Transmissible gastroenteritis virus | 72.6% |
| NC005831 Alphacoronavirus Setracovirus Netherlands 2002 Human NL63 | NC038861 Alphacoronavirus Tegacovirus U.S. Pig Transmissible gastroenteritis virus | 72.6% |
| NC028811 Alphacoronavirus Myotacovirus China 2011 Myotis ricketti SAX2011 | NC038861 Alphacoronavirus Tegacovirus U.S. Pig Transmissible gastroenteritis virus | 72.6% |
| NC023760 Alphacoronavirus Minacovirus U.S. 1998 Mustela vison | NC032730 Alphacoronavirus Luchacovirus China 2013 Rattus norvegicus | 72.6% |
| **NC038861 Alphacoronavirus Tegacovirus U.S. Pig Transmissible gastroenteritis virus** | **OP700657 Tadarida brasilensis bat alphacoronavirus 2 isolate Tb3** | **72.6%** |
| **NC038861 Alphacoronavirus Tegacovirus U.S. Pig Transmissible gastroenteritis virus** | **OP715780 Tadarida brasilensis bat alphacoronavirus 2 isolate Tb2** | **72.6%** |
| NC030292 Alphacoronavirus Minacovirus Netherlands 2010 Mustela putorius ferret | NC034972 Alphacoronavirus Luchacovirus China 2011 Apodemus chevrieri rat AcCoV JC34 | 72.5% |
| NC002645 Alphacoronavirus Duvinacovirus Human coronavirus 229E | NC028806 Alphacoronavirus Tegacovirus Italy 2009 Swine | 72.5% |
| NC005831 Alphacoronavirus Setracovirus Netherlands 2002 Human NL63 | NC028806 Alphacoronavirus Tegacovirus Italy 2009 Swine | 72.5% |
| NC028752 Alphacoronavirus Duvinacovirus Saudi Arabia 2015 Camel | NC028806 Alphacoronavirus Tegacovirus Italy 2009 Swine | 72.5% |
| NC028806 Alphacoronavirus Tegacovirus Italy 2009 Swine | NC028811 Alphacoronavirus Myotacovirus China 2011 Myotis ricketti SAX2011 | 72.5% |
| NC003436 Alphacoronavirus Pedacovirus Porcine | NC030292 Alphacoronavirus Minacovirus Netherlands 2010 Mustela putorius ferret | 72.5% |
| NC022103 Alphacoronavirus Colacovirus U.S. 2006 Myotis lucifugus | NC030292 Alphacoronavirus Minacovirus Netherlands 2010 Mustela putorius ferret | 72.5% |
| **NC030292 Alphacoronavirus Minacovirus Netherlands 2010 Mustela putorius ferret** | **OP700657 Tadarida brasilensis bat alphacoronavirus 2 isolate Tb3** | **72.5%** |
| **NC030292 Alphacoronavirus Minacovirus Netherlands 2010 Mustela putorius ferret** | **OP715780 Tadarida brasilensis bat alphacoronavirus 2 isolate Tb2** | **72.5%** |
| MZ081397 Alphacoronavirus China 2020 Myotis laniger | NC032730 Alphacoronavirus Luchacovirus China 2013 Rattus norvegicus | 72.5% |
| MW249018 Coronavirinae Peru 2016 Desmodus rotundus | NC023760 Alphacoronavirus Minacovirus U.S. 1998 Mustela vison | 72.5% |
| MZ328299 Alphacoronavirus China 2016 Miniopterus schreibersii | NC032730 Alphacoronavirus Luchacovirus China 2013 Rattus norvegicus | 72.5% |
| MT663548 Alphacoronavirus Peru 2015 Desmodus rotundus | NC023760 Alphacoronavirus Minacovirus U.S. 1998 Mustela vison | 72.4% |
| NC010438 Alphacoronavirus Minunacovirus Hong Kong 2004 Miniopterus bat HKU8 | NC032730 Alphacoronavirus Luchacovirus China 2013 Rattus norvegicus | 72.4% |
| MZ328298 Alphacoronavirus China 2016 Myotis chinensis | NC002306 Alphacoronavirus Tegacovirus U.S. Feline | 72.4% |
| NC005831 Alphacoronavirus Setracovirus Netherlands 2002 Human NL63 | NC030292 Alphacoronavirus Minacovirus Netherlands 2010 Mustela putorius ferret | 72.4% |
| **NC028806 Alphacoronavirus Tegacovirus Italy 2009 Swine** | **OP700657 Tadarida brasilensis bat alphacoronavirus 2 isolate Tb3** | **72.4%** |
| **NC028806 Alphacoronavirus Tegacovirus Italy 2009 Swine** | **OP715780 Tadarida brasilensis bat alphacoronavirus 2 isolate Tb2** | **72.4%** |
| NC038861 Alphacoronavirus Tegacovirus U.S. Pig Transmissible gastroenteritis virus | NC046964 Alphacoronavirus Nyctacovirus Italy 2015 Pipistrellus kuhlii | 72.4% |
| NC030292 Alphacoronavirus Minacovirus Netherlands 2010 Mustela putorius ferret | MZ293744 Alphacoronavirus Brazil 2014 Gardnerycteris crenulatum bat | 72.4% |
| NC010437 Alphacoronavirus Minunacovirus Hong Kong 2004 Miniopterus magnater | NC032730 Alphacoronavirus Luchacovirus China 2013 Rattus norvegicus | 72.4% |
| NC010438 Alphacoronavirus Minunacovirus Hong Kong 2004 Miniopterus bat HKU8 | NC034972 Alphacoronavirus Luchacovirus China 2011 Apodemus chevrieri rat AcCoV JC34 | 72.4% |
| MN535734 Coronavirinae Denmark 2016 Myotis dasycneme | NC002306 Alphacoronavirus Tegacovirus U.S. Feline | 72.3% |
| NC002306 Alphacoronavirus Tegacovirus U.S. Feline | NC028752 Alphacoronavirus Duvinacovirus Saudi Arabia 2015 Camel | 72.3% |
| MZ328298 Alphacoronavirus China 2016 Myotis chinensis | NC030292 Alphacoronavirus Minacovirus Netherlands 2010 Mustela putorius ferret | 72.3% |
| **NC002306 Alphacoronavirus Tegacovirus U.S. Feline** | **OP700657 Tadarida brasilensis bat alphacoronavirus 2 isolate Tb3** | **72.3%** |
| **NC002306 Alphacoronavirus Tegacovirus U.S. Feline** | **OP715780 Tadarida brasilensis bat alphacoronavirus 2 isolate Tb2** | **72.3%** |
| NC002306 Alphacoronavirus Tegacovirus U.S. Feline | NC046964 Alphacoronavirus Nyctacovirus Italy 2015 Pipistrellus kuhlii | 72.3% |
| NC002306 Alphacoronavirus Tegacovirus U.S. Feline | NC002645 Alphacoronavirus Duvinacovirus Human coronavirus 229E | 72.3% |
| NC028811 Alphacoronavirus Myotacovirus China 2011 Myotis ricketti SAX2011 | NC030292 Alphacoronavirus Minacovirus Netherlands 2010 Mustela putorius ferret | 72.3% |
| NC028806 Alphacoronavirus Tegacovirus Italy 2009 Swine | NC046964 Alphacoronavirus Nyctacovirus Italy 2015 Pipistrellus kuhlii | 72.3% |
| NC028806 Alphacoronavirus Tegacovirus Italy 2009 Swine | NC034972 Alphacoronavirus Luchacovirus China 2011 Apodemus chevrieri rat AcCoV JC34 | 72.3% |
| NC009657 Alphacoronavirus Pedacovirus China 2005 Scotophilus bat | NC023760 Alphacoronavirus Minacovirus U.S. 1998 Mustela vison | 72.2% |
| NC002306 Alphacoronavirus Tegacovirus U.S. Feline | NC028811 Alphacoronavirus Myotacovirus China 2011 Myotis ricketti SAX2011 | 72.2% |
| MW249018 Coronavirinae Peru 2016 Desmodus rotundus | NC030292 Alphacoronavirus Minacovirus Netherlands 2010 Mustela putorius ferret | 72.2% |
| MZ081397 Alphacoronavirus China 2020 Myotis laniger | NC034972 Alphacoronavirus Luchacovirus China 2011 Apodemus chevrieri rat AcCoV JC34 | 72.2% |
| NC010437 Alphacoronavirus Minunacovirus Hong Kong 2004 Miniopterus magnater | NC034972 Alphacoronavirus Luchacovirus China 2011 Apodemus chevrieri rat AcCoV JC34 | 72.1% |
| NC022103 Alphacoronavirus Colacovirus U.S. 2006 Myotis lucifugus | NC023760 Alphacoronavirus Minacovirus U.S. 1998 Mustela vison | 72.1% |
| NC028833 Alphacoronavirus Nyctacovirus China 2013 Nyctalus velutinus bat | NC030292 Alphacoronavirus Minacovirus Netherlands 2010 Mustela putorius ferret | 72.1% |
| NC023760 Alphacoronavirus Minacovirus U.S. 1998 Mustela vison | OL415262 Alphacoronavirus U.S. 2021 Eptesicus fuscus | 72.1% |
| MT663548 Alphacoronavirus Peru 2015 Desmodus rotundus | NC030292 Alphacoronavirus Minacovirus Netherlands 2010 Mustela putorius ferret | 72.1% |
| NC028806 Alphacoronavirus Tegacovirus Italy 2009 Swine | MZ293744 Alphacoronavirus Brazil 2014 Gardnerycteris crenulatum bat | 72.1% |
| NC038861 Alphacoronavirus Tegacovirus U.S. Pig Transmissible gastroenteritis virus | MZ293744 Alphacoronavirus Brazil 2014 Gardnerycteris crenulatum bat | 72.1% |
| MZ328299 Alphacoronavirus China 2016 Miniopterus schreibersii | NC034972 Alphacoronavirus Luchacovirus China 2011 Apodemus chevrieri rat AcCoV JC34 | 72.1% |
| NC028833 Alphacoronavirus Nyctacovirus China 2013 Nyctalus velutinus bat | NC038861 Alphacoronavirus Tegacovirus U.S. Pig Transmissible gastroenteritis virus | 72.1% |
| NC023760 Alphacoronavirus Minacovirus U.S. 1998 Mustela vison | OL410609 Alphacoronavirus U.S. 2020 Eptesicus fuscus | 72.1% |
| NC034972 Alphacoronavirus Luchacovirus China 2011 Apodemus chevrieri rat AcCoV JC34 | NC038861 Alphacoronavirus Tegacovirus U.S. Pig Transmissible gastroenteritis virus | 72.1% |
| NC002306 Alphacoronavirus Tegacovirus U.S. Feline | NC005831 Alphacoronavirus Setracovirus Netherlands 2002 Human NL63 | 72.0% |
| NC028806 Alphacoronavirus Tegacovirus Italy 2009 Swine | NC028833 Alphacoronavirus Nyctacovirus China 2013 Nyctalus velutinus bat | 72.0% |
| NC009657 Alphacoronavirus Pedacovirus China 2005 Scotophilus bat | NC038861 Alphacoronavirus Tegacovirus U.S. Pig Transmissible gastroenteritis virus | 72.0% |
| NC023760 Alphacoronavirus Minacovirus U.S. 1998 Mustela vison | OL410607 Alphacoronavirus U.S. 2020 Eptesicus fuscus | 72.0% |
| MN535734 Coronavirinae Denmark 2016 Myotis dasycneme | NC032730 Alphacoronavirus Luchacovirus China 2013 Rattus norvegicus | 72.0% |
| NC028814 Alphacoronavirus Decacovirus China 2013 Rhinolophus ferrumequinum HuB2013 | NC032730 Alphacoronavirus Luchacovirus China 2013 Rattus norvegicus | 72.0% |
| NC028806 Alphacoronavirus Tegacovirus Italy 2009 Swine | NC032730 Alphacoronavirus Luchacovirus China 2013 Rattus norvegicus | 71.9% |
| NC032730 Alphacoronavirus Luchacovirus China 2013 Rattus norvegicus | NC038861 Alphacoronavirus Tegacovirus U.S. Pig Transmissible gastroenteritis virus | 71.9% |
| MW924112 Alphacoronavirus Korea 2020 Eptesicus serotinus HCQD2020 | NC023760 Alphacoronavirus Minacovirus U.S. 1998 Mustela vison | 71.9% |
| NC028824 Alphacoronavirus Rhinacovirus China 2013 Rhinolophus ferrumequinum YN2012 | NC032730 Alphacoronavirus Luchacovirus China 2013 Rattus norvegicus | 71.9% |
| NC034972 Alphacoronavirus Luchacovirus China 2011 Apodemus chevrieri rat AcCoV JC34 | NC046964 Alphacoronavirus Nyctacovirus Italy 2015 Pipistrellus kuhlii | 71.9% |
| **NC023760 Alphacoronavirus Minacovirus U.S. 1998 Mustela vison** | **OP715781 Tadarida brasiliensis bat alphacoronavirus 1 isolate Tb1** | **71.9%** |
| NC002306 Alphacoronavirus Tegacovirus U.S. Feline | MZ293744 Alphacoronavirus Brazil 2014 Gardnerycteris crenulatum bat | 71.9% |
| NC028811 Alphacoronavirus Myotacovirus China 2011 Myotis ricketti SAX2011 | NC034972 Alphacoronavirus Luchacovirus China 2011 Apodemus chevrieri rat AcCoV JC34 | 71.9% |
| NC002306 Alphacoronavirus Tegacovirus U.S. Feline | NC028833 Alphacoronavirus Nyctacovirus China 2013 Nyctalus velutinus bat | 71.9% |
| NC009988 Alphacoronavirus Rhinacovirus China 2006 Rhinolophus bat HKU2 | NC032730 Alphacoronavirus Luchacovirus China 2013 Rattus norvegicus | 71.8% |
| MN535734 Coronavirinae Denmark 2016 Myotis dasycneme | NC034972 Alphacoronavirus Luchacovirus China 2011 Apodemus chevrieri rat AcCoV JC34 | 71.8% |
| NC009657 Alphacoronavirus Pedacovirus China 2005 Scotophilus bat | NC028806 Alphacoronavirus Tegacovirus Italy 2009 Swine | 71.8% |
| NC030292 Alphacoronavirus Minacovirus Netherlands 2010 Mustela putorius ferret | NC046964 Alphacoronavirus Nyctacovirus Italy 2015 Pipistrellus kuhlii | 71.8% |
| MT663548 Alphacoronavirus Peru 2015 Desmodus rotundus | NC028806 Alphacoronavirus Tegacovirus Italy 2009 Swine | 71.8% |
| MW249018 Coronavirinae Peru 2016 Desmodus rotundus | NC028806 Alphacoronavirus Tegacovirus Italy 2009 Swine | 71.8% |
| NC028811 Alphacoronavirus Myotacovirus China 2011 Myotis ricketti SAX2011 | NC032730 Alphacoronavirus Luchacovirus China 2013 Rattus norvegicus | 71.7% |
| NC009988 Alphacoronavirus Rhinacovirus China 2006 Rhinolophus bat HKU2 | NC034972 Alphacoronavirus Luchacovirus China 2011 Apodemus chevrieri rat AcCoV JC34 | 71.7% |
| NC028814 Alphacoronavirus Decacovirus China 2013 Rhinolophus ferrumequinum HuB2013 | NC034972 Alphacoronavirus Luchacovirus China 2011 Apodemus chevrieri rat AcCoV JC34 | 71.7% |
| NC028824 Alphacoronavirus Rhinacovirus China 2013 Rhinolophus ferrumequinum YN2012 | NC034972 Alphacoronavirus Luchacovirus China 2011 Apodemus chevrieri rat AcCoV JC34 | 71.7% |
| MT663548 Alphacoronavirus Peru 2015 Desmodus rotundus | NC038861 Alphacoronavirus Tegacovirus U.S. Pig Transmissible gastroenteritis virus | 71.7% |
| MW249018 Coronavirinae Peru 2016 Desmodus rotundus | NC038861 Alphacoronavirus Tegacovirus U.S. Pig Transmissible gastroenteritis virus | 71.7% |
| NC009657 Alphacoronavirus Pedacovirus China 2005 Scotophilus bat | NC030292 Alphacoronavirus Minacovirus Netherlands 2010 Mustela putorius ferret | 71.7% |
| MZ328298 Alphacoronavirus China 2016 Myotis chinensis | NC034972 Alphacoronavirus Luchacovirus China 2011 Apodemus chevrieri rat AcCoV JC34 | 71.6% |
| NC032107 Alphacoronavirus Setracovirus Kenia 2010 Triaenops afer NL63 related bat coronavirus BtKYNL63 9a | NC032730 Alphacoronavirus Luchacovirus China 2013 Rattus norvegicus | 71.6% |
| NC038861 Alphacoronavirus Tegacovirus U.S. Pig Transmissible gastroenteritis virus | OL410609 Alphacoronavirus U.S. 2020 Eptesicus fuscus | 71.6% |
| NC038861 Alphacoronavirus Tegacovirus U.S. Pig Transmissible gastroenteritis virus | OL415262 Alphacoronavirus U.S. 2021 Eptesicus fuscus | 71.6% |
| MZ328298 Alphacoronavirus China 2016 Myotis chinensis | NC032730 Alphacoronavirus Luchacovirus China 2013 Rattus norvegicus | 71.5% |
| NC002306 Alphacoronavirus Tegacovirus U.S. Feline | NC009657 Alphacoronavirus Pedacovirus China 2005 Scotophilus bat | 71.5% |
| **NC038861 Alphacoronavirus Tegacovirus U.S. Pig Transmissible gastroenteritis virus** | **OP715781 Tadarida brasiliensis bat alphacoronavirus 1 isolate Tb1** | **71.5%** |
| NC038861 Alphacoronavirus Tegacovirus U.S. Pig Transmissible gastroenteritis virus | OL410607 Alphacoronavirus U.S. 2020 Eptesicus fuscus | 71.5% |
| NC002306 Alphacoronavirus Tegacovirus U.S. Feline | NC034972 Alphacoronavirus Luchacovirus China 2011 Apodemus chevrieri rat AcCoV JC34 | 71.5% |
| MT663548 Alphacoronavirus Peru 2015 Desmodus rotundus | NC002306 Alphacoronavirus Tegacovirus U.S. Feline | 71.4% |
| MW249018 Coronavirinae Peru 2016 Desmodus rotundus | NC002306 Alphacoronavirus Tegacovirus U.S. Feline | 71.4% |
| NC018871 Alphacoronavirus Decacovirus China 2005 Rousettus bat HKU10 | NC034972 Alphacoronavirus Luchacovirus China 2011 Apodemus chevrieri rat AcCoV JC34 | 71.4% |
| NC022103 Alphacoronavirus Colacovirus U.S. 2006 Myotis lucifugus | NC038861 Alphacoronavirus Tegacovirus U.S. Pig Transmissible gastroenteritis virus | 71.4% |
| NC030292 Alphacoronavirus Minacovirus Netherlands 2010 Mustela putorius ferret | OL415262 Alphacoronavirus U.S. 2021 Eptesicus fuscus | 71.4% |
| NC002306 Alphacoronavirus Tegacovirus U.S. Feline | NC032730 Alphacoronavirus Luchacovirus China 2013 Rattus norvegicus | 71.4% |
| MW924112 Alphacoronavirus Korea 2020 Eptesicus serotinus HCQD2020 | NC038861 Alphacoronavirus Tegacovirus U.S. Pig Transmissible gastroenteritis virus | 71.4% |
| NC032107 Alphacoronavirus Setracovirus Kenia 2010 Triaenops afer NL63 related bat coronavirus BtKYNL63 9a | NC034972 Alphacoronavirus Luchacovirus China 2011 Apodemus chevrieri rat AcCoV JC34 | 71.4% |
| NC032730 Alphacoronavirus Luchacovirus China 2013 Rattus norvegicus | NC048216 Alphacoronavirus Setracovirus Kenya 2010 Triaenops afer bat NL63 related bat coronavirus BtKYNL63 9b | 71.4% |
| NC030292 Alphacoronavirus Minacovirus Netherlands 2010 Mustela putorius ferret | OL410609 Alphacoronavirus U.S. 2020 Eptesicus fuscus | 71.4% |
| NC032730 Alphacoronavirus Luchacovirus China 2013 Rattus norvegicus | NC046964 Alphacoronavirus Nyctacovirus Italy 2015 Pipistrellus kuhlii | 71.4% |
| NC028833 Alphacoronavirus Nyctacovirus China 2013 Nyctalus velutinus bat | NC034972 Alphacoronavirus Luchacovirus China 2011 Apodemus chevrieri rat AcCoV JC34 | 71.3% |
| NC030292 Alphacoronavirus Minacovirus Netherlands 2010 Mustela putorius ferret | OL410607 Alphacoronavirus U.S. 2020 Eptesicus fuscus | 71.3% |
| **NC028806 Alphacoronavirus Tegacovirus Italy 2009 Swine** | **OP715781 Tadarida brasiliensis bat alphacoronavirus 1 isolate Tb1** | **71.3%** |
| **NC030292 Alphacoronavirus Minacovirus Netherlands 2010 Mustela putorius ferret** | **OP715781 Tadarida brasiliensis bat alphacoronavirus 1 isolate Tb1** | **71.3%** |
| NC018871 Alphacoronavirus Decacovirus China 2005 Rousettus bat HKU10 | NC032730 Alphacoronavirus Luchacovirus China 2013 Rattus norvegicus | 71.3% |
| NC002306 Alphacoronavirus Tegacovirus U.S. Feline | NC022103 Alphacoronavirus Colacovirus U.S. 2006 Myotis lucifugus | 71.2% |
| NC028806 Alphacoronavirus Tegacovirus Italy 2009 Swine | OL410609 Alphacoronavirus U.S. 2020 Eptesicus fuscus | 71.2% |
| NC028806 Alphacoronavirus Tegacovirus Italy 2009 Swine | OL415262 Alphacoronavirus U.S. 2021 Eptesicus fuscus | 71.2% |
| MW924112 Alphacoronavirus Korea 2020 Eptesicus serotinus HCQD2020 | NC030292 Alphacoronavirus Minacovirus Netherlands 2010 Mustela putorius ferret | 71.2% |
| NC002306 Alphacoronavirus Tegacovirus U.S. Feline | OL410609 Alphacoronavirus U.S. 2020 Eptesicus fuscus | 71.2% |
| NC002306 Alphacoronavirus Tegacovirus U.S. Feline | OL415262 Alphacoronavirus U.S. 2021 Eptesicus fuscus | 71.2% |
| NC034972 Alphacoronavirus Luchacovirus China 2011 Apodemus chevrieri rat AcCoV JC34 | NC048216 Alphacoronavirus Setracovirus Kenya 2010 Triaenops afer bat NL63 related bat coronavirus BtKYNL63 9b | 71.1% |
| NC022103 Alphacoronavirus Colacovirus U.S. 2006 Myotis lucifugus | NC028806 Alphacoronavirus Tegacovirus Italy 2009 Swine | 71.1% |
| NC028806 Alphacoronavirus Tegacovirus Italy 2009 Swine | OL410607 Alphacoronavirus U.S. 2020 Eptesicus fuscus | 71.1% |
| **NC002306 Alphacoronavirus Tegacovirus U.S. Feline** | **OP715781 Tadarida brasiliensis bat alphacoronavirus 1 isolate Tb1** | **71.1%** |
| MW924112 Alphacoronavirus Korea 2020 Eptesicus serotinus HCQD2020 | NC028806 Alphacoronavirus Tegacovirus Italy 2009 Swine | 71.1% |
| NC002306 Alphacoronavirus Tegacovirus U.S. Feline | OL410607 Alphacoronavirus U.S. 2020 Eptesicus fuscus | 71.1% |
| MW924112 Alphacoronavirus Korea 2020 Eptesicus serotinus HCQD2020 | NC002306 Alphacoronavirus Tegacovirus U.S. Feline | 71.0% |
| NC028752 Alphacoronavirus Duvinacovirus Saudi Arabia 2015 Camel | NC034972 Alphacoronavirus Luchacovirus China 2011 Apodemus chevrieri rat AcCoV JC34 | 71.0% |
| **NC034972 Alphacoronavirus Luchacovirus China 2011 Apodemus chevrieri rat AcCoV JC34** | **OP700657 Tadarida brasilensis bat alphacoronavirus 2 isolate Tb3** | **70.9%** |
| **NC034972 Alphacoronavirus Luchacovirus China 2011 Apodemus chevrieri rat AcCoV JC34** | **OP715780 Tadarida brasilensis bat alphacoronavirus 2 isolate Tb2** | **70.9%** |
| NC005831 Alphacoronavirus Setracovirus Netherlands 2002 Human NL63 | NC032730 Alphacoronavirus Luchacovirus China 2013 Rattus norvegicus | 70.8% |
| NC028752 Alphacoronavirus Duvinacovirus Saudi Arabia 2015 Camel | NC032730 Alphacoronavirus Luchacovirus China 2013 Rattus norvegicus | 70.8% |
| NC028833 Alphacoronavirus Nyctacovirus China 2013 Nyctalus velutinus bat | NC032730 Alphacoronavirus Luchacovirus China 2013 Rattus norvegicus | 70.8% |
| **NC032730 Alphacoronavirus Luchacovirus China 2013 Rattus norvegicus** | **OP700657 Tadarida brasilensis bat alphacoronavirus 2 isolate Tb3** | **70.8%** |
| **NC032730 Alphacoronavirus Luchacovirus China 2013 Rattus norvegicus** | **OP715780 Tadarida brasilensis bat alphacoronavirus 2 isolate Tb2** | **70.8%** |
| NC032730 Alphacoronavirus Luchacovirus China 2013 Rattus norvegicus | MZ293744 Alphacoronavirus Brazil 2014 Gardnerycteris crenulatum bat | 70.7% |
| NC034972 Alphacoronavirus Luchacovirus China 2011 Apodemus chevrieri rat AcCoV JC34 | MZ293744 Alphacoronavirus Brazil 2014 Gardnerycteris crenulatum bat | 70.7% |
| MT663548 Alphacoronavirus Peru 2015 Desmodus rotundus | NC034972 Alphacoronavirus Luchacovirus China 2011 Apodemus chevrieri rat AcCoV JC34 | 70.6% |
| MW249018 Coronavirinae Peru 2016 Desmodus rotundus | NC034972 Alphacoronavirus Luchacovirus China 2011 Apodemus chevrieri rat AcCoV JC34 | 70.6% |
| NC002645 Alphacoronavirus Duvinacovirus Human coronavirus 229E | NC034972 Alphacoronavirus Luchacovirus China 2011 Apodemus chevrieri rat AcCoV JC34 | 70.5% |
| NC005831 Alphacoronavirus Setracovirus Netherlands 2002 Human NL63 | NC034972 Alphacoronavirus Luchacovirus China 2011 Apodemus chevrieri rat AcCoV JC34 | 70.5% |
| NC003436 Alphacoronavirus Pedacovirus Porcine | NC034972 Alphacoronavirus Luchacovirus China 2011 Apodemus chevrieri rat AcCoV JC34 | 70.4% |
| MT663548 Alphacoronavirus Peru 2015 Desmodus rotundus | NC032730 Alphacoronavirus Luchacovirus China 2013 Rattus norvegicus | 70.4% |
| MW249018 Coronavirinae Peru 2016 Desmodus rotundus | NC032730 Alphacoronavirus Luchacovirus China 2013 Rattus norvegicus | 70.4% |
| NC002645 Alphacoronavirus Duvinacovirus Human coronavirus 229E | NC032730 Alphacoronavirus Luchacovirus China 2013 Rattus norvegicus | 70.3% |
| NC022103 Alphacoronavirus Colacovirus U.S. 2006 Myotis lucifugus | NC034972 Alphacoronavirus Luchacovirus China 2011 Apodemus chevrieri rat AcCoV JC34 | 70.1% |
| **NC032730 Alphacoronavirus Luchacovirus China 2013 Rattus norvegicus** | **OP715781 Tadarida brasiliensis bat alphacoronavirus 1 isolate Tb1** | **70.1%** |
| NC003436 Alphacoronavirus Pedacovirus Porcine | NC032730 Alphacoronavirus Luchacovirus China 2013 Rattus norvegicus | 70.0% |
| NC034972 Alphacoronavirus Luchacovirus China 2011 Apodemus chevrieri rat AcCoV JC34 | OL410609 Alphacoronavirus U.S. 2020 Eptesicus fuscus | 70.0% |
| NC022103 Alphacoronavirus Colacovirus U.S. 2006 Myotis lucifugus | NC032730 Alphacoronavirus Luchacovirus China 2013 Rattus norvegicus | 69.9% |
| NC032730 Alphacoronavirus Luchacovirus China 2013 Rattus norvegicus | OL410609 Alphacoronavirus U.S. 2020 Eptesicus fuscus | 69.9% |
| NC034972 Alphacoronavirus Luchacovirus China 2011 Apodemus chevrieri rat AcCoV JC34 | OL415262 Alphacoronavirus U.S. 2021 Eptesicus fuscus | 69.9% |
| **NC034972 Alphacoronavirus Luchacovirus China 2011 Apodemus chevrieri rat AcCoV JC34** | **OP715781 Tadarida brasiliensis bat alphacoronavirus 1 isolate Tb1** | **69.9%** |
| NC032730 Alphacoronavirus Luchacovirus China 2013 Rattus norvegicus | OL415262 Alphacoronavirus U.S. 2021 Eptesicus fuscus | 69.9% |
| MW924112 Alphacoronavirus Korea 2020 Eptesicus serotinus HCQD2020 | NC034972 Alphacoronavirus Luchacovirus China 2011 Apodemus chevrieri rat AcCoV JC34 | 69.8% |
| NC034972 Alphacoronavirus Luchacovirus China 2011 Apodemus chevrieri rat AcCoV JC34 | OL410607 Alphacoronavirus U.S. 2020 Eptesicus fuscus | 69.8% |
| NC009657 Alphacoronavirus Pedacovirus China 2005 Scotophilus bat | NC032730 Alphacoronavirus Luchacovirus China 2013 Rattus norvegicus | 69.8% |
| NC009657 Alphacoronavirus Pedacovirus China 2005 Scotophilus bat | NC034972 Alphacoronavirus Luchacovirus China 2011 Apodemus chevrieri rat AcCoV JC34 | 69.8% |
| NC032730 Alphacoronavirus Luchacovirus China 2013 Rattus norvegicus | OL410607 Alphacoronavirus U.S. 2020 Eptesicus fuscus | 69.8% |
| MW924112 Alphacoronavirus Korea 2020 Eptesicus serotinus HCQD2020 | NC032730 Alphacoronavirus Luchacovirus China 2013 Rattus norvegicus | 69.7% |
| NC019843 Betacoronavirus Merbecovirus Saudi Arabia 2012 Human MERSCoV | NC045512 Betacoronavirus Sarbecovirus China 2019 Human SARS CoV 2 | 68.1% |
| MN996532 Betacoronavirus Sarbecovirus China 2013 Rhinolophus affinis RaTG13 | NC019843 Betacoronavirus Merbecovirus Saudi Arabia 2012 Human MERSCoV | 68.1% |
| NC028824 Alphacoronavirus Rhinacovirus China 2013 Rhinolophus ferrumequinum YN2012 | KY967715 Alphacoronavirus Sunacovirus China 2015 Suncus murinus musarania | 66.9% |
| NC009988 Alphacoronavirus Rhinacovirus China 2006 Rhinolophus bat HKU2 | KY967715 Alphacoronavirus Sunacovirus China 2015 Suncus murinus musarania | 66.6% |
| NC028814 Alphacoronavirus Decacovirus China 2013 Rhinolophus ferrumequinum HuB2013 | KY967715 Alphacoronavirus Sunacovirus China 2015 Suncus murinus musarania | 66.3% |
| NC018871 Alphacoronavirus Decacovirus China 2005 Rousettus bat HKU10 | KY967715 Alphacoronavirus Sunacovirus China 2015 Suncus murinus musarania | 66.2% |
| NC030292 Alphacoronavirus Minacovirus Netherlands 2010 Mustela putorius ferret | KY967715 Alphacoronavirus Sunacovirus China 2015 Suncus murinus musarania | 66.0% |
| NC010438 Alphacoronavirus Minunacovirus Hong Kong 2004 Miniopterus bat HKU8 | KY967715 Alphacoronavirus Sunacovirus China 2015 Suncus murinus musarania | 66.0% |
| NC048216 Alphacoronavirus Setracovirus Kenya 2010 Triaenops afer bat NL63 related bat coronavirus BtKYNL63 9b | KY967715 Alphacoronavirus Sunacovirus China 2015 Suncus murinus musarania | 66.0% |
| NC023760 Alphacoronavirus Minacovirus U.S. 1998 Mustela vison | KY967715 Alphacoronavirus Sunacovirus China 2015 Suncus murinus musarania | 65.9% |
| NC022103 Alphacoronavirus Colacovirus U.S. 2006 Myotis lucifugus | KY967715 Alphacoronavirus Sunacovirus China 2015 Suncus murinus musarania | 65.8% |
| NC005831 Alphacoronavirus Setracovirus Netherlands 2002 Human NL63 | KY967715 Alphacoronavirus Sunacovirus China 2015 Suncus murinus musarania | 65.7% |
| NC032107 Alphacoronavirus Setracovirus Kenia 2010 Triaenops afer NL63 related bat coronavirus BtKYNL63 9a | KY967715 Alphacoronavirus Sunacovirus China 2015 Suncus murinus musarania | 65.7% |
| NC006577 Betacoronavirus Embecovirus China 2004 Human HKU1 | NC019843 Betacoronavirus Merbecovirus Saudi Arabia 2012 Human MERSCoV | 65.7% |
| NC003436 Alphacoronavirus Pedacovirus Porcine | KY967715 Alphacoronavirus Sunacovirus China 2015 Suncus murinus musarania | 65.7% |
| NC038861 Alphacoronavirus Tegacovirus U.S. Pig Transmissible gastroenteritis virus | KY967715 Alphacoronavirus Sunacovirus China 2015 Suncus murinus musarania | 65.6% |
| NC010437 Alphacoronavirus Minunacovirus Hong Kong 2004 Miniopterus magnater | KY967715 Alphacoronavirus Sunacovirus China 2015 Suncus murinus musarania | 65.5% |
| **OP700657 Tadarida brasilensis bat alphacoronavirus 2 isolate Tb3** | **KY967715 Alphacoronavirus Sunacovirus China 2015 Suncus murinus musarania** | **65.5%** |
| **OP715780 Tadarida brasilensis bat alphacoronavirus 2 isolate Tb2** | **KY967715 Alphacoronavirus Sunacovirus China 2015 Suncus murinus musarania** | **65.5%** |
| NC028833 Alphacoronavirus Nyctacovirus China 2013 Nyctalus velutinus bat | KY967715 Alphacoronavirus Sunacovirus China 2015 Suncus murinus musarania | 65.4% |
| NC002306 Alphacoronavirus Tegacovirus U.S. Feline | KY967715 Alphacoronavirus Sunacovirus China 2015 Suncus murinus musarania | 65.4% |
| MZ081397 Alphacoronavirus China 2020 Myotis laniger | KY967715 Alphacoronavirus Sunacovirus China 2015 Suncus murinus musarania | 65.4% |
| NC028806 Alphacoronavirus Tegacovirus Italy 2009 Swine | KY967715 Alphacoronavirus Sunacovirus China 2015 Suncus murinus musarania | 65.3% |
| MZ328299 Alphacoronavirus China 2016 Miniopterus schreibersii | KY967715 Alphacoronavirus Sunacovirus China 2015 Suncus murinus musarania | 65.3% |
| NC034972 Alphacoronavirus Luchacovirus China 2011 Apodemus chevrieri rat AcCoV JC34 | KY967715 Alphacoronavirus Sunacovirus China 2015 Suncus murinus musarania | 65.3% |
| MW249018 Coronavirinae Peru 2016 Desmodus rotundus | KY967715 Alphacoronavirus Sunacovirus China 2015 Suncus murinus musarania | 65.0% |
| MT663548 Alphacoronavirus Peru 2015 Desmodus rotundus | KY967715 Alphacoronavirus Sunacovirus China 2015 Suncus murinus musarania | 64.9% |
| NC032730 Alphacoronavirus Luchacovirus China 2013 Rattus norvegicus | KY967715 Alphacoronavirus Sunacovirus China 2015 Suncus murinus musarania | 64.9% |
| NC046964 Alphacoronavirus Nyctacovirus Italy 2015 Pipistrellus kuhlii | KY967715 Alphacoronavirus Sunacovirus China 2015 Suncus murinus musarania | 64.8% |
| NC028752 Alphacoronavirus Duvinacovirus Saudi Arabia 2015 Camel | KY967715 Alphacoronavirus Sunacovirus China 2015 Suncus murinus musarania | 64.7% |
| NC009657 Alphacoronavirus Pedacovirus China 2005 Scotophilus bat | KY967715 Alphacoronavirus Sunacovirus China 2015 Suncus murinus musarania | 64.7% |
| MZ293744 Alphacoronavirus Brazil 2014 Gardnerycteris crenulatum bat | KY967715 Alphacoronavirus Sunacovirus China 2015 Suncus murinus musarania | 64.7% |
| NC002645 Alphacoronavirus Duvinacovirus Human coronavirus 229E | KY967715 Alphacoronavirus Sunacovirus China 2015 Suncus murinus musarania | 64.6% |
| OL410607 Alphacoronavirus U.S. 2020 Eptesicus fuscus | KY967715 Alphacoronavirus Sunacovirus China 2015 Suncus murinus musarania | 64.6% |
| OL410609 Alphacoronavirus U.S. 2020 Eptesicus fuscus | KY967715 Alphacoronavirus Sunacovirus China 2015 Suncus murinus musarania | 64.6% |
| OL415262 Alphacoronavirus U.S. 2021 Eptesicus fuscus | KY967715 Alphacoronavirus Sunacovirus China 2015 Suncus murinus musarania | 64.5% |
| MZ328298 Alphacoronavirus China 2016 Myotis chinensis | KY967715 Alphacoronavirus Sunacovirus China 2015 Suncus murinus musarania | 64.5% |
| MW924112 Alphacoronavirus Korea 2020 Eptesicus serotinus HCQD2020 | KY967715 Alphacoronavirus Sunacovirus China 2015 Suncus murinus musarania | 64.5% |
| NC028811 Alphacoronavirus Myotacovirus China 2011 Myotis ricketti SAX2011 | KY967715 Alphacoronavirus Sunacovirus China 2015 Suncus murinus musarania | 64.4% |
| **OP715781 Tadarida brasiliensis bat alphacoronavirus 1 isolate Tb1** | **KY967715 Alphacoronavirus Sunacovirus China 2015 Suncus murinus musarania** | **64.4%** |
| MN535734 Coronavirinae Denmark 2016 Myotis dasycneme | KY967715 Alphacoronavirus Sunacovirus China 2015 Suncus murinus musarania | 64.3% |
| NC006577 Betacoronavirus Embecovirus China 2004 Human HKU1 | NC045512 Betacoronavirus Sarbecovirus China 2019 Human SARS CoV 2 | 63.8% |
| MN996532 Betacoronavirus Sarbecovirus China 2013 Rhinolophus affinis RaTG13 | NC006577 Betacoronavirus Embecovirus China 2004 Human HKU1 | 63.6% |
| NC030292 Alphacoronavirus Minacovirus Netherlands 2010 Mustela putorius ferret | KY370053 Alphacoronavirus Soracovirus China 2014 Sorex araneus musarania | 63.1% |
| NC009988 Alphacoronavirus Rhinacovirus China 2006 Rhinolophus bat HKU2 | KY370053 Alphacoronavirus Soracovirus China 2014 Sorex araneus musarania | 62.3% |
| NC023760 Alphacoronavirus Minacovirus U.S. 1998 Mustela vison | KY370053 Alphacoronavirus Soracovirus China 2014 Sorex araneus musarania | 62.1% |
| NC028824 Alphacoronavirus Rhinacovirus China 2013 Rhinolophus ferrumequinum YN2012 | KY370053 Alphacoronavirus Soracovirus China 2014 Sorex araneus musarania | 62.1% |
| NC028806 Alphacoronavirus Tegacovirus Italy 2009 Swine | KY370053 Alphacoronavirus Soracovirus China 2014 Sorex araneus musarania | 61.9% |
| NC038861 Alphacoronavirus Tegacovirus U.S. Pig Transmissible gastroenteritis virus | KY370053 Alphacoronavirus Soracovirus China 2014 Sorex araneus musarania | 61.9% |
| NC002306 Alphacoronavirus Tegacovirus U.S. Feline | KY370053 Alphacoronavirus Soracovirus China 2014 Sorex araneus musarania | 61.9% |
| NC028833 Alphacoronavirus Nyctacovirus China 2013 Nyctalus velutinus bat | KY370053 Alphacoronavirus Soracovirus China 2014 Sorex araneus musarania | 61.4% |
| MZ081397 Alphacoronavirus China 2020 Myotis laniger | KY370053 Alphacoronavirus Soracovirus China 2014 Sorex araneus musarania | 61.4% |
| MZ328298 Alphacoronavirus China 2016 Myotis chinensis | KY370053 Alphacoronavirus Soracovirus China 2014 Sorex araneus musarania | 61.4% |
| MZ328299 Alphacoronavirus China 2016 Miniopterus schreibersii | KY370053 Alphacoronavirus Soracovirus China 2014 Sorex araneus musarania | 61.3% |
| MN535734 Coronavirinae Denmark 2016 Myotis dasycneme | KY370053 Alphacoronavirus Soracovirus China 2014 Sorex araneus musarania | 61.3% |
| NC028811 Alphacoronavirus Myotacovirus China 2011 Myotis ricketti SAX2011 | KY370053 Alphacoronavirus Soracovirus China 2014 Sorex araneus musarania | 61.3% |
| NC002645 Alphacoronavirus Duvinacovirus Human coronavirus 229E | KY370053 Alphacoronavirus Soracovirus China 2014 Sorex araneus musarania | 61.1% |
| NC010437 Alphacoronavirus Minunacovirus Hong Kong 2004 Miniopterus magnater | KY370053 Alphacoronavirus Soracovirus China 2014 Sorex araneus musarania | 61.0% |
| NC046964 Alphacoronavirus Nyctacovirus Italy 2015 Pipistrellus kuhlii | KY370053 Alphacoronavirus Soracovirus China 2014 Sorex araneus musarania | 60.9% |
| NC028752 Alphacoronavirus Duvinacovirus Saudi Arabia 2015 Camel | KY370053 Alphacoronavirus Soracovirus China 2014 Sorex araneus musarania | 60.9% |
| NC028814 Alphacoronavirus Decacovirus China 2013 Rhinolophus ferrumequinum HuB2013 | KY370053 Alphacoronavirus Soracovirus China 2014 Sorex araneus musarania | 60.9% |
| NC048216 Alphacoronavirus Setracovirus Kenya 2010 Triaenops afer bat NL63 related bat coronavirus BtKYNL63 9b | KY370053 Alphacoronavirus Soracovirus China 2014 Sorex araneus musarania | 60.8% |
| KY370053 Alphacoronavirus Soracovirus China 2014 Sorex araneus musarania | KY967715 Alphacoronavirus Sunacovirus China 2015 Suncus murinus musarania | 60.5% |
| NC003436 Alphacoronavirus Pedacovirus Porcine | KY370053 Alphacoronavirus Soracovirus China 2014 Sorex araneus musarania | 60.5% |
| NC022103 Alphacoronavirus Colacovirus U.S. 2006 Myotis lucifugus | KY370053 Alphacoronavirus Soracovirus China 2014 Sorex araneus musarania | 60.5% |
| **OP700657 Tadarida brasilensis bat alphacoronavirus 2 isolate Tb3** | **KY370053 Alphacoronavirus Soracovirus China 2014 Sorex araneus musarania** | **60.4%** |
| **OP715780 Tadarida brasilensis bat alphacoronavirus 2 isolate Tb2** | **KY370053 Alphacoronavirus Soracovirus China 2014 Sorex araneus musarania** | **60.4%** |
| NC010438 Alphacoronavirus Minunacovirus Hong Kong 2004 Miniopterus bat HKU8 | KY370053 Alphacoronavirus Soracovirus China 2014 Sorex araneus musarania | 60.3% |
| NC009657 Alphacoronavirus Pedacovirus China 2005 Scotophilus bat | KY370053 Alphacoronavirus Soracovirus China 2014 Sorex araneus musarania | 60.3% |
| NC018871 Alphacoronavirus Decacovirus China 2005 Rousettus bat HKU10 | KY370053 Alphacoronavirus Soracovirus China 2014 Sorex araneus musarania | 60.3% |
| NC032107 Alphacoronavirus Setracovirus Kenia 2010 Triaenops afer NL63 related bat coronavirus BtKYNL63 9a | KY370053 Alphacoronavirus Soracovirus China 2014 Sorex araneus musarania | 60.3% |
| NC005831 Alphacoronavirus Setracovirus Netherlands 2002 Human NL63 | KY370053 Alphacoronavirus Soracovirus China 2014 Sorex araneus musarania | 60.2% |
| NC034972 Alphacoronavirus Luchacovirus China 2011 Apodemus chevrieri rat AcCoV JC34 | KY370053 Alphacoronavirus Soracovirus China 2014 Sorex araneus musarania | 60.1% |
| MZ293744 Alphacoronavirus Brazil 2014 Gardnerycteris crenulatum bat | KY370053 Alphacoronavirus Soracovirus China 2014 Sorex araneus musarania | 60.1% |
| NC032730 Alphacoronavirus Luchacovirus China 2013 Rattus norvegicus | KY370053 Alphacoronavirus Soracovirus China 2014 Sorex araneus musarania | 60.0% |
| MT663548 Alphacoronavirus Peru 2015 Desmodus rotundus | KY370053 Alphacoronavirus Soracovirus China 2014 Sorex araneus musarania | 59.9% |
| MW249018 Coronavirinae Peru 2016 Desmodus rotundus | KY370053 Alphacoronavirus Soracovirus China 2014 Sorex araneus musarania | 59.9% |
| OL410607 Alphacoronavirus U.S. 2020 Eptesicus fuscus | KY370053 Alphacoronavirus Soracovirus China 2014 Sorex araneus musarania | 59.8% |
| OL410609 Alphacoronavirus U.S. 2020 Eptesicus fuscus | KY370053 Alphacoronavirus Soracovirus China 2014 Sorex araneus musarania | 59.8% |
| MW924112 Alphacoronavirus Korea 2020 Eptesicus serotinus HCQD2020 | KY370053 Alphacoronavirus Soracovirus China 2014 Sorex araneus musarania | 59.8% |
| OL415262 Alphacoronavirus U.S. 2021 Eptesicus fuscus | KY370053 Alphacoronavirus Soracovirus China 2014 Sorex araneus musarania | 59.8% |
| **OP715781 Tadarida brasiliensis bat alphacoronavirus 1 isolate Tb1** | **KY370053 Alphacoronavirus Soracovirus China 2014 Sorex araneus musarania** | **59.5%** |
| NC010438 Alphacoronavirus Minunacovirus Hong Kong 2004 Miniopterus bat HKU8 | NC019843 Betacoronavirus Merbecovirus Saudi Arabia 2012 Human MERSCoV | 59.2% |
| NC019843 Betacoronavirus Merbecovirus Saudi Arabia 2012 Human MERSCoV | NC038861 Alphacoronavirus Tegacovirus U.S. Pig Transmissible gastroenteritis virus | 59.0% |
| NC009657 Alphacoronavirus Pedacovirus China 2005 Scotophilus bat | NC019843 Betacoronavirus Merbecovirus Saudi Arabia 2012 Human MERSCoV | 58.9% |
| NC019843 Betacoronavirus Merbecovirus Saudi Arabia 2012 Human MERSCoV | NC022103 Alphacoronavirus Colacovirus U.S. 2006 Myotis lucifugus | 58.9% |
| NC019843 Betacoronavirus Merbecovirus Saudi Arabia 2012 Human MERSCoV | NC028806 Alphacoronavirus Tegacovirus Italy 2009 Swine | 58.9% |
| **NC019843 Betacoronavirus Merbecovirus Saudi Arabia 2012 Human MERSCoV** | **OP700657 Tadarida brasilensis bat alphacoronavirus 2 isolate Tb3** | **58.9%** |
| **NC019843 Betacoronavirus Merbecovirus Saudi Arabia 2012 Human MERSCoV** | **OP715780 Tadarida brasilensis bat alphacoronavirus 2 isolate Tb2** | **58.9%** |
| NC002306 Alphacoronavirus Tegacovirus U.S. Feline | NC019843 Betacoronavirus Merbecovirus Saudi Arabia 2012 Human MERSCoV | 58.8% |
| NC019843 Betacoronavirus Merbecovirus Saudi Arabia 2012 Human MERSCoV | NC048216 Alphacoronavirus Setracovirus Kenya 2010 Triaenops afer bat NL63 related bat coronavirus BtKYNL63 9b | 58.7% |
| MN996532 Betacoronavirus Sarbecovirus China 2013 Rhinolophus affinis RaTG13 | NC023760 Alphacoronavirus Minacovirus U.S. 1998 Mustela vison | 58.6% |
| NC023760 Alphacoronavirus Minacovirus U.S. 1998 Mustela vison | NC045512 Betacoronavirus Sarbecovirus China 2019 Human SARS CoV 2 | 58.6% |
| **NC045512 Betacoronavirus Sarbecovirus China 2019 Human SARS CoV 2** | **OP700657 Tadarida brasilensis bat alphacoronavirus 2 isolate Tb3** | **58.6%** |
| **NC045512 Betacoronavirus Sarbecovirus China 2019 Human SARS CoV 2** | **OP715780 Tadarida brasilensis bat alphacoronavirus 2 isolate Tb2** | **58.6%** |
| NC003436 Alphacoronavirus Pedacovirus Porcine | NC019843 Betacoronavirus Merbecovirus Saudi Arabia 2012 Human MERSCoV | 58.6% |
| NC019843 Betacoronavirus Merbecovirus Saudi Arabia 2012 Human MERSCoV | NC023760 Alphacoronavirus Minacovirus U.S. 1998 Mustela vison | 58.6% |
| NC019843 Betacoronavirus Merbecovirus Saudi Arabia 2012 Human MERSCoV | NC028814 Alphacoronavirus Decacovirus China 2013 Rhinolophus ferrumequinum HuB2013 | 58.5% |
| NC019843 Betacoronavirus Merbecovirus Saudi Arabia 2012 Human MERSCoV | NC032107 Alphacoronavirus Setracovirus Kenia 2010 Triaenops afer NL63 related bat coronavirus BtKYNL63 9a | 58.5% |
| **MN996532 Betacoronavirus Sarbecovirus China 2013 Rhinolophus affinis RaTG13** | **OP700657 Tadarida brasilensis bat alphacoronavirus 2 isolate Tb3** | **58.5%** |
| **MN996532 Betacoronavirus Sarbecovirus China 2013 Rhinolophus affinis RaTG13** | **OP715780 Tadarida brasilensis bat alphacoronavirus 2 isolate Tb2** | **58.5%** |
| NC019843 Betacoronavirus Merbecovirus Saudi Arabia 2012 Human MERSCoV | NC030292 Alphacoronavirus Minacovirus Netherlands 2010 Mustela putorius ferret | 58.5% |
| MN996532 Betacoronavirus Sarbecovirus China 2013 Rhinolophus affinis RaTG13 | NC009657 Alphacoronavirus Pedacovirus China 2005 Scotophilus bat | 58.4% |
| NC009988 Alphacoronavirus Rhinacovirus China 2006 Rhinolophus bat HKU2 | NC019843 Betacoronavirus Merbecovirus Saudi Arabia 2012 Human MERSCoV | 58.4% |
| NC009657 Alphacoronavirus Pedacovirus China 2005 Scotophilus bat | NC045512 Betacoronavirus Sarbecovirus China 2019 Human SARS CoV 2 | 58.3% |
| MN996532 Betacoronavirus Sarbecovirus China 2013 Rhinolophus affinis RaTG13 | NC003436 Alphacoronavirus Pedacovirus Porcine | 58.3% |
| NC003436 Alphacoronavirus Pedacovirus Porcine | NC045512 Betacoronavirus Sarbecovirus China 2019 Human SARS CoV 2 | 58.3% |
| NC019843 Betacoronavirus Merbecovirus Saudi Arabia 2012 Human MERSCoV | NC028824 Alphacoronavirus Rhinacovirus China 2013 Rhinolophus ferrumequinum YN2012 | 58.3% |
| NC005831 Alphacoronavirus Setracovirus Netherlands 2002 Human NL63 | NC019843 Betacoronavirus Merbecovirus Saudi Arabia 2012 Human MERSCoV | 58.2% |
| NC010437 Alphacoronavirus Minunacovirus Hong Kong 2004 Miniopterus magnater | NC019843 Betacoronavirus Merbecovirus Saudi Arabia 2012 Human MERSCoV | 58.2% |
| NC028824 Alphacoronavirus Rhinacovirus China 2013 Rhinolophus ferrumequinum YN2012 | NC045512 Betacoronavirus Sarbecovirus China 2019 Human SARS CoV 2 | 58.2% |
| NC045512 Betacoronavirus Sarbecovirus China 2019 Human SARS CoV 2 | NC046964 Alphacoronavirus Nyctacovirus Italy 2015 Pipistrellus kuhlii | 58.1% |
| NC018871 Alphacoronavirus Decacovirus China 2005 Rousettus bat HKU10 | NC019843 Betacoronavirus Merbecovirus Saudi Arabia 2012 Human MERSCoV | 58.1% |
| NC019843 Betacoronavirus Merbecovirus Saudi Arabia 2012 Human MERSCoV | NC028833 Alphacoronavirus Nyctacovirus China 2013 Nyctalus velutinus bat | 58.1% |
| MN996532 Betacoronavirus Sarbecovirus China 2013 Rhinolophus affinis RaTG13 | NC046964 Alphacoronavirus Nyctacovirus Italy 2015 Pipistrellus kuhlii | 58.1% |
| NC019843 Betacoronavirus Merbecovirus Saudi Arabia 2012 Human MERSCoV | NC028752 Alphacoronavirus Duvinacovirus Saudi Arabia 2015 Camel | 58.0% |
| MN996532 Betacoronavirus Sarbecovirus China 2013 Rhinolophus affinis RaTG13 | NC030292 Alphacoronavirus Minacovirus Netherlands 2010 Mustela putorius ferret | 58.0% |
| NC030292 Alphacoronavirus Minacovirus Netherlands 2010 Mustela putorius ferret | NC045512 Betacoronavirus Sarbecovirus China 2019 Human SARS CoV 2 | 58.0% |
| MN996532 Betacoronavirus Sarbecovirus China 2013 Rhinolophus affinis RaTG13 | NC028824 Alphacoronavirus Rhinacovirus China 2013 Rhinolophus ferrumequinum YN2012 | 58.0% |
| NC010438 Alphacoronavirus Minunacovirus Hong Kong 2004 Miniopterus bat HKU8 | NC045512 Betacoronavirus Sarbecovirus China 2019 Human SARS CoV 2 | 58.0% |
| NC045512 Betacoronavirus Sarbecovirus China 2019 Human SARS CoV 2 | NC048216 Alphacoronavirus Setracovirus Kenya 2010 Triaenops afer bat NL63 related bat coronavirus BtKYNL63 9b | 58.0% |
| NC002645 Alphacoronavirus Duvinacovirus Human coronavirus 229E | NC019843 Betacoronavirus Merbecovirus Saudi Arabia 2012 Human MERSCoV | 58.0% |
| NC038861 Alphacoronavirus Tegacovirus U.S. Pig Transmissible gastroenteritis virus | NC045512 Betacoronavirus Sarbecovirus China 2019 Human SARS CoV 2 | 58.0% |
| NC009988 Alphacoronavirus Rhinacovirus China 2006 Rhinolophus bat HKU2 | NC045512 Betacoronavirus Sarbecovirus China 2019 Human SARS CoV 2 | 57.9% |
| NC010437 Alphacoronavirus Minunacovirus Hong Kong 2004 Miniopterus magnater | NC045512 Betacoronavirus Sarbecovirus China 2019 Human SARS CoV 2 | 57.9% |
| NC028814 Alphacoronavirus Decacovirus China 2013 Rhinolophus ferrumequinum HuB2013 | NC045512 Betacoronavirus Sarbecovirus China 2019 Human SARS CoV 2 | 57.9% |
| MN996532 Betacoronavirus Sarbecovirus China 2013 Rhinolophus affinis RaTG13 | NC048216 Alphacoronavirus Setracovirus Kenya 2010 Triaenops afer bat NL63 related bat coronavirus BtKYNL63 9b | 57.9% |
| MN996532 Betacoronavirus Sarbecovirus China 2013 Rhinolophus affinis RaTG13 | NC010438 Alphacoronavirus Minunacovirus Hong Kong 2004 Miniopterus bat HKU8 | 57.9% |
| MN996532 Betacoronavirus Sarbecovirus China 2013 Rhinolophus affinis RaTG13 | NC028814 Alphacoronavirus Decacovirus China 2013 Rhinolophus ferrumequinum HuB2013 | 57.9% |
| NC018871 Alphacoronavirus Decacovirus China 2005 Rousettus bat HKU10 | NC045512 Betacoronavirus Sarbecovirus China 2019 Human SARS CoV 2 | 57.9% |
| NC028833 Alphacoronavirus Nyctacovirus China 2013 Nyctalus velutinus bat | NC045512 Betacoronavirus Sarbecovirus China 2019 Human SARS CoV 2 | 57.9% |
| NC019843 Betacoronavirus Merbecovirus Saudi Arabia 2012 Human MERSCoV | MZ293744 Alphacoronavirus Brazil 2014 Gardnerycteris crenulatum bat | 57.9% |
| MN996532 Betacoronavirus Sarbecovirus China 2013 Rhinolophus affinis RaTG13 | NC038861 Alphacoronavirus Tegacovirus U.S. Pig Transmissible gastroenteritis virus | 57.9% |
| NC002306 Alphacoronavirus Tegacovirus U.S. Feline | NC045512 Betacoronavirus Sarbecovirus China 2019 Human SARS CoV 2 | 57.9% |
| MN996532 Betacoronavirus Sarbecovirus China 2013 Rhinolophus affinis RaTG13 | NC018871 Alphacoronavirus Decacovirus China 2005 Rousettus bat HKU10 | 57.8% |
| MN996532 Betacoronavirus Sarbecovirus China 2013 Rhinolophus affinis RaTG13 | NC028833 Alphacoronavirus Nyctacovirus China 2013 Nyctalus velutinus bat | 57.8% |
| MZ328299 Alphacoronavirus China 2016 Miniopterus schreibersii | NC045512 Betacoronavirus Sarbecovirus China 2019 Human SARS CoV 2 | 57.8% |
| NC019843 Betacoronavirus Merbecovirus Saudi Arabia 2012 Human MERSCoV | NC034972 Alphacoronavirus Luchacovirus China 2011 Apodemus chevrieri rat AcCoV JC34 | 57.8% |
| NC028806 Alphacoronavirus Tegacovirus Italy 2009 Swine | NC045512 Betacoronavirus Sarbecovirus China 2019 Human SARS CoV 2 | 57.8% |
| MN996532 Betacoronavirus Sarbecovirus China 2013 Rhinolophus affinis RaTG13 | MZ328299 Alphacoronavirus China 2016 Miniopterus schreibersii | 57.8% |
| MN996532 Betacoronavirus Sarbecovirus China 2013 Rhinolophus affinis RaTG13 | NC009988 Alphacoronavirus Rhinacovirus China 2006 Rhinolophus bat HKU2 | 57.8% |
| MN996532 Betacoronavirus Sarbecovirus China 2013 Rhinolophus affinis RaTG13 | NC010437 Alphacoronavirus Minunacovirus Hong Kong 2004 Miniopterus magnater | 57.8% |
| MN996532 Betacoronavirus Sarbecovirus China 2013 Rhinolophus affinis RaTG13 | NC002306 Alphacoronavirus Tegacovirus U.S. Feline | 57.7% |
| MZ081397 Alphacoronavirus China 2020 Myotis laniger | NC019843 Betacoronavirus Merbecovirus Saudi Arabia 2012 Human MERSCoV | 57.7% |
| MN996532 Betacoronavirus Sarbecovirus China 2013 Rhinolophus affinis RaTG13 | NC028806 Alphacoronavirus Tegacovirus Italy 2009 Swine | 57.7% |
| MW924112 Alphacoronavirus Korea 2020 Eptesicus serotinus HCQD2020 | NC045512 Betacoronavirus Sarbecovirus China 2019 Human SARS CoV 2 | 57.7% |
| NC019843 Betacoronavirus Merbecovirus Saudi Arabia 2012 Human MERSCoV | NC046964 Alphacoronavirus Nyctacovirus Italy 2015 Pipistrellus kuhlii | 57.7% |
| MN535734 Coronavirinae Denmark 2016 Myotis dasycneme | NC019843 Betacoronavirus Merbecovirus Saudi Arabia 2012 Human MERSCoV | 57.7% |
| NC019843 Betacoronavirus Merbecovirus Saudi Arabia 2012 Human MERSCoV | NC032730 Alphacoronavirus Luchacovirus China 2013 Rattus norvegicus | 57.6% |
| **NC045512 Betacoronavirus Sarbecovirus China 2019 Human SARS CoV 2** | **OP715781 Tadarida brasiliensis bat alphacoronavirus 1 isolate Tb1** | **57.6%** |
| MN996532 Betacoronavirus Sarbecovirus China 2013 Rhinolophus affinis RaTG13 | MW924112 Alphacoronavirus Korea 2020 Eptesicus serotinus HCQD2020 | 57.6% |
| MW249018 Coronavirinae Peru 2016 Desmodus rotundus | NC019843 Betacoronavirus Merbecovirus Saudi Arabia 2012 Human MERSCoV | 57.5% |
| MZ328299 Alphacoronavirus China 2016 Miniopterus schreibersii | NC019843 Betacoronavirus Merbecovirus Saudi Arabia 2012 Human MERSCoV | 57.5% |
| NC022103 Alphacoronavirus Colacovirus U.S. 2006 Myotis lucifugus | NC045512 Betacoronavirus Sarbecovirus China 2019 Human SARS CoV 2 | 57.5% |
| **MN996532 Betacoronavirus Sarbecovirus China 2013 Rhinolophus affinis RaTG13** | **OP715781 Tadarida brasiliensis bat alphacoronavirus 1 isolate Tb1** | **57.5%** |
| MT663548 Alphacoronavirus Peru 2015 Desmodus rotundus | NC019843 Betacoronavirus Merbecovirus Saudi Arabia 2012 Human MERSCoV | 57.5% |
| NC019843 Betacoronavirus Merbecovirus Saudi Arabia 2012 Human MERSCoV | NC028811 Alphacoronavirus Myotacovirus China 2011 Myotis ricketti SAX2011 | 57.5% |
| MN996532 Betacoronavirus Sarbecovirus China 2013 Rhinolophus affinis RaTG13 | NC022103 Alphacoronavirus Colacovirus U.S. 2006 Myotis lucifugus | 57.5% |
| NC045512 Betacoronavirus Sarbecovirus China 2019 Human SARS CoV 2 | OL410607 Alphacoronavirus U.S. 2020 Eptesicus fuscus | 57.5% |
| NC045512 Betacoronavirus Sarbecovirus China 2019 Human SARS CoV 2 | OL415262 Alphacoronavirus U.S. 2021 Eptesicus fuscus | 57.5% |
| MZ328298 Alphacoronavirus China 2016 Myotis chinensis | NC019843 Betacoronavirus Merbecovirus Saudi Arabia 2012 Human MERSCoV | 57.4% |
| MN535734 Coronavirinae Denmark 2016 Myotis dasycneme | NC045512 Betacoronavirus Sarbecovirus China 2019 Human SARS CoV 2 | 57.4% |
| NC032107 Alphacoronavirus Setracovirus Kenia 2010 Triaenops afer NL63 related bat coronavirus BtKYNL63 9a | NC045512 Betacoronavirus Sarbecovirus China 2019 Human SARS CoV 2 | 57.4% |
| MN996532 Betacoronavirus Sarbecovirus China 2013 Rhinolophus affinis RaTG13 | NC032107 Alphacoronavirus Setracovirus Kenia 2010 Triaenops afer NL63 related bat coronavirus BtKYNL63 9a | 57.3% |
| MN996532 Betacoronavirus Sarbecovirus China 2013 Rhinolophus affinis RaTG13 | OL410607 Alphacoronavirus U.S. 2020 Eptesicus fuscus | 57.3% |
| NC045512 Betacoronavirus Sarbecovirus China 2019 Human SARS CoV 2 | OL410609 Alphacoronavirus U.S. 2020 Eptesicus fuscus | 57.3% |
| MN996532 Betacoronavirus Sarbecovirus China 2013 Rhinolophus affinis RaTG13 | OL415262 Alphacoronavirus U.S. 2021 Eptesicus fuscus | 57.3% |
| MN535734 Coronavirinae Denmark 2016 Myotis dasycneme | MN996532 Betacoronavirus Sarbecovirus China 2013 Rhinolophus affinis RaTG13 | 57.3% |
| MZ081397 Alphacoronavirus China 2020 Myotis laniger | NC045512 Betacoronavirus Sarbecovirus China 2019 Human SARS CoV 2 | 57.3% |
| MN996532 Betacoronavirus Sarbecovirus China 2013 Rhinolophus affinis RaTG13 | MZ293744 Alphacoronavirus Brazil 2014 Gardnerycteris crenulatum bat | 57.3% |
| MN996532 Betacoronavirus Sarbecovirus China 2013 Rhinolophus affinis RaTG13 | MZ081397 Alphacoronavirus China 2020 Myotis laniger | 57.3% |
| **NC019843 Betacoronavirus Merbecovirus Saudi Arabia 2012 Human MERSCoV** | **OP715781 Tadarida brasiliensis bat alphacoronavirus 1 isolate Tb1** | **57.3%** |
| NC045512 Betacoronavirus Sarbecovirus China 2019 Human SARS CoV 2 | MZ293744 Alphacoronavirus Brazil 2014 Gardnerycteris crenulatum bat | 57.3% |
| MN996532 Betacoronavirus Sarbecovirus China 2013 Rhinolophus affinis RaTG13 | OL410609 Alphacoronavirus U.S. 2020 Eptesicus fuscus | 57.2% |
| NC019843 Betacoronavirus Merbecovirus Saudi Arabia 2012 Human MERSCoV | OL410609 Alphacoronavirus U.S. 2020 Eptesicus fuscus | 57.2% |
| NC019843 Betacoronavirus Merbecovirus Saudi Arabia 2012 Human MERSCoV | OL410607 Alphacoronavirus U.S. 2020 Eptesicus fuscus | 57.2% |
| NC019843 Betacoronavirus Merbecovirus Saudi Arabia 2012 Human MERSCoV | OL415262 Alphacoronavirus U.S. 2021 Eptesicus fuscus | 57.2% |
| MW924112 Alphacoronavirus Korea 2020 Eptesicus serotinus HCQD2020 | NC019843 Betacoronavirus Merbecovirus Saudi Arabia 2012 Human MERSCoV | 57.2% |
| NC005831 Alphacoronavirus Setracovirus Netherlands 2002 Human NL63 | NC045512 Betacoronavirus Sarbecovirus China 2019 Human SARS CoV 2 | 57.1% |
| MN996532 Betacoronavirus Sarbecovirus China 2013 Rhinolophus affinis RaTG13 | MZ328298 Alphacoronavirus China 2016 Myotis chinensis | 57.1% |
| MN996532 Betacoronavirus Sarbecovirus China 2013 Rhinolophus affinis RaTG13 | NC005831 Alphacoronavirus Setracovirus Netherlands 2002 Human NL63 | 57.1% |
| MN996532 Betacoronavirus Sarbecovirus China 2013 Rhinolophus affinis RaTG13 | NC028811 Alphacoronavirus Myotacovirus China 2011 Myotis ricketti SAX2011 | 57.1% |
| MZ328298 Alphacoronavirus China 2016 Myotis chinensis | NC045512 Betacoronavirus Sarbecovirus China 2019 Human SARS CoV 2 | 57.1% |
| NC028811 Alphacoronavirus Myotacovirus China 2011 Myotis ricketti SAX2011 | NC045512 Betacoronavirus Sarbecovirus China 2019 Human SARS CoV 2 | 57.1% |
| NC034972 Alphacoronavirus Luchacovirus China 2011 Apodemus chevrieri rat AcCoV JC34 | NC045512 Betacoronavirus Sarbecovirus China 2019 Human SARS CoV 2 | 57.0% |
| MN996532 Betacoronavirus Sarbecovirus China 2013 Rhinolophus affinis RaTG13 | MT663548 Alphacoronavirus Peru 2015 Desmodus rotundus | 56.9% |
| MN996532 Betacoronavirus Sarbecovirus China 2013 Rhinolophus affinis RaTG13 | MW249018 Coronavirinae Peru 2016 Desmodus rotundus | 56.9% |
| MN996532 Betacoronavirus Sarbecovirus China 2013 Rhinolophus affinis RaTG13 | NC034972 Alphacoronavirus Luchacovirus China 2011 Apodemus chevrieri rat AcCoV JC34 | 56.9% |
| MT663548 Alphacoronavirus Peru 2015 Desmodus rotundus | NC045512 Betacoronavirus Sarbecovirus China 2019 Human SARS CoV 2 | 56.9% |
| MW249018 Coronavirinae Peru 2016 Desmodus rotundus | NC045512 Betacoronavirus Sarbecovirus China 2019 Human SARS CoV 2 | 56.9% |
| NC019843 Betacoronavirus Merbecovirus Saudi Arabia 2012 Human MERSCoV | KY370053 Alphacoronavirus Soracovirus China 2014 Sorex araneus musarania | 56.7% |
| MN996532 Betacoronavirus Sarbecovirus China 2013 Rhinolophus affinis RaTG13 | NC028752 Alphacoronavirus Duvinacovirus Saudi Arabia 2015 Camel | 56.7% |
| NC028752 Alphacoronavirus Duvinacovirus Saudi Arabia 2015 Camel | NC045512 Betacoronavirus Sarbecovirus China 2019 Human SARS CoV 2 | 56.7% |
| NC019843 Betacoronavirus Merbecovirus Saudi Arabia 2012 Human MERSCoV | KY967715 Alphacoronavirus Sunacovirus China 2015 Suncus murinus musarania | 56.7% |
| NC032730 Alphacoronavirus Luchacovirus China 2013 Rattus norvegicus | NC045512 Betacoronavirus Sarbecovirus China 2019 Human SARS CoV 2 | 56.6% |
| NC006577 Betacoronavirus Embecovirus China 2004 Human HKU1 | NC028806 Alphacoronavirus Tegacovirus Italy 2009 Swine | 56.6% |
| NC006577 Betacoronavirus Embecovirus China 2004 Human HKU1 | NC038861 Alphacoronavirus Tegacovirus U.S. Pig Transmissible gastroenteritis virus | 56.6% |
| MN996532 Betacoronavirus Sarbecovirus China 2013 Rhinolophus affinis RaTG13 | NC032730 Alphacoronavirus Luchacovirus China 2013 Rattus norvegicus | 56.5% |
| MN996532 Betacoronavirus Sarbecovirus China 2013 Rhinolophus affinis RaTG13 | NC002645 Alphacoronavirus Duvinacovirus Human coronavirus 229E | 56.5% |
| NC002645 Alphacoronavirus Duvinacovirus Human coronavirus 229E | NC045512 Betacoronavirus Sarbecovirus China 2019 Human SARS CoV 2 | 56.5% |
| NC006577 Betacoronavirus Embecovirus China 2004 Human HKU1 | NC030292 Alphacoronavirus Minacovirus Netherlands 2010 Mustela putorius ferret | 56.2% |
| NC006577 Betacoronavirus Embecovirus China 2004 Human HKU1 | MZ293744 Alphacoronavirus Brazil 2014 Gardnerycteris crenulatum bat | 56.2% |
| NC002306 Alphacoronavirus Tegacovirus U.S. Feline | NC006577 Betacoronavirus Embecovirus China 2004 Human HKU1 | 56.1% |
| NC045512 Betacoronavirus Sarbecovirus China 2019 Human SARS CoV 2 | KY370053 Alphacoronavirus Soracovirus China 2014 Sorex araneus musarania | 56.1% |
| MN996532 Betacoronavirus Sarbecovirus China 2013 Rhinolophus affinis RaTG13 | KY370053 Alphacoronavirus Soracovirus China 2014 Sorex araneus musarania | 55.9% |
| MT663548 Alphacoronavirus Peru 2015 Desmodus rotundus | NC006577 Betacoronavirus Embecovirus China 2004 Human HKU1 | 55.9% |
| NC006577 Betacoronavirus Embecovirus China 2004 Human HKU1 | NC028833 Alphacoronavirus Nyctacovirus China 2013 Nyctalus velutinus bat | 55.9% |
| MW249018 Coronavirinae Peru 2016 Desmodus rotundus | NC006577 Betacoronavirus Embecovirus China 2004 Human HKU1 | 55.7% |
| NC003436 Alphacoronavirus Pedacovirus Porcine | NC006577 Betacoronavirus Embecovirus China 2004 Human HKU1 | 55.7% |
| NC006577 Betacoronavirus Embecovirus China 2004 Human HKU1 | NC023760 Alphacoronavirus Minacovirus U.S. 1998 Mustela vison | 55.6% |
| NC006577 Betacoronavirus Embecovirus China 2004 Human HKU1 | NC048216 Alphacoronavirus Setracovirus Kenya 2010 Triaenops afer bat NL63 related bat coronavirus BtKYNL63 9b | 55.4% |
| NC006577 Betacoronavirus Embecovirus China 2004 Human HKU1 | NC010437 Alphacoronavirus Minunacovirus Hong Kong 2004 Miniopterus magnater | 55.4% |
| NC045512 Betacoronavirus Sarbecovirus China 2019 Human SARS CoV 2 | KY967715 Alphacoronavirus Sunacovirus China 2015 Suncus murinus musarania | 55.4% |
| NC006577 Betacoronavirus Embecovirus China 2004 Human HKU1 | NC034972 Alphacoronavirus Luchacovirus China 2011 Apodemus chevrieri rat AcCoV JC34 | 55.3% |
| NC006577 Betacoronavirus Embecovirus China 2004 Human HKU1 | NC022103 Alphacoronavirus Colacovirus U.S. 2006 Myotis lucifugus | 55.3% |
| MN996532 Betacoronavirus Sarbecovirus China 2013 Rhinolophus affinis RaTG13 | KY967715 Alphacoronavirus Sunacovirus China 2015 Suncus murinus musarania | 55.2% |
| NC006577 Betacoronavirus Embecovirus China 2004 Human HKU1 | KY967715 Alphacoronavirus Sunacovirus China 2015 Suncus murinus musarania | 55.1% |
| **NC006577 Betacoronavirus Embecovirus China 2004 Human HKU1** | **OP700657 Tadarida brasilensis bat alphacoronavirus 2 isolate Tb3** | **55.0%** |
| **NC006577 Betacoronavirus Embecovirus China 2004 Human HKU1** | **OP715780 Tadarida brasilensis bat alphacoronavirus 2 isolate Tb2** | **55.0%** |
| NC006577 Betacoronavirus Embecovirus China 2004 Human HKU1 | NC028752 Alphacoronavirus Duvinacovirus Saudi Arabia 2015 Camel | 54.9% |
| NC006577 Betacoronavirus Embecovirus China 2004 Human HKU1 | NC032730 Alphacoronavirus Luchacovirus China 2013 Rattus norvegicus | 54.8% |
| NC006577 Betacoronavirus Embecovirus China 2004 Human HKU1 | NC046964 Alphacoronavirus Nyctacovirus Italy 2015 Pipistrellus kuhlii | 54.8% |
| NC006577 Betacoronavirus Embecovirus China 2004 Human HKU1 | KY370053 Alphacoronavirus Soracovirus China 2014 Sorex araneus musarania | 54.8% |
| MW924112 Alphacoronavirus Korea 2020 Eptesicus serotinus HCQD2020 | NC006577 Betacoronavirus Embecovirus China 2004 Human HKU1 | 54.7% |
| NC006577 Betacoronavirus Embecovirus China 2004 Human HKU1 | OL410609 Alphacoronavirus U.S. 2020 Eptesicus fuscus | 54.6% |
| MZ081397 Alphacoronavirus China 2020 Myotis laniger | NC006577 Betacoronavirus Embecovirus China 2004 Human HKU1 | 54.6% |
| NC006577 Betacoronavirus Embecovirus China 2004 Human HKU1 | NC009657 Alphacoronavirus Pedacovirus China 2005 Scotophilus bat | 54.6% |
| NC006577 Betacoronavirus Embecovirus China 2004 Human HKU1 | NC010438 Alphacoronavirus Minunacovirus Hong Kong 2004 Miniopterus bat HKU8 | 54.6% |
| NC006577 Betacoronavirus Embecovirus China 2004 Human HKU1 | OL415262 Alphacoronavirus U.S. 2021 Eptesicus fuscus | 54.6% |
| **NC006577 Betacoronavirus Embecovirus China 2004 Human HKU1** | **OP715781 Tadarida brasiliensis bat alphacoronavirus 1 isolate Tb1** | **54.6%** |
| NC002645 Alphacoronavirus Duvinacovirus Human coronavirus 229E | NC006577 Betacoronavirus Embecovirus China 2004 Human HKU1 | 54.5% |
| NC006577 Betacoronavirus Embecovirus China 2004 Human HKU1 | OL410607 Alphacoronavirus U.S. 2020 Eptesicus fuscus | 54.5% |
| MZ328299 Alphacoronavirus China 2016 Miniopterus schreibersii | NC006577 Betacoronavirus Embecovirus China 2004 Human HKU1 | 54.5% |
| NC006577 Betacoronavirus Embecovirus China 2004 Human HKU1 | NC018871 Alphacoronavirus Decacovirus China 2005 Rousettus bat HKU10 | 54.4% |
| NC005831 Alphacoronavirus Setracovirus Netherlands 2002 Human NL63 | NC006577 Betacoronavirus Embecovirus China 2004 Human HKU1 | 54.3% |
| NC006577 Betacoronavirus Embecovirus China 2004 Human HKU1 | NC028814 Alphacoronavirus Decacovirus China 2013 Rhinolophus ferrumequinum HuB2013 | 54.3% |
| MZ328298 Alphacoronavirus China 2016 Myotis chinensis | NC006577 Betacoronavirus Embecovirus China 2004 Human HKU1 | 54.2% |
| NC006577 Betacoronavirus Embecovirus China 2004 Human HKU1 | NC028811 Alphacoronavirus Myotacovirus China 2011 Myotis ricketti SAX2011 | 54.2% |
| MN535734 Coronavirinae Denmark 2016 Myotis dasycneme | NC006577 Betacoronavirus Embecovirus China 2004 Human HKU1 | 54.2% |
| NC006577 Betacoronavirus Embecovirus China 2004 Human HKU1 | NC032107 Alphacoronavirus Setracovirus Kenia 2010 Triaenops afer NL63 related bat coronavirus BtKYNL63 9a | 54.2% |
| NC006577 Betacoronavirus Embecovirus China 2004 Human HKU1 | NC009988 Alphacoronavirus Rhinacovirus China 2006 Rhinolophus bat HKU2 | 54.1% |
| NC006577 Betacoronavirus Embecovirus China 2004 Human HKU1 | NC028824 Alphacoronavirus Rhinacovirus China 2013 Rhinolophus ferrumequinum YN2012 | 54.1% |

Comparisons involving novel AlphaCoV genomes identified in this work are depicted in bold.
