# Supplementary material for: Identification and characterization of novel alphacoronaviruses in Tadarida brasiliensis (Chiroptera, Molossidae) from Argentina: insights into recombination as a mechanism favoring bat coronavirus cross-species transmission
Source: Microbiol Spectr. 2023 Sep 11;11(5):e02047-23. doi: 10.1128/spectrum.02047-23 (PMC10581097; doi:10.1128/spectrum.02047-23)
Supplement: Table S5 — Recombination events meeting the inclusion criterion. [file spectrum.02047-23-s0007.docx]

**SUPPLEMENTARY TABLE S5** Recombination events meeting the inclusion criterion.

| **Event number** | **Putative recombinant virus** | **Species Breakpoint localization relative to recombinant virus (nt position)** | **Genes in recombinant region** | **Recombination length (nt)** | **Parent sequences** | | **Method (p-value)** | | | | | | |
| --- | --- | --- | --- | --- | --- | --- | --- | --- | --- | --- | --- | --- | --- |
|  |  |  |  |  | **Minor** | **Major** | **RDP** | **GENECONV** | **Bootscan** | **Maxchi** | **Chimaera** | **SiScan** | **3Seq** |
| 1 | KJ473798 | 21,076–26,162 | S, ORF3, E, M | 5,086 | Unknown (MN611518) | KJ473800 | 6.59 × 10^−318^ | 9.77 × 10^−301^ | 2.76 × 10^−279^ | 1.23 × 10^−69^ | 5.99 × 10^−13^ | 1.07 × 10^−75^ | 2.96 × 10^−10^ |
|  |  |  |  |  |  | KJ473799 |  |  |  |  |  |  |  |
| 2 | MK211370 | 20,573–25,588* | ORF1ab, S, E | 5,015 | MH687939 | MH687966 | 5.39 × 10^−111^ | 3.40 × 10^−291^ | 1.07 × 10^−99^ | 3.25 × 10^−75^ | 9.16 × 10^−3^ | 2.08 × 10^−79^ | 4.89 × 10^−10^ |
|  | MK211369 |  |  |  | MH687935 | MH687957 |  |  |  |  |  |  |  |
|  | MK211371 |  |  |  |  | MN611521 |  |  |  |  |  |  |  |
|  | DQ648858 |  |  |  |  |  |  |  |  |  |  |  |  |
|  | MH687954 |  |  |  |  |  |  |  |  |  |  |  |  |
|  | MH687964 |  |  |  |  |  |  |  |  |  |  |  |  |
|  | MH687941 |  |  |  |  |  |  |  |  |  |  |  |  |
| 3 | EU420139 | 1*–21,047 | ORF1ab | 21,046 | KJ473797 | Unknown (KJ473799) | 1.91 × 10^−316^ | 5.21 × 10^−302^ | NS | 4.39 × 10^−94^ | 1.52 × 10^−7^ | 1.03 × 10^−160^ | 1.18 × 10^−10^ |
|  |  |  |  |  | MN611518 |  |  |  |  |  |  |  |  |
| 4 | NC028806 | 21,275–24,674 | S, ORF3 | 3,399 | Unknown (NC002306) | AJ271965 | NS | 2.67 × 10^−295^ | NS | 6.05 × 10^−44^ | 7.95 × 10^−6^ | 1.20 × 10^−97^ | 2.96 × 10^−10^ |
| 5 | OK287352 | 20,492–24,731* | ORF1b, S, ORF3 | 4,239 | Unknown (JQ989266) | MZ081390 | 1.77 × 10^−259^ | NS | 4.08 × 10^−225^ | 5.49 × 10^−3^ | 8.10 × 10^−67^ | 4.73 × 10^−63^ | 2.67 × 10^−10^ |
|  |  |  |  |  | Unknown (JQ989272) |  |  |  |  |  |  |  |  |
|  |  |  |  |  | Unknown (MN611523) |  |  |  |  |  |  |  |  |
| 6 | JQ989271 | 20,657–24,562* | S | 3,905 | Unknown (MZ081390) | JQ989266 | 4.22 × 10^−252^ | 1.41 × 10^−194^ | 9.70 × 10^−226^ | 4.30 × 10^−39^ | 8.29 × 10^−63^ | 5.12 × 10^−65^ | 2.67 × 10^−10^ |
|  | JQ989270 |  |  |  |  |  |  |  |  |  |  |  |  |
| 7 | MH687957 | 25,840–27,423 | M, N | 1,583 | Unknown (MH687939) | MH687956 | 1.87 × 10^−118^ | 6.03 × 10^−45^ | 8.71 × 10^−109^ | 9.75 × 10^−21^ | 5.91 × 10^−10^ | 1.55 × 10^−17^ | 1.72 × 10^−7^ |
|  | MH687966 |  |  |  | Unknown (MH687935) | MK211369 |  |  |  |  |  |  |  |
|  | MN611521 |  |  |  |  | MK211371 |  |  |  |  |  |  |  |
|  |  |  |  |  |  | DQ648858 |  |  |  |  |  |  |  |
|  |  |  |  |  |  | MH687954 |  |  |  |  |  |  |  |
|  |  |  |  |  |  | MH687964 |  |  |  |  |  |  |  |
|  |  |  |  |  |  | MH687941 |  |  |  |  |  |  |  |
|  |  |  |  |  |  | MH687965 |  |  |  |  |  |  |  |
| 8 | MZ081391 | 20,542–22,661 | ORF1b, S | 2,119 | Unknown (JQ989266) | MZ081390 | 8.97 × 10^−137^ | 1.46 × 10^−54^ | 2.19 × 10^−125^ | 4.31 × 10^−28^ | 1.15 × 10^−31^ | 4.14 × 10^−23^ | 2.96 × 10^−10^ |
|  |  |  |  |  | Unknown (MN611523) |  |  |  |  |  |  |  |  |
| 9 | MK211373 | 20,267–23,159 | ORF1b, S | 2,892 | MZ081388 | MG916901 | 1.88 × 10^−126^ | 1.24 × 10^−91^ | 8.17 × 10^−127^ | 9.24 × 10^−17^ | NS | 2.88 × 10^−82^ | 2.96 × 10^−11^ |
| 10 | MZ081388 | 20,420–22,047* | ORF1b, S | 1,627 | MZ081387 | MG916901 | 3.22 × 10^−95^ | NS | 1.29 × 10^−48^ | 2.27 × 10^−45^ | 9.51 × 10^−17^ | 7.43 × 10^−4^ | 5.33 × 10^−10^ |
|  |  |  |  |  |  | OK287353 |  |  |  |  |  |  |  |
| 11 | AY567487 | 20,781–22,874 | S | 2,093 | MN611517 | Unknown (NC028752) | 9.64 × 10^−84^ | NS | 2.90 × 10^−27^ | 3.73 × 10^−17^ | 3.11 × 10^−22^ | 5.10 × 10^−4^ | 6.22 × 10^−7^ |
|  |  |  |  |  |  | Unknown (AF304460) |  |  |  |  |  |  |  |
| 12 | MH687935 | 7,853–16,337 | ORF1ab | 8,484 | MH687953 | MH687939 | 1.38 × 10^−74^ | 2.96 × 10^−80^ | 4.26 × 10^−73^ | 5.02 × 10^−31^ | 9.18 × 10^−32^ | 1.19 × 10^−32^ | 2.96 × 10^−11^ |
| 13 | KJ473808 | 8,020–10,353 | ORF1a | 2,333 | MN611522 | MZ081399 | 1.24 × 10^−77^ | 3.82 × 10^−20^ | 4.32 × 10^−74^ | 2.56 × 10^−16^ | 5.26 × 10^−18^ | 1.94 × 10^−21^ | 2.07 × 10^−10^ |
|  |  |  |  |  |  | MZ081398 |  |  |  |  |  |  |  |
| 14 | MG916902 | 21,148–25,165* | S, ORF3 | 4,017 | Unknown (MZ081384) | MG916901 | 4.45 × 10^−56^ | NS | 6.40 × 10^−15^ | 3.32 × 10^−24^ | 9.59 × 10^−29^ | 6.69 × 10^−20^ | 2.07 × 10^−10^ |
|  | MG916903 |  |  |  |  | OK287353 |  |  |  |  |  |  |  |
| 15 | MH687965 | 20,479–22,442 | ORF1b, S | 1,963 | Unknown (MK211372) | MH687956 | 8.44 × 10^−54^ | 6.20 × 10^−53^ | 3.95 × 10^−53^ | 3.60 × 10^−12^ | 1.96 × 10^−15^ | 1.07 × 10^−23^ | 8.89 × 10^−11^ |
|  |  |  |  |  |  | MH687957 |  |  |  |  |  |  |  |
| **16** | **OL410609** | **21,796–23,414** | **S** | **1,618** | **OP715781** | **OL410607** | **1.31 × 10^−51^** | **NS** | **1.92 × 10^−34^** | **1.44 × 10^−8^** | **1.24 × 10^−17^** | **2.54 x10^−3^** | **4.15 × 10^−10^** |
| 17 | MZ218052 | 15,030–18,582 | ORF1ab | 3,552 | MN535733 | MN535732 | 5.99 × 10^−37^ | 1.10 × 10^−62^ | 2.95 × 10^−45^ | 3.66 × 10^−15^ | 4.99 × 10^−17^ | 2.64 × 10^−18^ | 1.18 × 10^−10^ |
| 18 | MZ218052 | 24,908–27,425* | ORF3, E, M, N | 2,517 | MG923574 | MN535732 | 4.19 × 10^−40^ | NS | 1.44 × 10^−14^ | 6.13 × 10^−5^ | 2.98 × 10^−14^ | NS | 1.18 × 10^−10^ |
| 19 | MN611522 | 2,662–13,997 | ORF1ab | 11,335 | MT747186 | Unknown (MZ081399) | 6.09 × 10^−33^ | 1.40 × 10^−34^ | 2.91 × 10^−32^ | 6.87 × 10^−35^ | 1.99 × 10^−24^ | 2.68 × 10^−49^ | 5.93 × 10^−11^ |
|  |  |  |  |  | EF203064 | Unknown (MZ081398) |  |  |  |  |  |  |  |
|  |  |  |  |  | EF203065 | Unknown (KJ473808) |  |  |  |  |  |  |  |
| 20 | MH687966 | 4,544–10,489 | ORF1ab | 5,945 | MH687965 | Unknown (MH687957) | 2.21 × 10^−28^ | 4.94 × 10^−31^ | 8.44 × 10^−31^ | 1.76 × 10^−12^ | 1.09 × 10^−12^ | 1.37 × 10^−16^ | 2.96 × 10^−11^ |
| 21 | MH687964 | 10,423–19,311 | ORF1ab | 8,888 | MH687954 | MH687941 | 3.30 × 10^−8^ | 1.71 × 10^−24^ | 1.71 × 10^−12^ | 1.96 × 10^−8^ | 2.91 × 10^−13^ | 3.65 × 10^−28^ | 7.81 × 10^−16^ |
| 22 | MW924112 | 17,870–20,736 | ORF1ab | 2,866 | OL415262 | Unknown (OL410609) | 7.85 × 10^−13^ | 2.96 × 10^−24^ | 5.26 × 10^−21^ | 1.35 × 10^−9^ | 1.93 × 10^−12^ | 1.18 × 10^−29^ | NS |
| 23 | MZ081388 | 2,348*–19,870* | ORF1ab | 17,522 | MG916901 | Unknown (OK287353) | 8.67 × 10^−24^ | 8.38 × 10^−20^ | 8.71 × 10^−7^ | 1.05 × 10^−18^ | 3.75 × 10^−22^ | NS | 2.96 × 10^−11^ |
|  |  |  |  |  | MK211373 |  |  |  |  |  |  |  |  |
| 24 | MH687957 | 13,107–18,226 | ORF1ab | 5,119 | MH687966 | MH687965 | 2.81 × 10^−13^ | 2.44 × 10^−22^ | 4.75 × 10^−17^ | 6.57 × 10^−15^ | 1.10 × 10^−15^ | 1.99 × 10^−20^ | 1.24 × 10^−16^ |
| 25 | MH687966 | 10,636*–14,820 | ORF1ab | 4,184 | MH687956 | Unknown (MH687954) | NS | 8.77 × 10^−4^ | 1.28 × 10^−5^ | 7.74 × 10^−7^ | 3.31 × 10^−2^ | 3.62 × 10^−7^ | 7.46 × 10^−23^ |
|  |  |  |  |  | MH687941 |  |  |  |  |  |  |  |  |
| 26 | MH687965 | 704–13,149* | ORF1ab | 12,445 | MH687956 | Unknown (MH687957) | 3.91 × 10^−10^ | 1.95 × 10^−12^ | 2.33 × 10^−16^ | 1.64 × 10^−6^ | 3.35 × 10^−6^ | 1.21 × 10^−20^ | 6.34 × 10^−12^ |
|  | MH687941 |  |  |  |  |  |  |  |  |  |  |  |  |
| 27 | MG923574 | 10,978–12,929* | ORF1ab | 1,951 | MZ218052 | MN535733 | 5.94 × 10^−18^ | 3.97 × 10^−5^ | 9.07 × 10^−12^ | 1.29 × 10^−6^ | 1.54 × 10^−5^ | 1.72 × 10^−4^ | 1.13 × 10^−3^ |
|  |  |  |  |  | MN535732 |  |  |  |  |  |  |  |  |
| 28 | MK211370 | 7,778–9,724 | ORF1ab | 1,946 | MK211371 | MK211369 | 5.74 × 10^−9^ | 1.28 × 10^−16^ | 1.51 × 10^−18^ | 5.66 × 10^−6^ | 5.23 × 10^−3^ | 3.27 × 10^−9^ | 2.10 × 10^−9^ |
|  |  |  |  |  |  | MK211372 |  |  |  |  |  |  |  |
| 29 | NC028752 | 2,120–4,576 | ORF1ab | 2,456 | MN611517 | AF304460 | 3.38 × 10^−16^ | NS | 4.22 × 10^−15^ | 2.29 × 10^−8^ | 3.78 × 10^−7^ | 1.55 × 10^−8^ | 1.48 × 10^−10^ |
|  |  |  |  |  | KY073748 |  |  |  |  |  |  |  |  |
| 30 | MN611522 | 13,998*–19,588 | ORF1ab | 5,590 | MT747186 | MZ081398 | 9.92 × 10^−5^ | NS | 3.05 × 10^−4^ | 2.68 × 10^−9^ | 7.63 × 10^−12^ | NS | 2.96 × 10^−11^ |
| 31 | EU420138 | 19,804*–26,388 | ORF1ab, ORF3, E, M | 6,584 | MN611524 | Unknown (KJ473796) | 1.34 × 10^−9^ | NS | NS | 7.53 × 10^−12^ | 4.15 × 10^−9^ | 2.34 × 10^−5^ | 2.96 × 10−11 |
| 32 | OK287353 | 24,448–26,195 | ORF3, E, M | 1,747 | Unknown (MG916901) | MZ081388 | NS | 9.23 × 10^−4^ | 1.45 × 10^−6^ | 1.13 × 10^−2^ | 4.08 × 10^−2^ | 3.66 × 10^−5^ | 2.96 × 10^−11^ |
|  |  |  |  |  | Unknown (MK211373) |  |  |  |  |  |  |  |  |
| 33 | OL410609 | 21,791*–24,628 | S | 2,837 | Unknown (OL415262) | MW924112 | 3.64 × 10^−12^ | 2.77 × 10^−21^ | 2.97 × 10^−25^ | 1.41 × 10^−13^ | 1.72 × 10^−8^ | 1.41 × 10^−23^ | 4.60 × 10^−8^ |
| 34 | OL410609 | 4,838–21,114* | ORF1ab, S | 16,276 | Unknown (OL410607) | OL415262 | 4.37 × 10^−3^ | 6.81 × 10^−27^ | 3.33 × 10^−29^ | 1.01 × 10^−9^ | 8.76 × 10^−9^ | NS | 5.93 × 10^−11^ |
| 35 | JQ989266 | 11,333–17,812 | ORF1ab | 6,479 | JQ989270 | JQ989272 | 7.30 × 10^−8^ | 9.85 × 10^−4^ | 6.10 × 10^−11^ | 1.30 × 10^−6^ | 4.19 × 10^−8^ | 7.20 × 10^−7^ | 1.78 × 10^−8^ |
|  |  |  |  |  | JQ989271 |  |  |  |  |  |  |  |  |
| 36 | MH687964 | 19,856*–26,134 | ORF1ab, ORF3, E, M | 6,278 | Unknown (MH687965) | MH687956 | NS | 1.10 × 10^−6^ | 9.04 × 10^−8^ | 2.30 × 10^−4^ | 8.54 × 10^−5^ | 1.58 × 10^−5^ | 5.40 × 10^−5^ |
| 37 | MH687957 | 6,999–9,091 | ORF1ab | 2,092 | Unknown (MH687954) | MH687964 | NS | 3.94 × 10^−7^ | 7.43 × 10^−9^ | 1.42 × 10^−5^ | 7.92 × 10^−5^ | 2.43 × 10^−11^ | 8.74 × 10^−4^ |
| 38 | JQ989272 | 8,774*–10,957 | ORF1ab | 2,183 | JQ989271 | JQ989266 | 3.68 × 10^−3^ | 1.06 × 10^−4^ | 2.58 × 10^−7^ | 9.42 × 10^−6^ | 1.57 × 10^−7^ | 5.69 × 10^−5^ | 3.88 × 10^−6^ |

Recombination event involving a novel AlphaCoV genome identified in this work is depicted in bold.

* = The actual breakpoint position is undetermined (it was most likely overprinted by a subsequent recombination event).

The sequence listed as “unknown” was used to infer the existance of a missing parental sequence.

NS = No significant *p*-value was recorded for this recombination event using this method.
